# PAX-regulated self-organizing polarity drives procambial strand formation

**DOI:** 10.1038/s41477-026-02382-w

**Published:** 2026-08-27

**Authors:** Alkistis E. Lanassa Bassukas, Yao Xiao, Namuk Kim, Claus Schwechheimer

**Affiliations:** 1 Plant Systems Biology, School of Life Sciences, Technical University of Munich, Freising, Germany; 2 Present Address: Guangdong Provincial Key Laboratory of Plant Adaptation and Molecular Design, School of Life Sciences, Guangzhou University, Guangzhou, China; 3 Present Address: Korea Research Institute of Bioscience and Biotechnology, Yuseong-gu, Republic of Korea

**Keywords:** Leaf development, Patterning

## Abstract

In plants, the vascular network enables the transport of water, nutrients and metabolites. The vascular network formed in leaves is a striking example of tissue patterning, and leaf venation is a defining leaf feature. During patterning, veins arise from aligned, elongated procambial cells derived from undifferentiated ground meristem cells. The mechanisms behind procambial strand formation involving cell selection, polarization, alignment, elongation and division have remained elusive. Here we identify a previously uncharacterized role for the polar protein PAX in establishing coordinated cell and tissue polarity during this process. We uncover a novel, auxin-guided role for the polar PAX protein in establishing cell and tissue polarity that precedes and is independent of the polarization and activity of PIN auxin transporters. Our findings refine and redefine existing models of vascular patterning during vein network formation in plant leaves.

## Abstract (web summary)

PAX, a polarly localized protein kinase, converts auxin cues into coordinated cell polarity, propagating polarity between neighbouring cells to align procambial cells and form connected veins. PAX reinforces PIN-mediated auxin patterning and vascular connectivity.

## Main

The establishment of cell polarity is a fundamental process in plants and animals, providing directional cues essential for tissue organization and function^1–3^. How polarity axes are generated to guide tissue patterning and morphogenesis is a central question in developmental biology. In animals, polarity often arises through cell migration and dynamic cell–cell interactions. By contrast, plant cells are constrained by rigid cell walls and must rely on alternative, largely unidentified mechanisms to establish polarity.

In plants, the vascular system, responsible for transporting water, nutrients and metabolites, consists of xylem and phloem. This network forms de novo during leaf development from aligned strands of procambial cells^3–5^. Procambial strand formation begins when ground meristem cells acquire a preprocambial state marked by elevated auxin levels relative to neighbouring cells^3,5^. These cells then differentiate into procambial cells, characterized by sustained high auxin levels and strong expression of the auxin transporter PIN1^3,5^. To form continuous, end-to-end aligned strands, ground meristem cells must be progressively selected, polarized, aligned and induced to divide and elongate anisotropically. However, the molecular mechanisms coordinating these processes have remained unclear.

PAX (PROTEIN KINASE ASSOCIATED WITH BRX) is an AGC1 family serine/threonine kinase closely related to D6 PROTEIN KINASE (D6PK) and the paralogous D6PK-LIKE1–3 (D6PKLs), collectively referred to as D6PK/D6PKLs^6,7^. These kinases are polarly localized plasma membrane-associated proteins that phosphorylate and activate PIN-FORMED (PIN) auxin transporters and regulate auxin-dependent growth^7–9^.

Here, we identify critical auxin-guided but PIN transporter-independent functions of polarized PAX in establishing cell and tissue polarity required for coordinated procambial strand formation during vein and vascular network development^7,10^.

## Results

### PAX is essential for vascular network connectivity

**Fig. 1:**
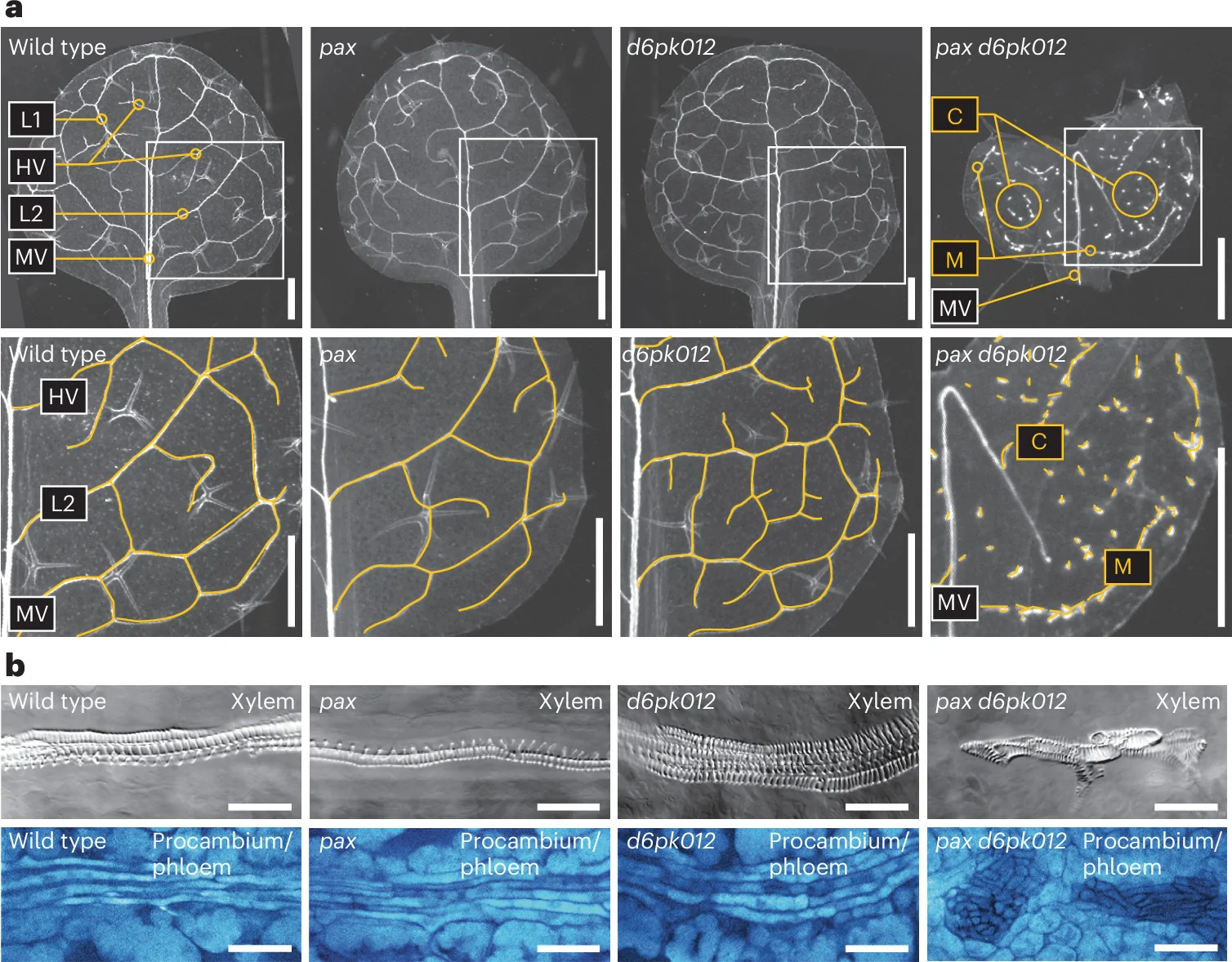
Mutations in *PAX* and *D6PK/D6PKL* disrupt vascular network continuity but not vascular cell differentiation in leaves. **a**, Representative dark-field microscopy images of cleared first leaves from 10-day-old seedlings of the indicated genotypes. White frames (top) denote regions magnified below. The MV, lateral loops (L), HVs, lamina cell conglomerates (C) and leaf margin (M) are indicated; all veins except the MV are false-coloured in yellow. The *pax d6pk012* leaf is partially folded owing to the rigid structure of the leaf; bright, scattered structures are trichomes. Scale bars, 1.5 mm. **b**, Representative confocal microscopy images of L1-stage leaves showing xylem strands (top, transmitted light image) and phloem/procambial tissues (bottom, autofluorescence). Images represent reproduced for at least three biological replicates in at least three independent experiments. Vascular network topology and vascular cell morphology were consistently reproduced in all depicted genotypes across at least two independent experiments, each containing more than three biological replicates. Scale bars, 25 µm.

The mature vascular network of an *Arabidopsis thaliana* leaf consists of a central midvein (MV) connecting the leaf to the rest of the plant (Fig. 1a). Lateral veins branch from the MV to form loops (L1–L3), initially in distal regions and later in proximal regions of the leaf. As the leaf expands, higher-order veins (HVs) emerge and either reconnect to the existing network or terminate freely within the lamina, producing closed or open vein patterns^5,11^. Although the vascular network is not fully predetermined, its topology can be quantified using three indices: the continuity index (fraction of veins with at least one connected end), the connectivity index (fraction of veins connected at both ends) and the cardinality index (number of veins with at least one connected end)^5,11^.

**Extended Data Fig. 1:**
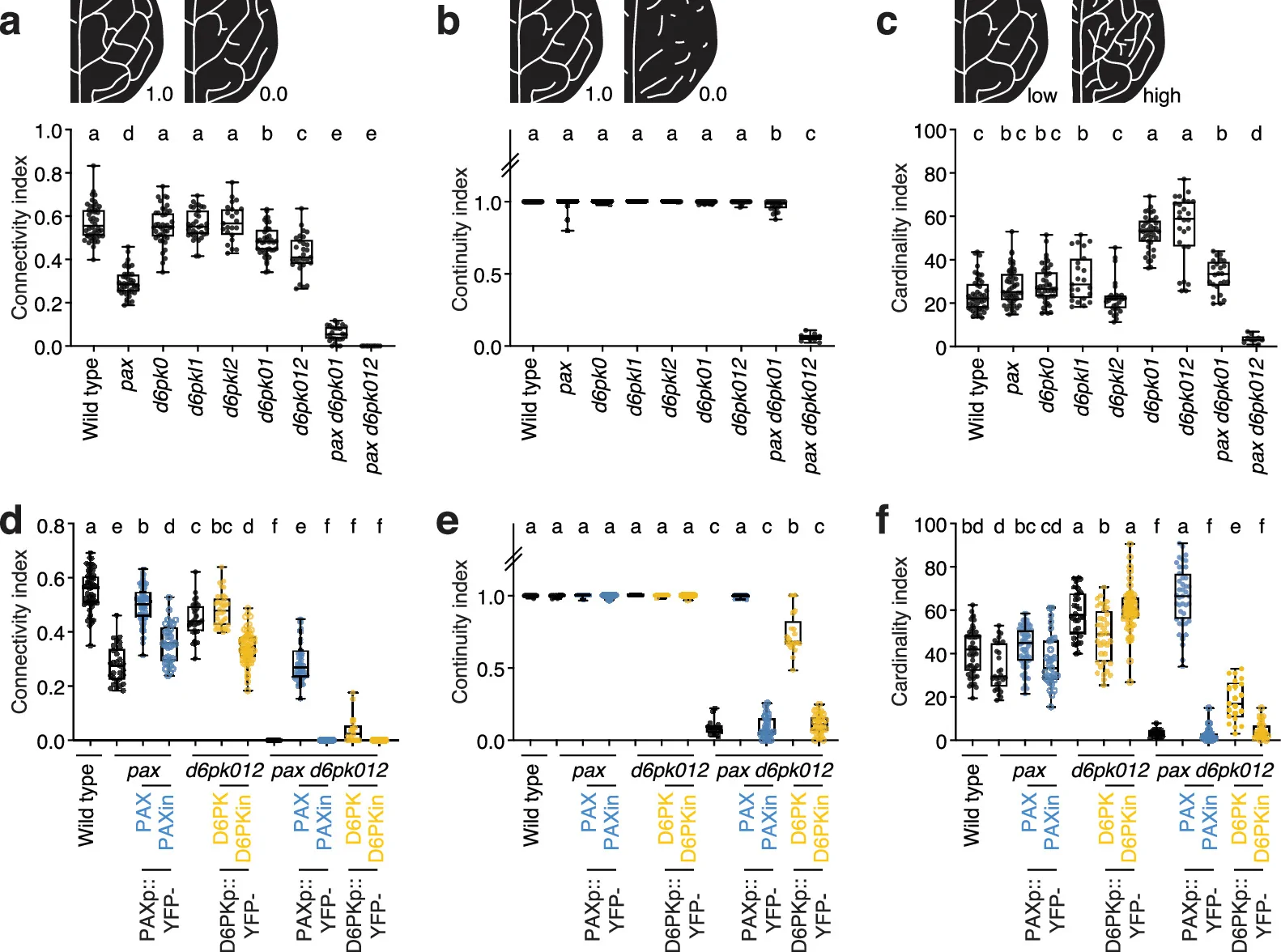
*PAX* and *D6PK/D6PKLs* cooperatively define vascular network topology in *Arabidopsis thaliana* leaves. **(a–f)** Box plots showing individual leaf measurements (dots), median (line), interquartile range (box), and minimum–maximum values (whiskers) of vein connectivity (**a,d**), continuity (**b,e**), and cardinality (**c,f**) indices for the indicated genotypes (see Fig. 1a, Extended Data Fig. 2, and Supplementary Fig. S1). Insets in (**a-c**) illustrate the corresponding network indices adapted from Verna et al.^11^. Sample sizes: *n* = individual leaves. Groups not sharing the same letter (**a–f**) differ significantly (adjusted P < 0.05). Source data

Analysis of 10-day-old *pax* mutants revealed a strong increase in veins connected at only one end, resulting in a markedly reduced connectivity index (Fig. 1a and Extended Data Fig. 1). To assess the relative roles of PAX and D6PK/D6PKLs in vascular network formation, we quantified network topology in single and higher-order mutants. While single *d6pk* and *d6pkl* mutants, as well as *d6pk01* double and *d6pk012* triple mutants, showed only mild connectivity defects, *pax d6pk01* and *pax d6pk012* mutants exhibited severe fragmentation of the vascular network (Fig. 1a, Extended Data Fig. 1 and Supplementary Fig. 1). In *pax d6pk012*, the network was reduced to the MV and isolated clusters of vascular cells scattered throughout the lamina, demonstrating a requirement for these kinases in vascular strand continuity (Fig. 1a, Extended Data Fig. 1 and Supplementary Fig. 1).

Despite the severe loss of connectivity, *pax d6pk012* leaves still contained cells with lignified xylem morphology and cells resembling phloem or procambium, indicating that procambial, xylem and phloem differentiation can occur independently of *PAX* and *D6PK/D6PKLs* (Fig. 1b). The connectivity defects correlated with reduced leaf and plant size, probably reflecting impaired long-distance transport as previously reported for the *pax* mutant^7^ (Supplementary Fig. 1e).

**Extended Data Fig. 2:**
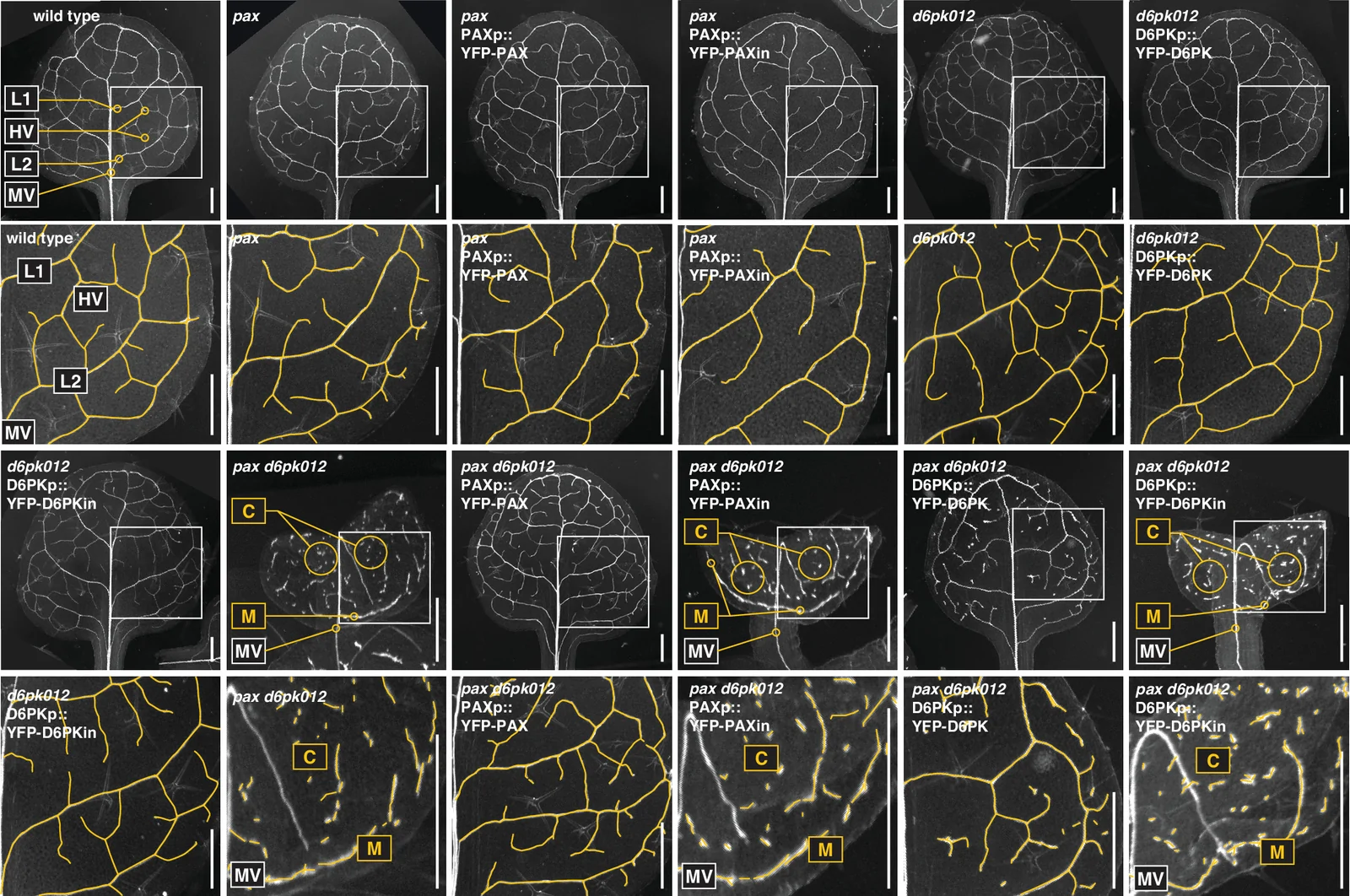
Expression of kinase-active PAX and D6PK suppresses vein topology defects in *pax*, *d6pk012*, and *pax d6pk012* mutants. Representative dark-field microscopy images showing xylem networks in cleared leaves from 10-day-old plants of indicated genotypes. White frames in upper panels indicate magnified regions shown below. The midvein (MV), selected lateral loops (L), higher-order veins (HV), cell conglomerates in the leaf lamina (C), and margin (M) are labeled; all veins except the MV are highlighted in yellow. Quantification of the network topology is presented in Extended Data Fig. 1d–f. Leaves of *pax d6pk012*, *pax d6pk012* PAXp::YFP-PAXin, and *pax d6pk012* PAXp::YFP-D6PKin are partially folded due to their rigid structures; bright, scattered structures are trichomes. All images are from the same experimental series; images for wild type, *pax*, *d6pk012*, and *pax d6pk012* are identical to those in Fig. 1a. Imaging and analysis of vein networks were repeated independently in at least three experiments, each including more than three biological replicates, with similar results obtained for all genotypes. Scale bars, 1.5 mm.

Expression of PAXp::YFP-PAX fully rescued connectivity and continuity defects in *pax d6pk012* (Extended Data Figs. 1 and 2 and Supplementary Fig. 1b–d). D6PKp::YFP-D6PK fully complemented *d6pk012* but only weakly rescued *pax d6pk012*, indicating a dominant role for PAX (Extended Data Figs. 1 and 2). Kinase-inactive variants of PAX and D6PK failed to rescue *pax d6pk012*, although partial activity of inactive PAX was detected in *pax* single mutants (Extended Data Figs. 1 and 2). Thus, kinase activity is required for PAX- and D6PK-mediated vascular patterning.

As PAX and D6PK/D6PKLs activate PIN-mediated auxin transport, we examined whether the observed phenotypes could be explained by reduced auxin transport^7,8^. Increased vein cardinality and broadened vascular tissues, phenotypes previously linked to impaired auxin transport, were observed in *d6pk01* and *d6pk012* mutants and in *pax d6pk012* complemented with PAXp::YFP-PAX^12^ (Extended Data Figs. 1 and 2). However, the pronounced loss of vein connectivity observed in *pax*, in *pax d6pk01* and *pax d6pk012* mutants could not be explained by auxin transport inhibition alone. Thus, while some aspects of the mutant phenotypes are consistent with altered auxin transport, others point to additional functions associated with PAX activity during vascular pattern formation.

Together, these findings indicate that *PAX* is required for proper spatiotemporal patterning of procambial tissue but not for vascular cell differentiation. *PAX* and *D6PK/D6PKLs* cooperate to regulate vein continuity and connectivity but are not functionally redundant. Importantly, PAX has critical functions in connecting the vascular network that cannot be explained by the direct regulation of auxin transport by PAX.

### PAX polarity during procambial cell fate selection is coordinated and follows distinct rules

Vascular networks arise from interconnected procambial strands composed of elongated, axially aligned procambial cells^13^. Procambial strands differentiate from single files of initially isodiametric ground meristem cells that transiently adopt a transmissive cell state, termed preprocambial cells^14^. During vein initiation, broad PIN1 expression domains narrow into discrete auxin-enriched cell files, while PIN1 localization transitions from diffuse to polarized along the strand axis^15^.

**Fig. 2:**
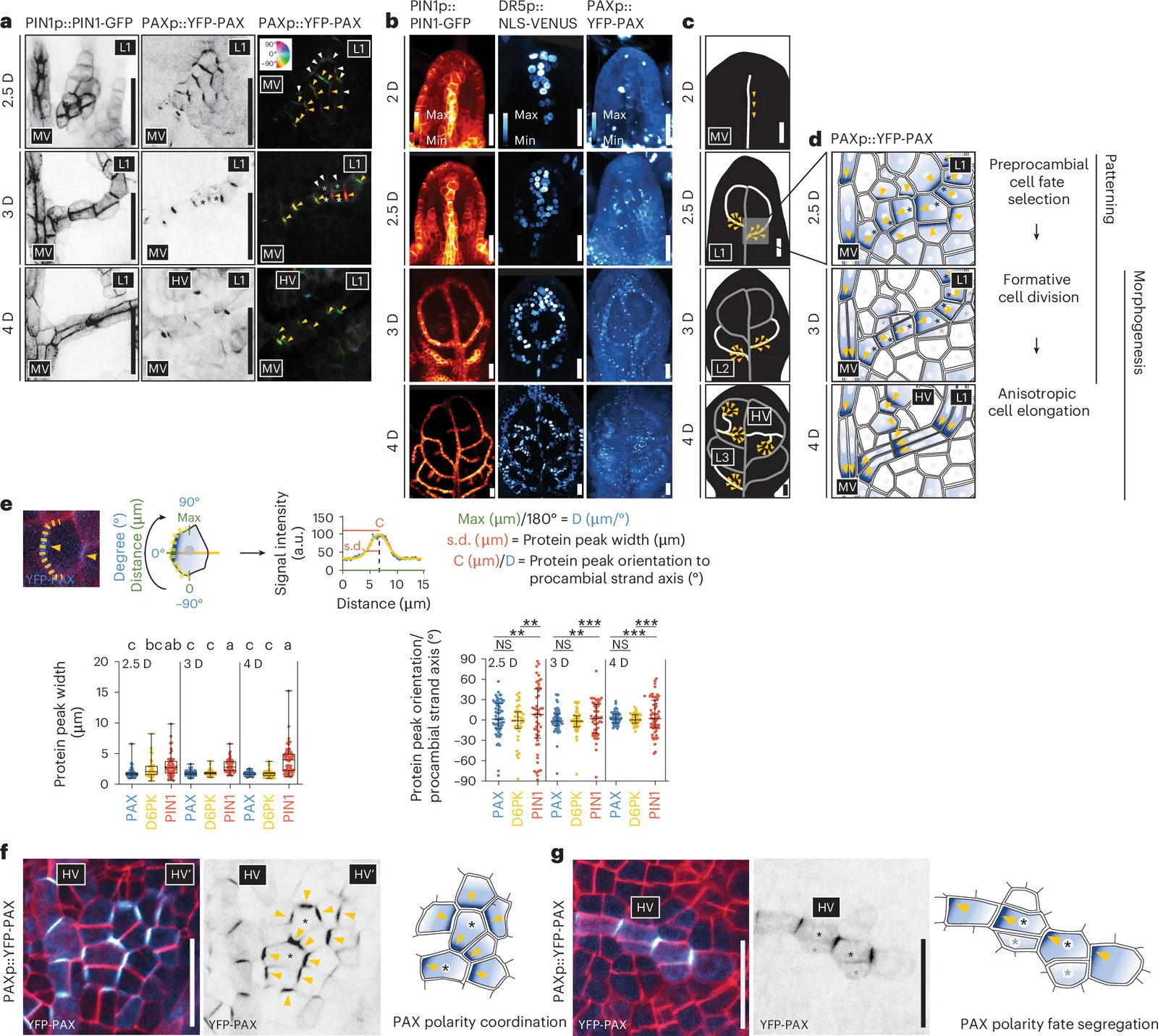
PAX polarity during preprocambial cell fate selection is coordinated and follows distinct rules. **a**, *Z*-projections of L1 leaf primordia at 2.5, 3 and 4 days (D) displayed in inverted greyscale, showing PIN1p::PIN1-GFP (left) and PAXp::YFP-PAX (middle) in incipient procambial strands. Right: colour-coded cellular polarity of PAX as defined in the legend. Yellow arrowheads highlight coordinated cell polarity aligned across three or more consecutive cells. Scale bars, 25 µm. **b**, *Z*-projections of representative confocal images of 2-, 2.5-, 3- and 4-day-old (D) leaf primordia expressing PIN1p::PIN1-GFP (left), the nucleus-targeted auxin reporter DR5p::NLS-VENUS (middle) in wild type or PAXp::YFP-PAX in *pax* mutant background (right). Calibration bars indicating fluorescence intensity. Scale bars, 25 µm. **c**, Graphical representations of tissue polarity derived from PAX cellular polarity shown in **a** and **b**. White lines mark incipient veins; grey lines denote pre-existing veins. Yellow arrowheads indicate tissue polarity. **d**, Schematic of cellular patterns during procambial strand formation, including formative cell divisions and anisotropic elongation. Blue-shaded cells express polar or intracellular PAX; asterisks mark cells recruited into the procambial strand. Yellow arrowheads indicate cell and tissue polarity. MV, lateral loops (L) and HVs (HV, HV′) are labelled in **a**, **c** and **d**. **e**, Box plots showing individual cell measurements (dots), median (line), interquartile range (box) and minimum–maximum values (whiskers) of protein peak width (s.d. of Gaussian fits) as a measure of cell polarity (left), and graphs showing individual cell measurements, median and interquartile range of protein peak orientation relative to the procambial strand axis as a measure of polarity alignment (right). The schematic above illustrates the measured parameters. Individual cells constituted the statistical unit and were sampled from the loop-forming regions of the first lateral loop (L1) at developmental stages 2.5–4 days from at least four independent leaves per genotype and developmental stage. NS, not significant; **P < 0.01; ***P < 0.001; groups not sharing the same letter (**a**–**c**) differ significantly (adjusted *P* < 0.05). **f**,**g**, *Z*-projections of incipient HVs at 4 days expressing PAXp::YFP-PAX in *pax*, shown in colour and inverted greyscale, with graphical representation of PAX polarity coordination (**f**) and polarity fate segregation after formative cell divisions (**g**). Asterisks mark reference cells for oriented PAX polarization (**f**) or cells incorporated into the procambial strand (**g**). Scale bars, 25 µm. Source data

**Extended Data Fig. 3:**
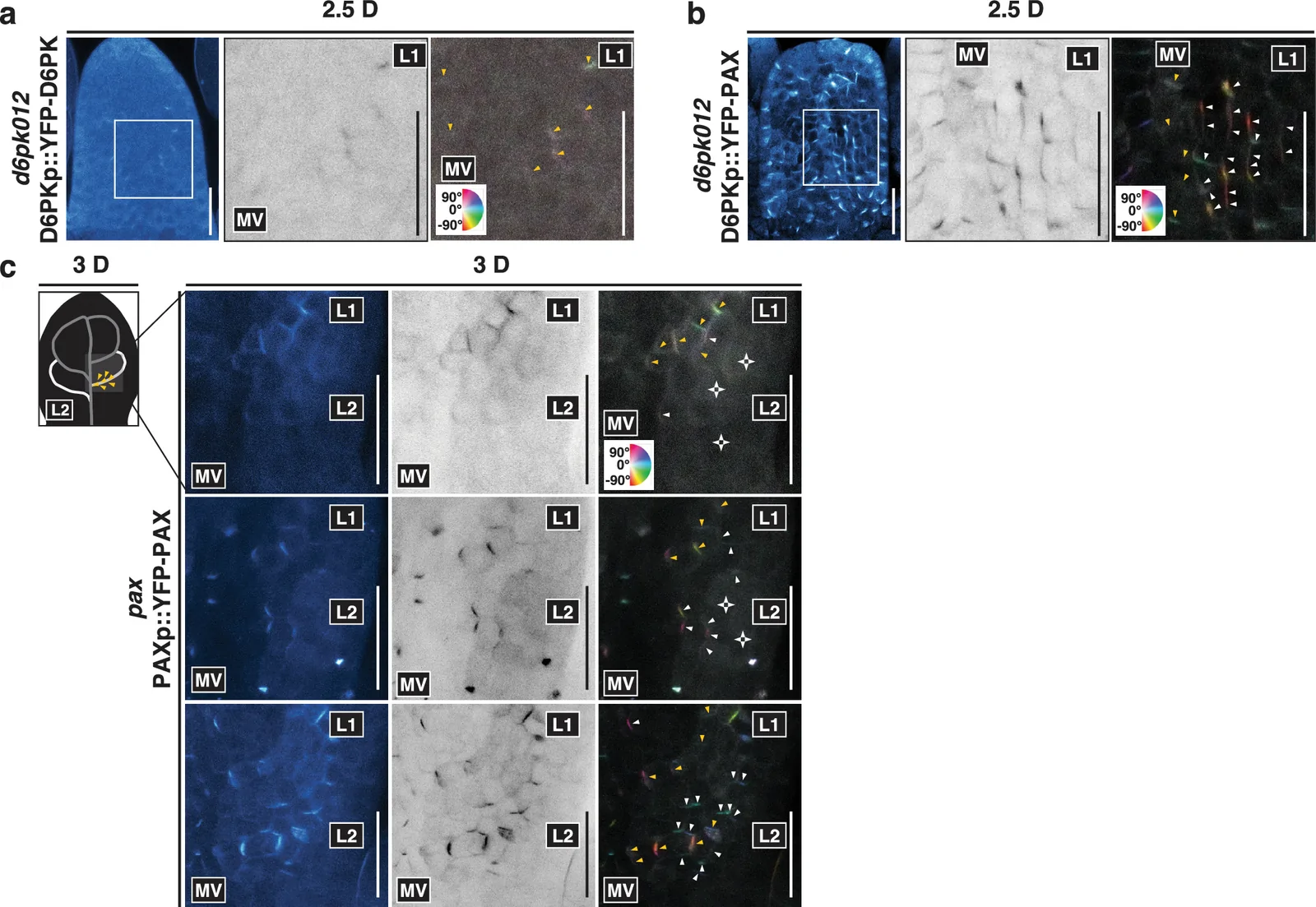
D6PK and PAX exhibit polar distribution in their expression domains. **(a** and **b)** Z-projections of representative confocal scans from 2.5-day-old (D) leaf primordia expressing either (**a**) D6PKp::YFP-D6PK or (**b**) D6PKp::YFP-PAX in the *d6pk012* background. Left panels show color-mode images, middle panels display magnified regions (indicated by white frames) in inverted greyscale, and right panels depict color-coded cellular polarity as defined in the accompanying legend. **(c)** Representative examples illustrating the developmental progression of the PAX polarity domain preceding L2 vein formation. Z-projections of representative confocal scans from different 3-day-old (D) leaf primordia expressing PAXp::YFP-PAX, shown in color (left), inverted greyscale (middle), and polarity-coded mode (right). to illustrate the propagation of PAX polarity prior to procambial cell fate specification. The upper row shows an early stage characterized by a broad PAX expression domain lacking pronounced polar localization. In the middle row, cells proximal to the midvein exhibit coordinated polarity and appear to function as polarity attractors for more distally positioned neighboring cells. In the lower row, multiple partially overlapping PAX polarity trajectories converge on a proximally positioned cell near the midvein, forming a supracellular fan-shaped polarity front domain. Yellow arrowheads indicate coordinated polarity, where D6PK or PAX polarity aligns in at least three consecutive cells. White arrowheads indicate polarity in neighboring cells not necessarily acquiring procambial identity. The midvein (MV), lateral loop 1 (L1), and lateral loop 2 (L2) are indicated. Scale bars, 25 µm.

To examine the dynamics of PAX distribution during procambial patterning, we analysed PAX localization during L1 loop formation in first leaf primordia at 2–4 days, together with PIN1 and the auxin reporter DR5::NLS-VENUS. At 2–2.5 days, PAX was broadly expressed along the MV and in flanking ground meristem domains marking future L1 veins, similar to PIN1 (Fig. 2a–e). While D6PK expression was uniform throughout the ground meristem (Extended Data Fig. 3a), PAX expression, similar to PIN1, was restricted to distinct domains flanking the developing MV, each spanning several ground meristem cells in diameter and delineating the future L1 region (Fig. 2a–d). Comparable broad PAX expression domains were observed in developing HVs (L2 and HV) (Extended Data Fig. 3b,c).

By 2.5 days, PAX and D6PK already showed strong polarized plasma membrane localization in a fan-shaped group of cells around the incipient procambial axis, whereas PIN1 remained largely diffuse (Fig. 2a–e). By 3 days, PAX expression became restricted to narrow preprocambial strands, parallelling PIN1 and auxin marker refinement (Fig. 2a–e).

**Extended Data Fig. 4:**
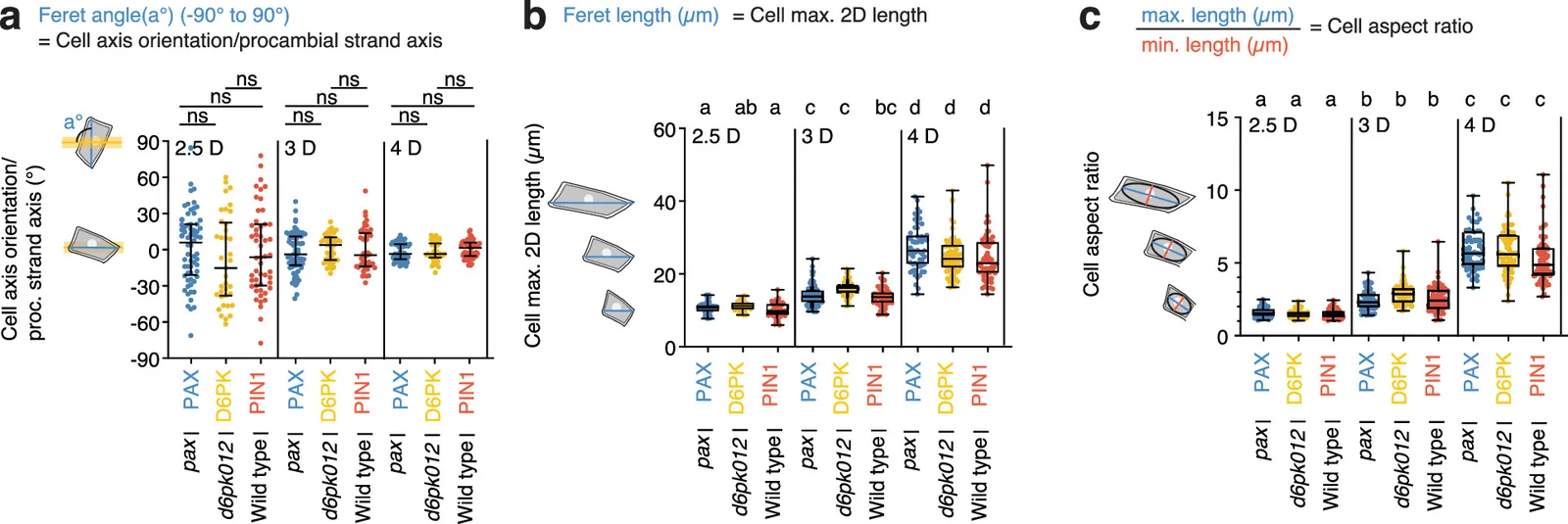
Cell morphology is unaffected by expression of the PAX, D6PK, or PIN1 transgenes. **(a)** Graph showing individual measurements, medians, and interquartile ranges of cell axis orientation relative to the axis of the procambial strand (Feret angle). **(b,c)** Box plots showing individual cell measurements (dots), median (line), interquartile range (box), and minimum–maximum values (whiskers) of maximum cell length (Feret length; **b**) and cell aspect ratio (maximum/minimum cell length; **c**). Schematic illustrations next to each graph indicate the cellular parameters used for each calculation. Statistical analyses were performed on individual YFP–PAX-, YFP–D6PK-, and PIN1–GFP-expressing cells sampled from the loop-forming region of the first lateral loop (L1) at developmental stages 2.5–4 D from at least four independent leaves per genotype and developmental stage. In (**a**), ns, not significant. In (**b,c**), groups not sharing the same letter (**a–c**) differ significantly (adjusted *P* < 0.05). Source data

D6PK was broadly expressed across the ground meristem and did not show the expression-domain refinement observed for PAX; however, D6PK polarity coincided spatiotemporally with PAX, being restricted to sites of incipient strand formation (Extended Data Fig. 3a). Expressing PAX under the control of the *D6PK* promoter expanded the PAX polarity domain beyond its native expression domain, revealing that ground meristem cells are competent to polarize PAX and that transcriptional deployment of PAX is a key determinant of its spatial polarity pattern (Extended Data Fig. 3b). Expression of these transgenes did not per se interfere with cell differentiation and affect cell morphology (Extended Data Fig. 4). Thus, PAX and D6PK polarity emerge early in morphologically preprocambial cells, preceding PIN1 polarization and procambial differentiation.

During our observations, we found that the formation of PAX cell and tissue polarity reproducibly followed several consistent rules: (1) polarized PAX was only observed in cells contacting other polarized cells (Fig. 2a–f and Extended Data Fig. 3b,c); (2) polarity orientation was coordinated among neighbouring cells, often towards an existing polarized strand such as the MV (Fig. 2a–f and Extended Data Fig. 3b,c); (3) PAX localization was restricted to plasma membrane regions at cell–cell interfaces shared with other polarized cells (Fig. 2f); and (4) after formative divisions, polarized PAX was often retained in only one daughter cell incorporated into the strand, suggesting polarity segregation (Fig. 2g). Together, these findings reveal that PAX expression dynamics are decisive for the establishment of PAX polarity patterns within the growing tissue and that PAX polarity is contingent on cell position rather than cell ontogeny. Further, the possibility of a PAX polarity segregation may help sharpen the lateral boundary of the PAX-polarized cellular field and of the forming procambial strand.

### PAX-dependent cell polarization is independent of PIN-mediated auxin transport

PIN1 distribution and auxin transport are essential for establishing proper vein topology and determining PIN1 expression domains but not for vein formation^11,12^. Mechanisms underlying the coordinated cell polarization downstream of auxin signalling during procambial strand patterning and cell morphogenesis remain elusive. To examine the dependency of PIN1 polarity on PAX and D6PK/D6PKLs, we analysed wild-type and *pax d6pk012* leaves using antibodies detecting PIN1 and its phosphorylated (active) forms^8,16^ (Supplementary Fig. 2).

**Fig. 3:**
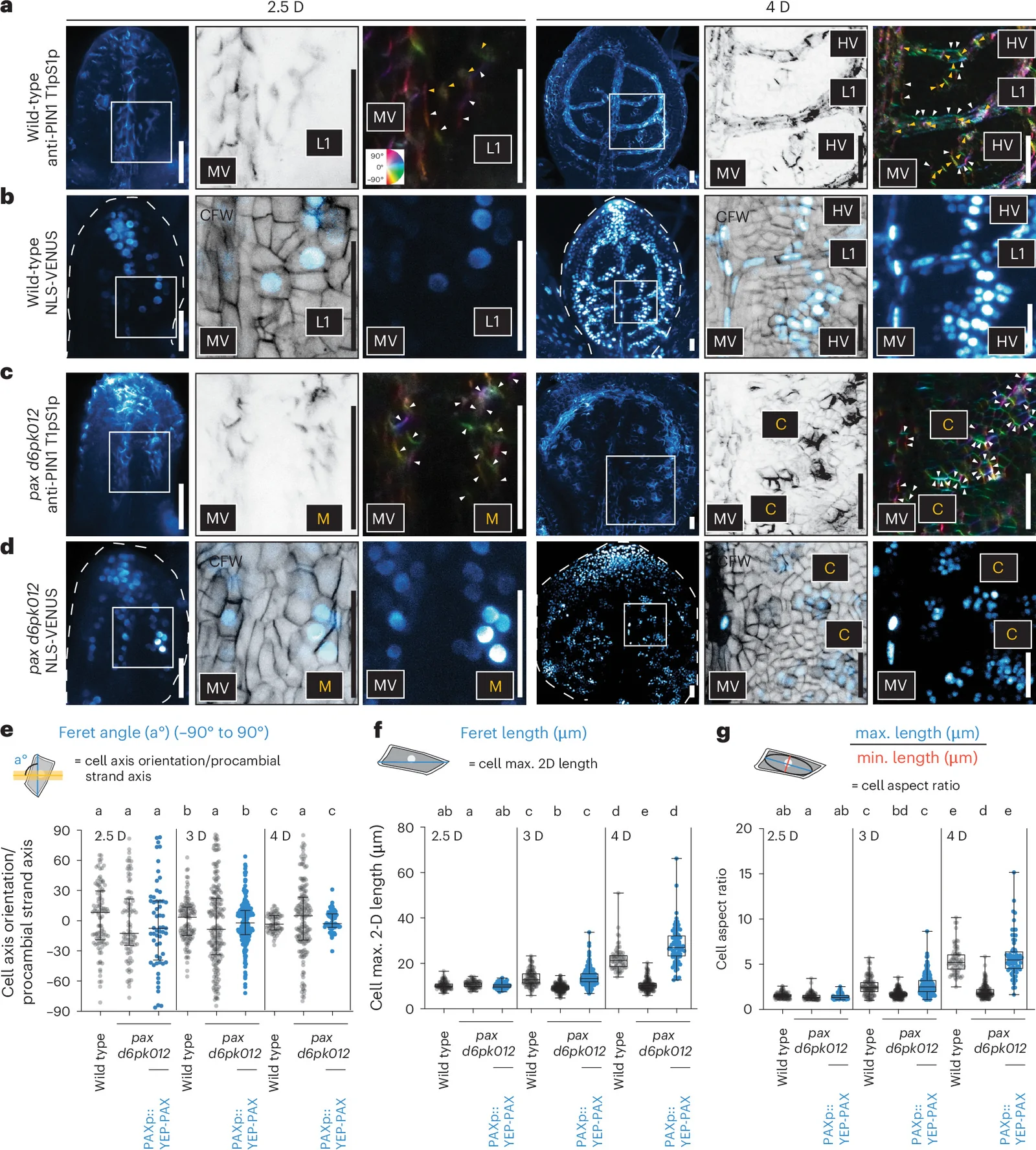
Cell polarization during procambial strand formation depends on PAX and is independent of PIN-mediated auxin transport. **a**–**d**, *Z*-projections of representative confocal images of 2.5- and 4-day-old (D) leaf primordia from wild type (Col-0) and *pax d6pk012* mutants. Samples were immunostained with anti-PIN1 T1pS1p (**a** and **c**) or expressed the nucleus-targeted auxin reporter DR5p::NLS-VENUS (**b** and **d**). Middle: CFW-stained cell walls (inverted greyscale). Right: the NLS-VENUS signal or colour-coded PIN1 polarity orientation. Magnified insets of framed regions (left) are shown. Yellow arrowheads indicate regions where at least three consecutive cells share PIN1 polarity direction; white arrowheads mark other cells with PIN1 polarity. Dashed lines in **b** and **d** outline the leaf lamina. The MV, lateral loops (L), HV, vascular fragments in the margin (M) and cell conglomerates (C) are labelled. See Extended Data Fig. 5 for additional developmental stages. Scale bars, 25 μm. **e**–**g**, Graph shows individual cell measurements, median and interquartile range of cell-axis orientation relative to the procambial strand axis (Feret angle) (**e**). Box plots show individual cell measurements (dots), median (line), interquartile range (box) and minimum–maximum values (whiskers) of maximum cell length (Feret length) (**f**) and cell aspect ratio (maximum/minimum cell length) (**g**). Statistical comparisons were based on individual DR5p::NLS-VENUS-expressing cells counterstained with CFW to visualize cell walls. Cells were sampled from the loop-forming region of the first lateral loop (L1) in wild-type and PAXp::YFP–PAX *pax d6pk012* leaves or, in *pax d6pk012* leaves, from incipient vascular fragments at the leaf margin (M) at developmental stages 2.5–4 days. Groups not sharing the same letter (**a**–**e**) differ significantly (adjusted *P* < 0.05). Source data

**Extended Data Fig. 5:**
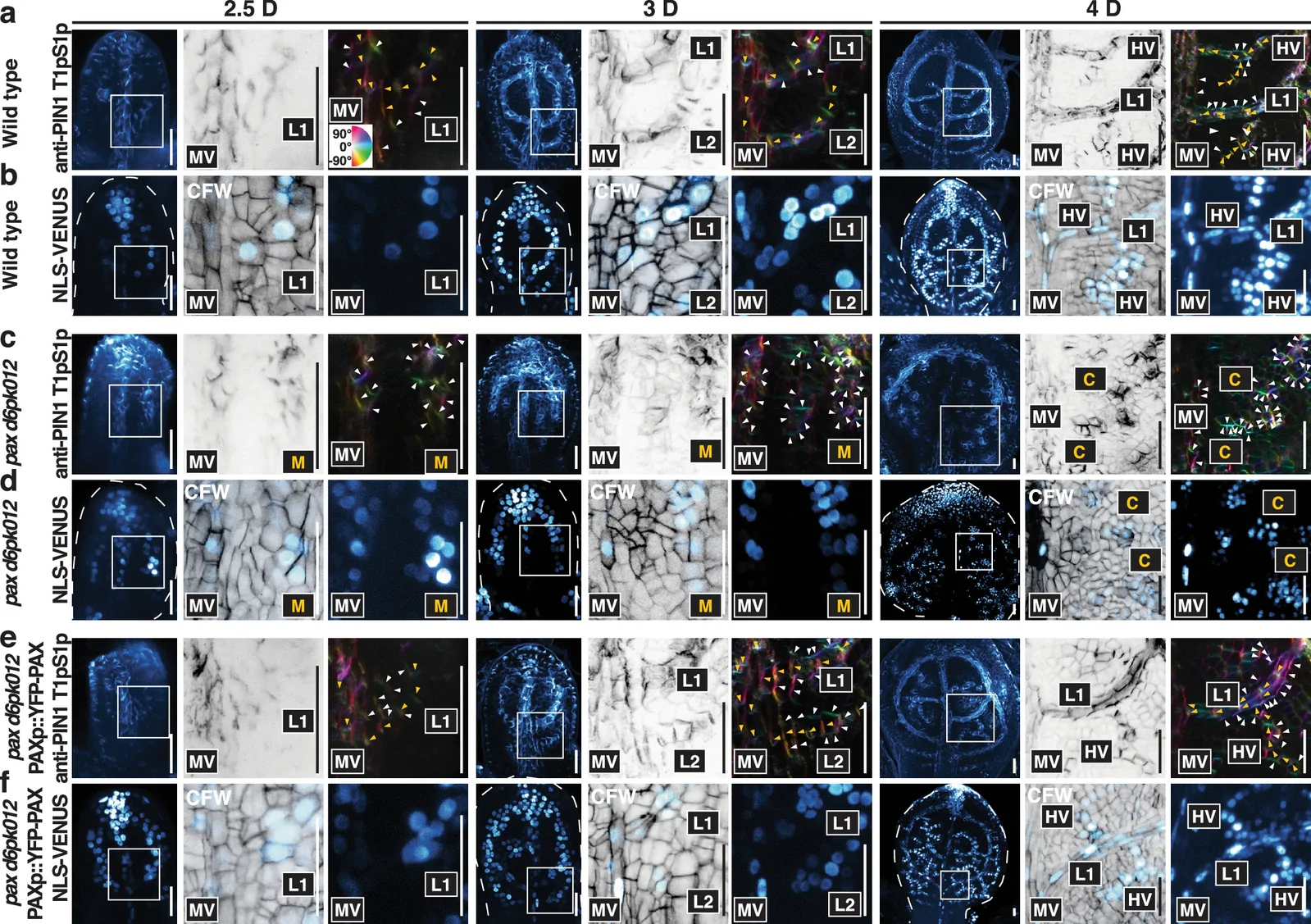
PAX is required for *de novo* PIN1 tissue polarity and auxin patterning in developing leaf primordia (1). **(a–f)** Z-projections of representative confocal scans of 2.5-, 3-, and 4-day-old (D) leaf primordia from wild type (Col-0), *pax d6pk012*, and PAXp::YFP-PAX *pax d6pk012* plants. Samples were immunostained with anti-PIN1 T1pS1p (**a, c, e**) or imaged for DR5p::NLS-VENUS nuclear signal (**b, d, f**) combined with calcofluor white (CFW) cell wall counterstain (middle panels) or shown as NLS-VENUS signal alone (right panels). Magnifications of framed areas (left panels) are shown in inverted grayscale (middle panels) or colour-coded for PIN1 polarity orientation (**a, c, e**) or NLS-VENUS (**b, d, f**). Yellow arrowheads indicate cell polarity, with at least three consecutive cells sharing PIN1 polarity direction; white arrowheads indicate polarity in other cells. Dashed lines mark the leaf circumference where applicable. Midvein (MV), lateral loops (L), higher-order veins (HV), cell conglomerates (C), and cell conglomerates in the leaf margin (M) are labeled. The images from wild type and *pax d6pk012* samples shown for 2.5 and 4 D in panels **a–d** are identical to those shown in Fig. 3a–d.

**Extended Data Fig. 6:**
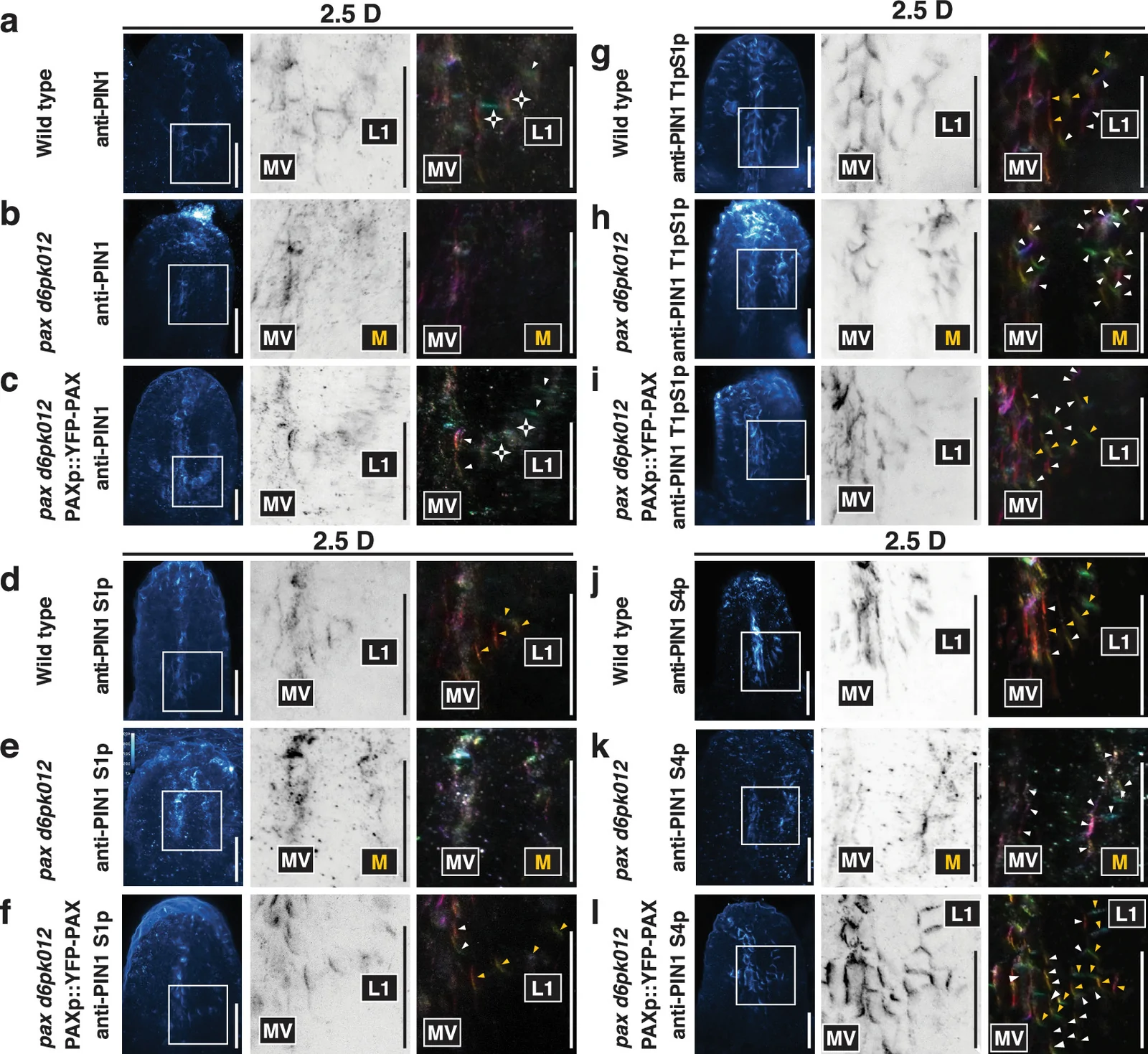
PAX is required for *de novo* PIN1 tissue polarity and auxin patterning in developing leaf primordia (2). **(a–l)** Z-projections of representative confocal scans of 2.5-day-old (D) leaf primordia from wild type (Col-0), *pax d6pk012*, and PAXp::YFP-PAX *pax d6pk012* plants immunostained with anti-PIN1 (**a–c**), anti-PIN1 S1p (**d–f**), anti-PIN1 T1pS1p (**g–i**), or anti-PIN1 S4p (**j–l**). Magnifications of the framed areas (left panels) from full-size images are shown in inverted grayscale (middle panels) or with colour-coded PIN1 polarity orientation (right panels). Yellow arrowheads indicate cell polarity, with at least three consecutive cells sharing PIN1 polarity direction; white arrowheads indicate polarity in other cells; asterisks mark non-polar PIN1 distribution. Midvein (MV), lateral loop 1 (L1), and cell conglomerates in the leaf margin (M) are labeled. Scale bars, 25 µm.

In wild-type leaves, phosphorylated PIN1 exhibited coordinated polarity that corresponded with PAX and D6PK polarities in auxin-accumulating preprocambial cells of the same stage (Fig. 3a,b and Extended Data Figs. 5 and 6). In *pax d6pk012* mutants, phosphorylated PIN1 was mislocalized or polarized towards isolated auxin-rich cell clusters rather than forming aligned strands (Fig. 3c,d). Expression of PAXp::YFP-PAX in *pax d6pk012* restored coordinated PIN1 polarity and strand formation, highlighting the essential role of PAX in coordinating PIN1 polarity across neighbouring cells and, consequently, shaping auxin distribution patterns along discrete and interconnected preprocambial strands (Extended Data Figs. 5 and 6).

Despite mispatterning, phosphorylated PIN1 was still detectable in *pax d6pk012*, suggesting that other kinases contribute to PIN activation^9,16,17^. Morphological analyses showed that procambial cells in wild-type and rescued lines were elongated and axially aligned, whereas *pax d6pk012* cells remained small, isotropic and randomly oriented (Fig. 3e–g). Thus, PAX is required for the axial morphogenesis driving procambial strand formation.

**Extended Data Fig. 7:**
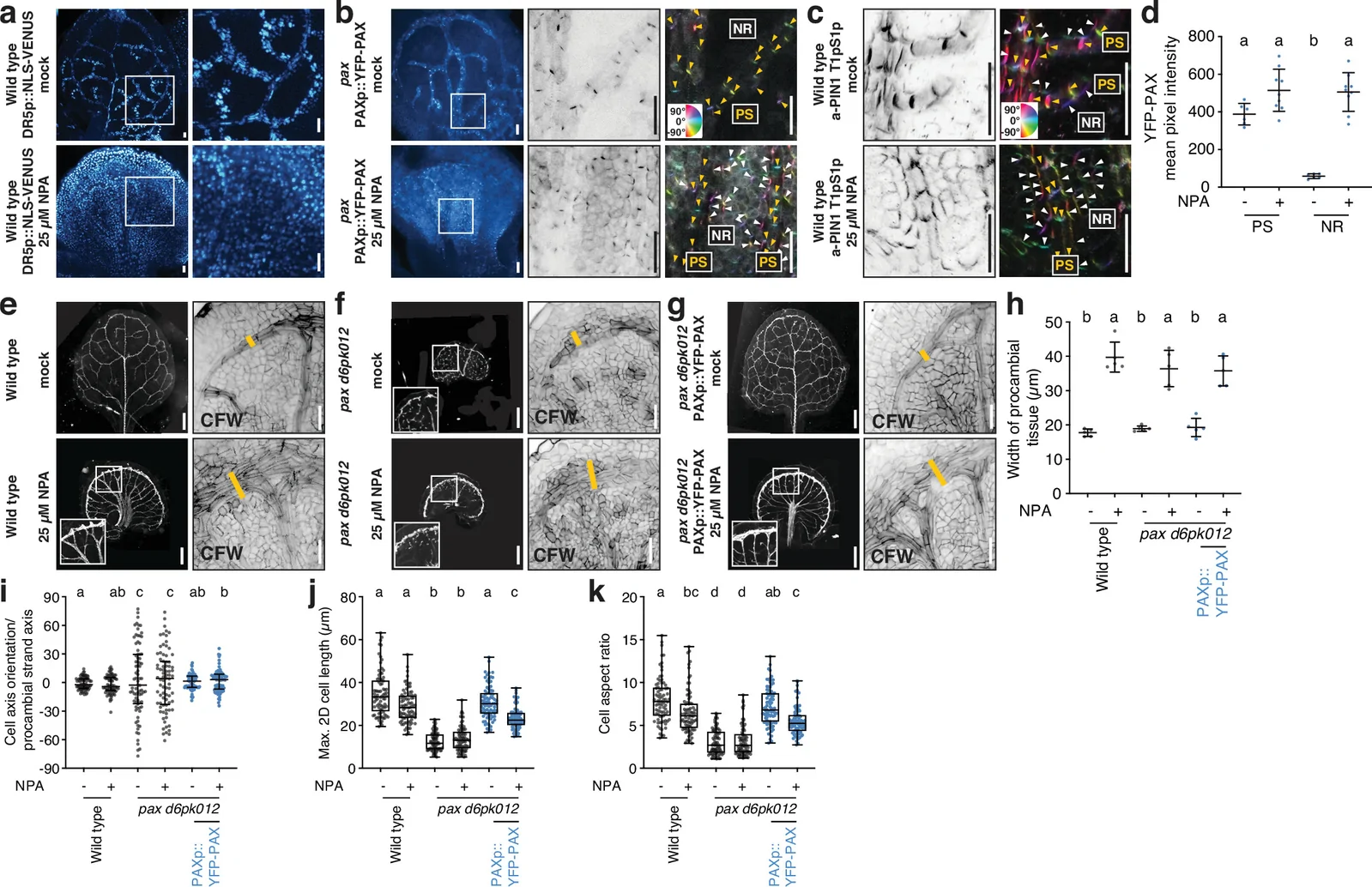
Cell polarisation during procambial strand formation requires PAX but is independent of PIN-mediated auxin transport. **(a)** Z-projected confocal images of 5-day-old leaf primordia expressing DR5p::NLS-VENUS, grown on 25 µM NPA or the mock control. Magnified views of the framed regions (left panels) are shown at right. Scale bars, 25 µm. **(b** and **c)** Z-projections of confocal images of 5-day-old PAXp::YFP-PAX pax leaf primorida (**b**) and 3-day-old wild-type immuno-stained with anti- PIN1 T1pS1p leaf primordia (**c**) grown on 25 µM NPA or the mock control. In (**b**) magnified views of the framed regions (left panels) are shown in inverted grayscale (middle panels) and with colour-coded vectors indicating PAX and polarity orientation (right panels) and in (**c**) inverted grayscale (left panels) and with colour-coded vectors indicating anti- PIN1 T1pS1p polarity orientation (right panels). Yellow arrowheads indicate polarity aligned across at least three consecutive cells; white arrowheads mark polarity in individual or non-aligned cells. Cells of the procambial strand (PS) and neighbouring region (NR) are labeled. **(d)** Quantification of mean YFP–PAX pixel intensity in PS and NR cells of 5-day-old leaf primordia grown with or without NPA. Each dot represents one leaf (, with the value calculated as the mean pixel intensity of 10 background-subtracted regions of interest (ROIs; technical replicates). Data are presented as mean ± s.d. Sample sizes: mock: PS, *n* = 6; NR, *n* = 6. NPA: PS, *n* = 9; NR, *n* = 9; individual leaves. Groups not sharing the same letter (**a–b**) differ significantly (adjusted *P* < 0.05). **(e-g)** Representative dark-field images of 10-day-old leaves (left panels) and inverted grayscale images of the marginal zone from 5-day-old leaves stained with calcofluor white to visualize cell walls (right panels), following NPA (bottom) or mock (top) treatment. Insets show magnified views of the framed regions. Yellow lines in 5-day-old laminae indicate procambial width measurements used in (H). **(h)** Quantification of procambial tissue width in 5-day-old seedlings treated with NPA or mock. Each dot represents one leaf (*n* = 5 leaves per group), with the value calculated as the mean of five width measurements (technical replicates). Groups not sharing the same letter (**a,b**) differ significantly (adjusted *P* < 0.05). **(i-k)** Graph in (**i**) showing individual cell measurements, median, and interquartile range of cell axis orientation relative to the procambial strand axis (Feret angle). (**j,k**) Box plots showing individual cell measurements (dots), median (line), interquartile range (box), and minimum–maximum values (whiskers) of maximum cell length (Feret length; **j**) and cell aspect ratio (maximum/minimum cell length; **k**). Schematics for these parameters are shown in Fig. 3e–g. Sample sizes (*n* = 80 cells per genotype and treatment of four independent leaves). Groups not sharing the same letter (**a-d**) differ significantly (adjusted *P* < 0.05). Source data

The detection of phosphorylated (activated) PIN1 in *pax d6pk012* is suggestive of active auxin transport in the tissue (Fig. 3 and Extended Data Figs. 5 and 6). To test whether these defects were caused by impaired auxin transport, we treated seedlings with the PIN inhibitor *N*-1-naphthylphthalamic acid (NPA)^12,18^. NPA did not affect PAX polarity or PIN1 phosphorylation but broadened PAX and PIN1 expression domains and induced marginal vein expansion, as previously reported^12^ (Extended Data Fig. 7). Importantly, *pax d6pk012* failed to form aligned veins in our NPA treatment conditions, and cell elongation defects persisted. We conclude from our observations that, although PAX regulates the coordination of phosphorylated PIN1 polarity and thereby influences auxin distribution, aspects of the *pax d6pk012* phenotype, particularly the loss of vascular cell axialization, cannot be accounted for by auxin transport defects.

### PDK1 regulates PAX and D6PK polarity

**Fig. 4:**
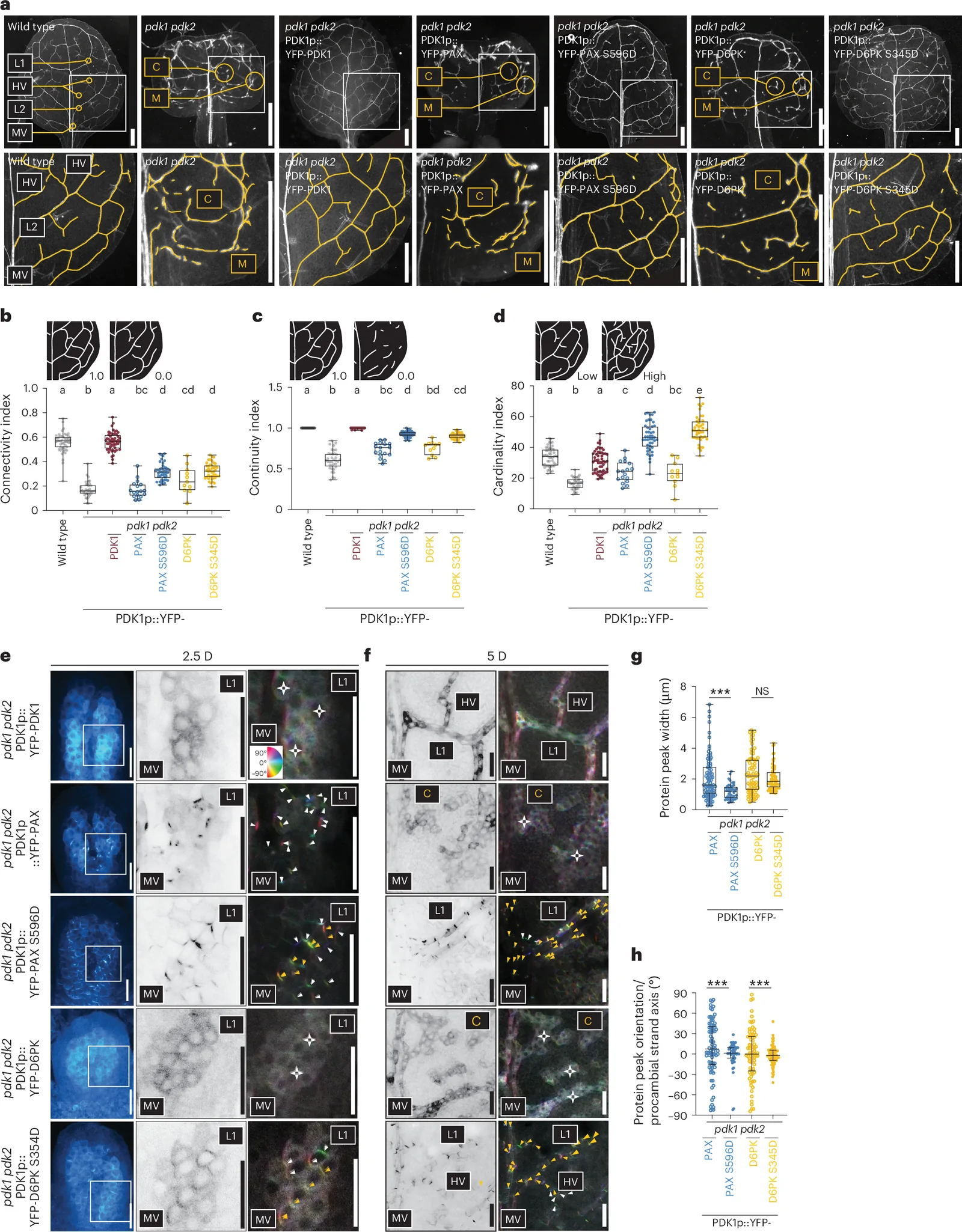
PDKs enhance PAX and D6PK cellular polarity during procambial strand formation. **a**, Representative dark-field microscopy images of the xylem network in cleared leaves from 10-day-old plants of indicated genotypes. White frames (top) highlight magnified areas shown below. MV, selected lateral loops (L), HVs, cell conglomerates (C) and marginal cell conglomerates (M) are labelled; all veins except MV are highlighted in yellow. Other prominent whitish structures are trichomes. Scale bars, 1.5 mm. **b**–**d**, Box plots showing individual leaf measurements (dots), median (line), interquartile range (box) and minimum–maximum values (whiskers) of vein connectivity (**b**), continuity (**c**) and cardinality (**d**) indices for the genotypes shown in **a**. Insets depict graphical representations of the indices (adapted from ref. ^11^). Statistical analyses were performed on individual leaves (*n* = leaves). Groups not sharing the same letter (**a**–**d**) differ significantly (adjusted *P* < 0.05). **e**, *Z*-projections of representative confocal scans of 2.5-day-old (D) leaf primordia from *pdk1 pdk2* expressing PDK1p::YFP-PDK1, PDK1p::YFP-PAX, PDK1p::YFP-PAX S596D, PDK1p::YFP-D6PK or PDK1p::YFP-D6PK S345D. Magnified areas (left) are shown in inverted greyscale (middle) and colour coded for protein polarity orientation (right). Yellow arrowheads indicate polarity in at least three consecutive cells; white arrowheads indicate polarity in other cells. MV and lateral loop 1 (L1) are labelled. Scale bars, 25 µm. **f**, *Z*-projections of 5-day-old (D) leaf primordia from the genotypes in **e** shown in inverted greyscale (left) and colour coded for polarity orientation (right) to assess cell-to-cell alignment during procambial strand formation. MV, lateral loops (L), HVs and cell conglomerates (C) are labelled. Scale bars, 25 µm. **g**,**h**, Quantification of YFP–PAX and YFP–D6PK polarity in the first lateral loop (L1) of 4-day-old leaves or, in *pdk1 pdk2* leaves expressing PDK1p::YFP–PAX or PDK1p::YFP–D6PK, in vascular fragments at the leaf margin. Box plots showing individual cell measurements (dots), median (line), interquartile range (box) and minimum–maximum values (whiskers) of protein peak width (s.d. of Gaussian fits) (**g**). Graphs showing individual cell measurements, median and interquartile range of protein peak orientation (**h**). Sample sizes: *n* = individual cells. adjusted ***P < 0.001.

PDK1 and PDK2 act upstream of PAX and D6PK/D6PKLs by phosphorylating their activation loops^19–21^. The *pdk1 pdk2* mutant exhibited fragmented vein networks with reduced connectivity, resembling but being milder than *pax d6pk012*^19^ (Figs. 1 and 4a–d). Expression of PDK1p::YFP-PDK1 rescued this phenotype (Fig. 4a–d).

Only phosphomimetic versions of PAX (S596D) or D6PK (S345D), but not wild-type proteins, partially rescued *pdk1 pdk2*, indicating that activation loop phosphorylation is required for function (Fig. 4a–d). In *pdk1 pdk2*, expressing wild-type PAX and D6PK lacked coordinated polarity, whereas expression of the phosphomimetic variants restored polarity, as well as PIN1 alignment and cell axialization (Fig. 4e–h). PDK1 itself was broadly and diffusely expressed, consistent with a permissive role of an upstream regulator (Supplementary Fig. 3).

**Extended Data Fig. 8:**
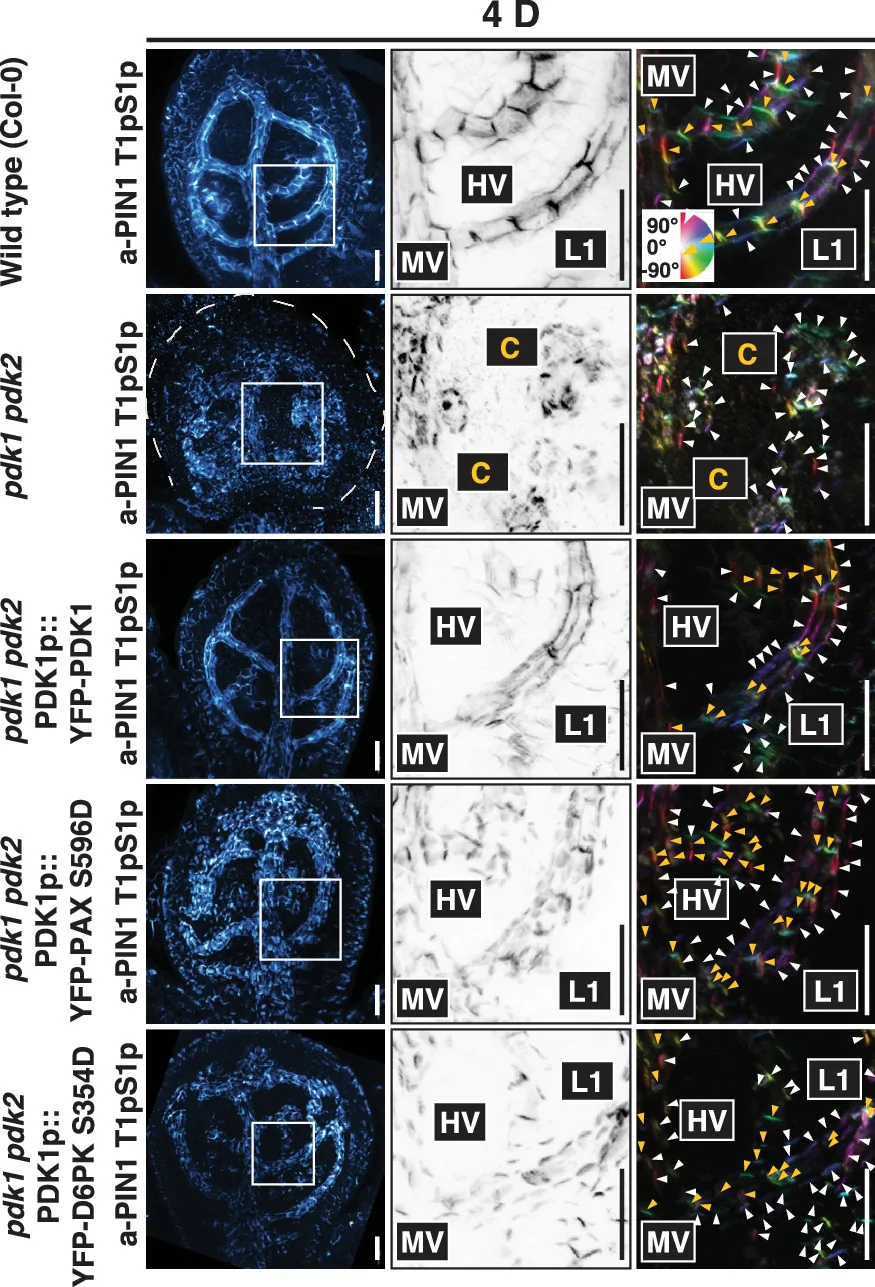
PDK1-dependent activation of PAX and D6PK enhances PIN1 polarity coordination. Z-projections of representative confocal scans of 4-day-old (D) leaf primordia from wild type (Col-0), *pdk1 pdk2*, PDK1p::YFP-PDK1 *pdk1 pdk2*, PDK1p::YFP-PAX S596D *pdk1 pdk2*, and PDK1p::YFP-D6PK S345D *pdk1 pdk2* immunostained with anti-PIN1 T1pS1p. Magnified views of framed areas (left panels) are shown in inverted grayscale (middle panels) and with colour-coded PIN1 polarity orientation (right panels). Yellow arrowheads indicate polarity in at least three consecutive cells; white arrowheads mark polarity in other cells. Midvein (MV), lateral loop 1 (L1), higher-order vein (HV), and cell conglomerates (C) are labeled. Scale bars, 25 µm.

Consistently, the plasma membrane distribution of phosphorylated PIN1 was unoriented in *pdk1 pdk2* but regained polarized distribution when PDK1, PAX S596D or D6PK S345D was expressed in *pdk1 pdk2* (Extended Data Fig. 8). At the level of cell morphology, *pdk1 pdk2* showed impaired axialization, with reduced cell lengths and aspect ratios, defects reversed only by the expression of phosphomimetic PAX S596D or D6PK S345D but not of their wild-type counterparts (Supplementary Fig. 4).

These results demonstrate that PDK1-mediated activation loop phosphorylation promotes coordinated PAX and D6PK polarity, PIN1 axialization and downstream procambial strand patterning and consolidation.

### PAX and D6PK interdependently regulate protein and cell polarity

**Extended Data Fig. 9:**
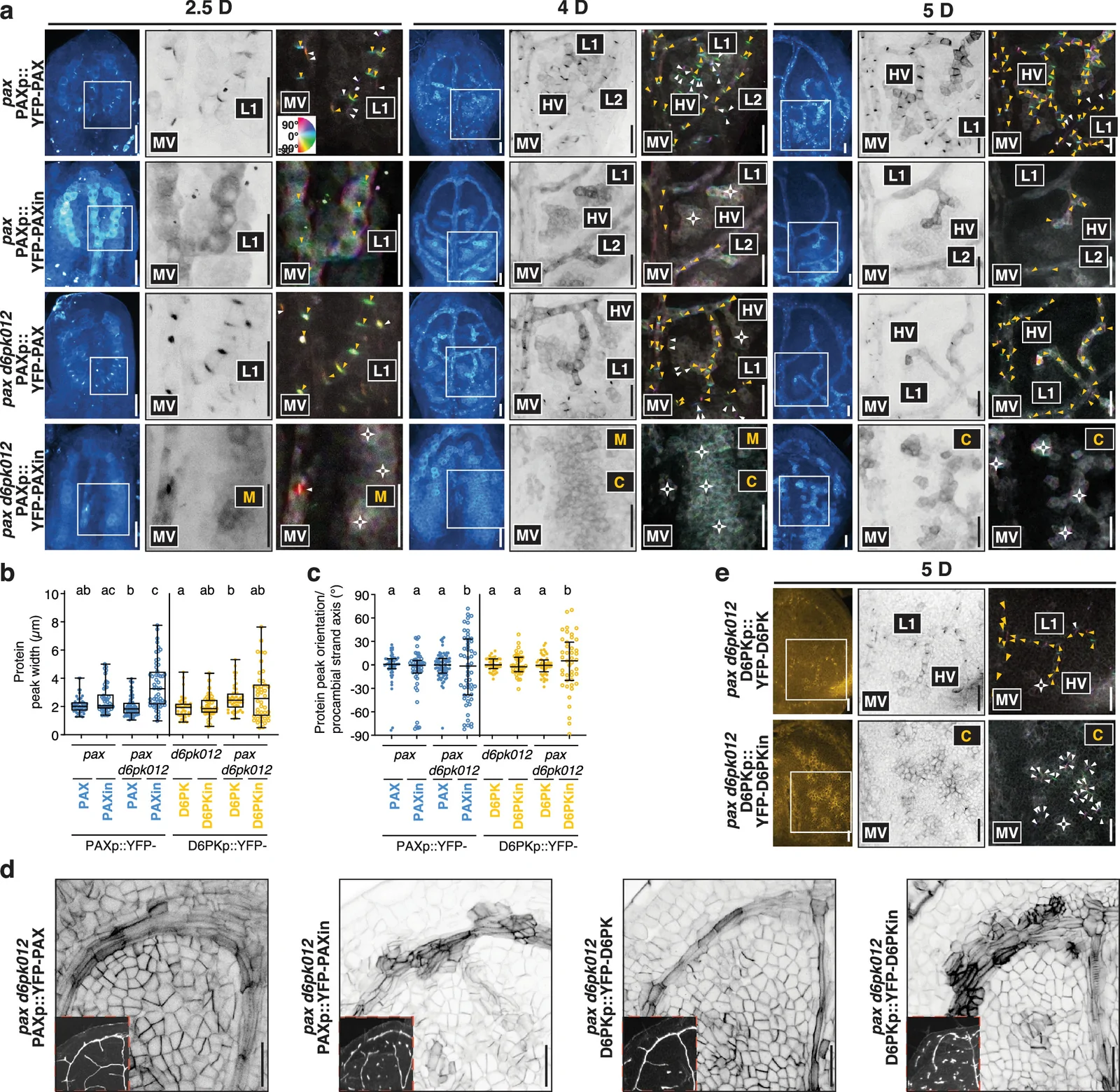
PAX and D6PK/D6PKL kinases function interdependently to coordinate protein polarity. **(a)** Z-projections of representative confocal scans of 2.5-, 4-, and 5-day-old (D) leaf primordia from *pax* or *pax d6pk012* mutants expressing PAXp::YFP-PAX or PAXp::YFP-PAXin. Magnified views of framed areas (left panels) are shown in inverted grayscale (middle panels) and with colour-coded PAX polarity orientation (right panels). Yellow arrowheads indicate polarity in ≥3 consecutive cells; white arrowheads mark polarity in other cells; asterisks denote non-polar PAX distribution. Midvein (MV), lateral loops (L), higher-order veins (HV), cell conglomerates (C), and cell conglomerates at the leaf margin (M) are labeled. Scale bar, 25 µm. **(b, c)** Quantification of YFP–PAX, YFP-PAXin, YFP–D6PK and YFP-D6PKin protein polarity in the indicated genotypes. Measurements were obtained from individual cells (n = individual cells) in the first lateral loop (L1) or, in YFP–PAXin-expressing *pax d6pk012* leaves, from cell conglomerates at the leaf margin (M). At least four independent leaves were analysed per genotype. (**b**) Box plots showing individual measurements (dots), median (line), interquartile range (box), and minimum–maximum values (whiskers) of protein peak width. Outliers were removed from the graphical display using the ROUT method (Q = 0.1%). Statistical analyses were performed on the complete dataset. (c) Graphs showing individual measurements, the median, and interquartile range of protein peak orientation. Groups not sharing the same letter (**a-c**) differ significantly (adjusted *P* < 0.05). **(d)** Representative dark-field images of the marginal zone of 10-day-old leaves (left lower corner) and inverted grayscale images of the marginal zone from 5-day-old leaves stained with calcofluor white to visualise cell walls. **(e)** Z-projections of representative confocal scans of 5-day-old (D) leaf primordia from *pax d6pk012* mutants expressing D6PKp::YFP-D6PK or D6PKp::YFP-D6PKin. Magnified views of framed areas (left panels) are shown in inverted grayscale (middle panels) and with colour-coded D6PK polarity orientation (right panels). Yellow arrowheads indicate polarity in ≥3 consecutive cells; white arrowheads mark polarity in other cells; asterisks denote non-polar D6PK distribution. Midvein (MV), lateral loops (L), higher-order veins (HV), and cell conglomerates (C) are labeled. Scale bar, 25 µm. Source data

Kinase-inactive PAX partially rescued *pax* but not *pax d6pk012*, indicating that PAX function depends on D6PK/D6PKLs (Extended Data Fig. 1d–f). Active PAX showed coordinated polarity in both genetic backgrounds, whereas inactive PAX lost polarity in *pax d6pk012* and showed diffuse expression resembling auxin accumulation patterns (Extended Data Fig. 9a–d). This loss of polarity correlated with impaired cell alignment and elongation (Supplementary Fig. 4).

Kinase-inactive D6PK showed similar behaviour but retained polarity within isolated procambial clusters, resembling PIN1 polarity in *pax d6pk012* (Extended Data Fig. 9e). PAX and D6PK/D6PKLs are thus mutually required for coordinated protein polarity and tissue organization.

### PAX is required for auxin-induced procambial strand formation

**Fig. 5:**
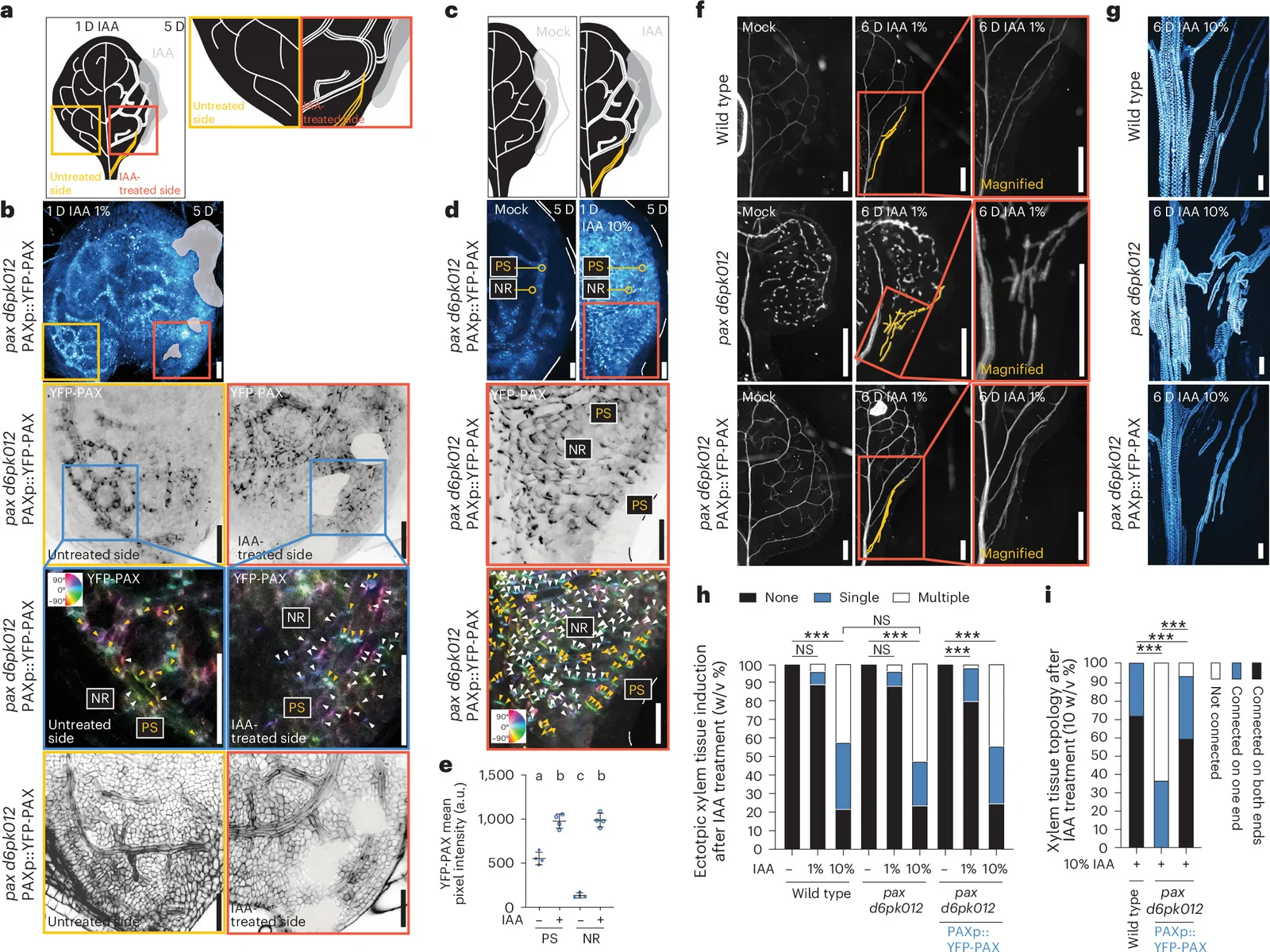
Auxin-induced ectopic procambial strand formation is PAX dependent. **a**, Schematic of a 5-day-old (D) leaf lamina and typical vein network in wild type (left) and after 24 h treatment with 1% IAA in lanolin paste (right, grey area). Yellow and red frames indicate regions analysed in **b**. **b**, *Z*-projections of confocal images showing YFP-PAX in 5-day-old (D) leaf primordia of PAXp::YFP-PAX *pax d6pk012*. Magnified views of the red and yellow framed regions (top) are shown in inverted greyscale with CFW cell wall staining, alongside schematic representations of procambial strands: newly formed (yellow lines) and pre-existing (white lines). Blue frames highlight areas with colour-coded PAX polarity orientation. Yellow arrowheads indicate coordinated polarity in three or more consecutive cells; white arrowheads mark other cells. PS, procambial strand; NR, neighbouring region. Scale bars, 50 µm. **c**, Schematic of a 5-day-old (D) leaf lamina and vein network after mock (left, white) or 24 h IAA (right, grey) treatment. Newly formed procambial strands (yellow lines) and pre-existing strands (white lines) are indicated. **d**, *Z*-projections of YFP-PAX in PAXp::YFP-PAX *pax d6pk012* leaf primordia after mock (left) or 10% IAA treatment (right). Insets show magnified red-framed regions in inverted greyscale and polarity orientation. Arrowhead notation as in **b**. Scale bars, 25 µm. **e**, Quantification of YFP–PAX mean pixel intensity in PS and NR cells of 5-day-old leaves treated with 10% IAA (+) or mock (−). Each dot represents one leaf (*n* = 4), with the value calculated as the mean pixel intensity of ten background-subtracted ROIs (technical replicates). Data are presented as mean ± s.d. Groups not sharing the same letter (**a**–**c**) differ significantly (adjusted *P* < 0.05). **f**, Representative dark-field images of cleared 10-day-old leaves treated for 6 days with 1% IAA or mock, showing the indicated genotypes. Red frames mark magnified regions in the right panels. Yellow lines denote IAA-induced xylem tissue. Scale bars, 1 mm. **g**, *Z*-projections of autofluorescent xylem cell walls in 10-day-old leaf laminae treated with 10% IAA. Scale bars, 25 µm. **h**,**i**, Stacked bar charts showing ectopic xylem induction following 1% or 10% IAA treatment or mock (−) treatment (**h**) and the topology of induced xylem tissue following 10% IAA treatment in the indicated genotypes (**i**). Classification categories are indicated in the graphs. Sample sizes: *n* = individual leaves for **h** and induced veins for **i**. ***P < 0.001. Source data

**Extended Data Fig. 10:**
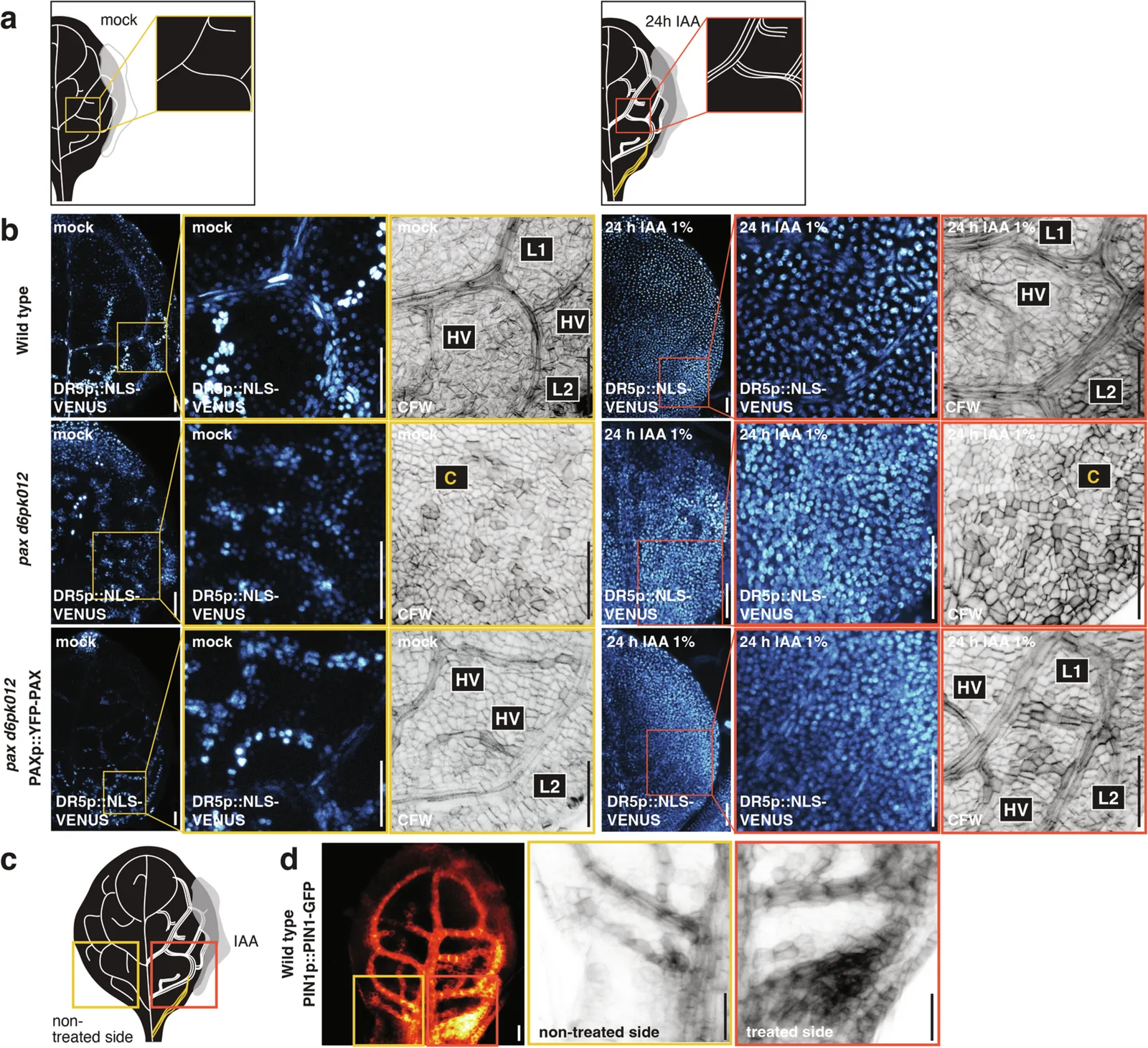
Auxin response following local auxin treatment is independent of PAX. **(a)** Schematic of a 4-day-old (D) leaf lamina and vein network in wild type (left) and after 24 h treatment with 1% IAA in lanolin paste (gray area, right). Yellow (left) and red (right) frames indicate regions enlarged in (**b**). Newly induced procambial strands post-IAA treatment are shown as yellow lines; existing procambial strands as white lines. **(b)** DR5p:NLS-VENUS expression in Z-projections of representative confocal images of 5-day-old (D) leaf primordia in wild type, *pax d6pk012*, and PAXp::YFP-PAX *pax d6pk012* after 24 h mock or 1% IAA treatment. Magnified views of yellow (mock) and red (IAA) framed areas from full images (left) are shown with DR5p:NLS-VENUS signal and in inverted grayscale with calcofluor white (CFW) cell wall staining. Cell conglomerates (C) are indicated. Scale bar, 50 µm. **(c)** Schematic of a 5-day-old (D) leaf lamina and vein network in wild type (left) and following 24 h of 1% IAA treatment (right). Lanolin paste with IAA is shown as a gray area. Yellow and red frames correspond to regions enlarged in (**d**). Newly formed procambial strands after IAA treatment are indicated by yellow lines; existing procambial strands by white lines. **(d)** PIN1-GFP localisation in Z-projections of representative confocal images of 5-day-old wild-type leaf primordia with 24 h 1% IAA treatment (yellow frame) or untreated (red frame). Magnifications of framed areas from full images (left) are shown in inverted grayscale. Scale bars, 50 µm.

Local auxin application can trigger the ectopic formation of procambial strands that connect to the existing vasculature^12,22^. In wild-type leaves, auxin treatment broadened auxin distribution and PIN1 expression and induced expanded strands. In PAXp::YFP-PAX *pax d6pk012*, auxin induced local PAX expression and strong coordinated PAX polarity along newly forming strands, which differentiated into continuous xylem (Fig. 5 and Extended Data Fig. 10). By contrast, auxin-treated *pax d6pk012* formed disorganized procambial clusters lacking connectivity (Fig. 5 and Extended Data Fig. 10). Thus, auxin induces PAX expression and polarity, and PAX is essential for organized procambial strand formation during both normal and ectopic vein development.

## Discussion

**Fig. 6:**
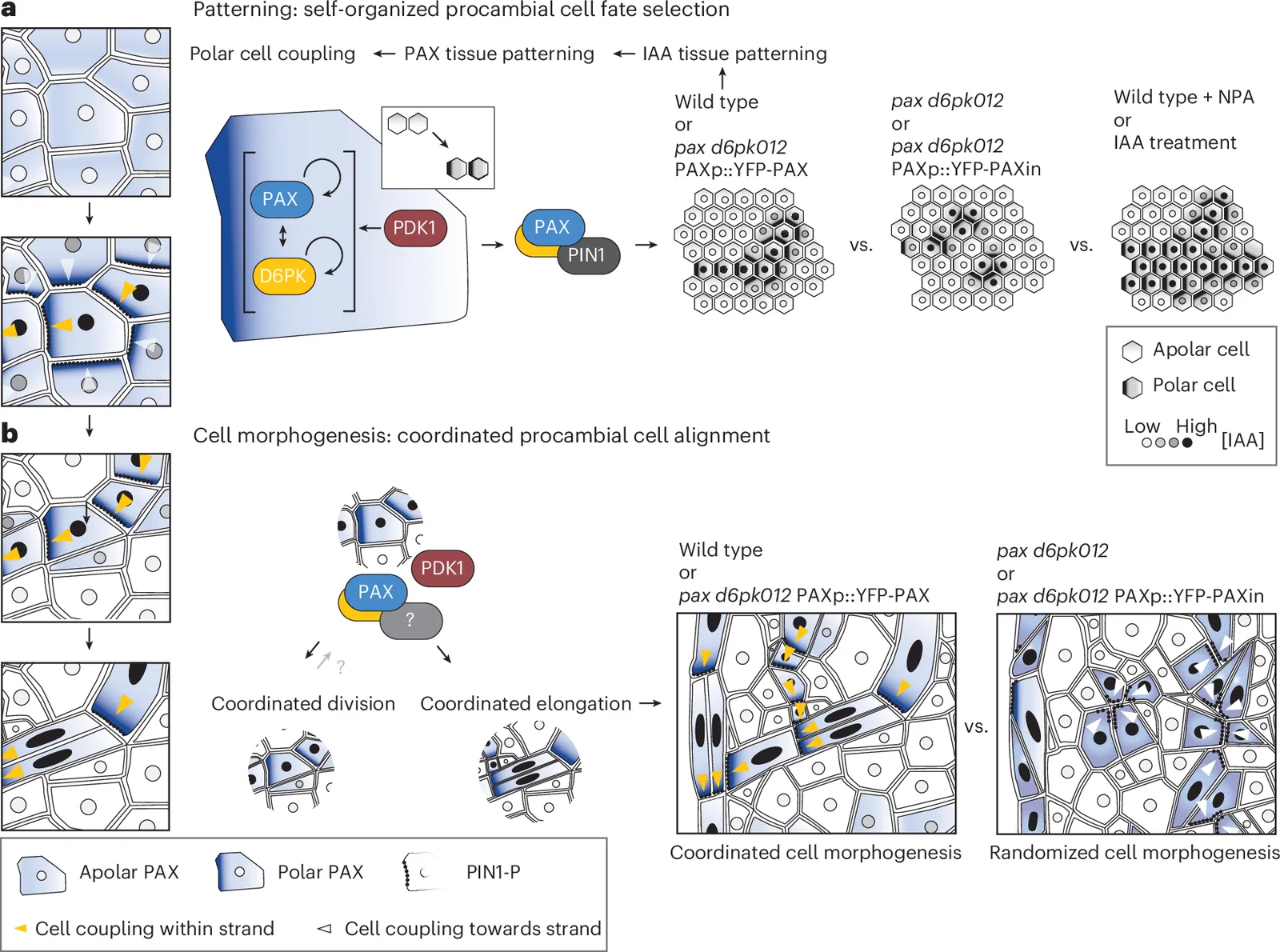
Model for the role of PAX in polar cell coupling, tissue patterning and morphogenesis during coordinated procambial cell alignment. **a**, Patterning during self-organized procambial cell fate selection. PAX catalytic activity, together with D6PK and upstream activation by PDK1, is rate limiting for the initial symmetry breaking and polarity establishment in ground-meristematic cells and the coordinated alignment of PAX, D6PK and PIN1 polarity across contiguous cells (polar cell coupling). In nearly isodiametric ground-meristem cells, the PAX polar domain consistently localizes to the interface with a single neighbouring PAX-polarizing cell, even when several adjacent cells direct their PAX domains towards the same target cell. This multicell convergence generates a funnel-shaped polarity-propagation pattern, with the narrow apex positioned adjacent to an existing PAX-polarized procambial strand and the broad end extending distally into the leaf primordium. Second, PAX catalytic activity promotes PIN1-dependent directional auxin transport, and resulting auxin flux patterns sharpen PAX expression into narrow axial domains. In PAX activity-deficient backgrounds, for example, *pax d6pk012* PAXp::YFP-PAXin, PAX domains expand and fragment into isolated clusters, parallelling disrupted auxin distribution and PIN1 expression domains. PIN-mediated auxin transport inhibition by NPA broadens the PAX expression domain and enlarges the field of cells showing coordinated PAX polarity, whereas local auxin application induces localized, polarized PAX expression. These processes require PAX kinase activity and cooperativity with D6PK/D6PKL. **b**, Cell morphogenesis and coordinated procambial cell alignment. PAX activity is essential for the coordinated procambial cell-axis alignment and end-to-end assembly of procambial cells within procambial strands through an effector and mechanism that remain to be resolved. Domain sharpening and lateral boundary refinement may be the result of division-linked inheritance of PAX polarity for coordinated cell morphogenesis. The disrupted axial alignment in *pax d6pk012*, which results in randomized cell morphogenesis, underscores the requirement of PAX catalytic activity for continuous strand formation.

We identify PAX as a central component of a plant-specific polarity coordination mechanism that links cell polarity with morphogenesis during vascular network formation (Fig. 6). PAX appears to translate auxin-derived positional information into the coordinated axialization of procambial cells, acting through local cell–cell interactions rather than through long-range auxin transport alone.

Our observations reveal two core principles underlying PAX behaviour. First, PAX polarity propagates axially between neighbouring cells, enabling a single polarized cell to orient multiple adjacent cells (Fig. 6a). Second, auxin defines *PAX* expression domains, while PAX in turn feeds back on auxin distribution through the regulation of PIN proteins (Fig. 6b). In addition, asymmetric retention of PAX during formative cell divisions may sharpen procambial strand boundaries and account for the radial polarity patterns observed when auxin transport is disrupted (Fig. 6b). Together, these observations support a dynamic model in which auxin biosynthesis, tissue-wide auxin distribution and auxin signalling regulate *PAX* transcription and activity during leaf development. These processes are probably mediated, at least in part, through PIN auxin transporters and auxin response pathways. PAX-dependent polarity could then become self-reinforcing, promoting coordinated alignment of polarized cells that ultimately gives rise to continuous procambial strands.

Although our data establish PAX as an important coordinator of vascular polarity, the molecular mechanisms linking auxin perception to PAX function and intercellular polarity propagation remain unresolved. Several previously identified regulators of vascular development may act within this framework. For example, mutants of the auxin response factor *MP* (*MONOPTEROS*) fail to establish a functional leaf vascular system, suggesting that MP may contribute to auxin-responsive regulation of *PAX* expression^15,23^.

Among characterized vein-patterning mutants, *sfc/van3* and the *cvp2 cvl* double mutant most closely phenocopy the severe vein fragmentation observed in *pax d6pk012* mutants^24,25^. SFC/VAN3 (SCARFACE/VASCULAR NETWORK DEFECTIVE3) encodes an ADP-ribosylation factor GTPase-activating protein that may facilitate PAX delivery and/or recycling at the plasma membrane^24,26,27^. Similarly, the inositol polyphosphate 5′-phosphatases CVP2 (COTYLEDON VASCULAR PATTERNING2) and CVL (CVP2-LIKE) regulate membrane phosphoinositide composition and could thereby influence PAX stability or retention at the plasma membrane^25^. Such mechanisms are consistent with previous studies demonstrating phosphoinositide-dependent regulation of PAX and other AGC1 kinase family members^10,28,29^. The guanine nucleotide exchange factor GNOM may further contribute to these processes by coordinating trafficking of both PIN auxin transporters and PAX/D6PK family kinases^30,31^.

Sterol composition may represent an additional layer of polarity regulation during vascular development. Mutants in the three Arabidopsis *SMT* (*C-24 STEROL METHYLTRANSFERASE*) genes exhibit vascular defects associated with impaired cellular axial organization^32^. In particular, *cvp1/smt2* and *smt3* are required for the synthesis of downstream C24-ethyl sterols such as 24-ethylidenelophenol^32^. Loss of *SMT2* function in the *cvp1* (*cotyledon vascular patterning 1*) background is further enhanced by *smt3* mutations, and the resulting vascular defects have been associated with impaired cellular axial organization^32^. These findings suggest that sterol composition contributes to the establishment or maintenance of cell polarity during vascular development, potentially by modulating membrane properties required for coordinated PAX polarity establishment during procambial strand formation.

Our findings also raise the possibility that additional signalling pathways modulate PAX-dependent polarity coordination. BRX family proteins, which interact with PAX during protophloem development in the root, may similarly function during leaf procambial strand formation^7^. Notably, some BRX family proteins act as auxin-sensitive negative regulators of both PAX activity and auxin transport, suggesting that they may provide feedback control within the auxin–PAX regulatory network^7,33,34^. Peptide-mediated signalling pathways, including CLE (CLAVATA3/EMBRYO SURROUNDING REGION-related) and TDIF (TRACHEARY ELEMENT DIFFERENTIATION INHIBITORY FACTOR) signalling, may likewise participate in the process^29,35–38^.

Beyond cell-autonomous mechanisms, PAX may also operate within a plasmodesmata-mediated signalling network. Plasmodesmata aperture is regulated by callose deposition, which controls pore permeability, and both narrowing and widening of plasmodesmata can produce vascular defects including open vein loops, vein fragments and vascular clusters^39^. The similarity of these phenotypes to those observed in *pax d6pk012* mutants raises the possibility that plasmodesmata-mediated intercellular flux contributes to PAX-dependent coordination of cell and tissue polarity.

Genetic analyses of *PAX* and *D6PK* further reveal a context-dependent functional interdependence in which kinase activity becomes limiting only in the absence of the cooperating kinase. Notably, PAX and D6PK exhibit distinct spatial deployment patterns during vascular development. Whereas *PAX* expression domains are progressively refined into narrow procambial cell files, *D6PK* expression remains broadly distributed throughout the ground meristematic tissue. Consistent with these differences, expansion of the *PAX* expression domain under the *D6PK* promoter strongly rewires the density and spatial distribution of PAX polarity domains, whereas D6PK polarity remains comparatively restricted to incipient procambial strands. These observations demonstrate that spatial deployment of distinct AGC1 kinase activities can actively shape tissue-scale polarity organization. Complementation analyses further indicate that, although both kinases contribute to polarity coordination, PAX more effectively supports long-range polarity propagation and vascular connectivity. The molecular basis underlying this functional specialization remains unresolved and represents an important direction for future investigation.

## Methods

### Biological material

All experiments were performed in *A. thaliana* Columbia-0 (Col-0) background. The *PAX* (AT2G44830) loss-of-function allele *pax-1* (*pax*; SAIL_688_B04) was previously described and used as a reference *pax* allele for all studies^7^. *pax-3* (SAIL_713_A02) was obtained from the Nottingham Arabidopsis Stock Center (NASC) and genotyped for homozygosity. Single (*d6pk0, d6pkl1, d6pkl2*), double (*d6pk01*) and triple (*d6pk012*) mutants of *D6PK* (AT5G55910), *D6PKL1* (AT4G26610) and *D6PKL2* (AT5G47750) were previously described^6^. *pax d6pk d6pkl1* (*pax d6pk01*) and *pax d6pk d6pkl1 d6pkl2* (*pax d6pk012*) were obtained by crossing *pax-1* and *d6pk012*, which combines the alleles *d6pk-1* (SALK_061847), *d6pkl1-1 (SALK_056618)* and *d6pkl2-2* (SALK_086127)^6^.

PAXp::YFP-PAX and PAXp::YFP-PAXin, carrying a K392E mutation in the ATP-binding site, were obtained by floral dip transformation of the respective transgene into *pax*^40^. PAXp::YFP-PAX *pax* and PAXp::YFP-PAXin *pax* were subsequently crossed into *d6pk012* to obtain PAXp::YFP-PAX *pax d6pk012* and PAXp::YFP-PAXin *pax d6pk012*. As homozygous *pax d6pk012* did not produce progeny, homozygous *pax d6pk012* mutants were repeatedly isolated from plants segregating for PAXp::YFP-PAX in *pax d6pk012* by PCR-based genotyping from a segregating parent line as described^6,7^.

PIN1p::PIN1-GFP and DR5p::NLS-VENUS were gifts from Remko Offringa (Leiden University, Netherlands) and Rishikesh P. Bhalerao (Umea Plant Science Center, Umea, Sweden), respectively.

The *pdk1-14 pdk2-4 (pdk1 pdk2)* double mutant, PDK1p::YFP-PDK1 *pdk1 pdk2*, PDK1p::YFP-PAX *pdk1 pdk2* and PDK1p::YFP-PAX S596D *pdk1 pdk2* were previously published and provided by Remko Offringa (Leiden University, Netherlands) and genotyped for the *pdk2* allele as previously described when isolated from a segregating population^19^. PDK1p::YFP-D6PK *pdk1 pdk2* and PDK1p::YFP-D6PK S345D *pdk1 pdk2* were previously described^21^.

### Molecular cloning

To generate the PAXp::YFP-PAX construct, a 2-kb promoter fragment upstream of the *PAX* (AT2G44830) start codon and the *PAX* genomic coding region were amplified from *A. thaliana* Col-0 genomic DNA using primer pairs 1 and 2, and 3 and 4, respectively. Both fragments were cloned into the pDONR201 vector (Invitrogen). The *PAX* genomic fragment was then recombined into the destination vector pEXTAG-YFP-GW (a gift from Jane Parker, Cologne, Germany) to generate 35Sp::YFP-PAX. Next, the PAX promoter fragment from pDONR201 and the YFP-PAX fusion transcript from pEXTAG-YFP-PAX were excized using KpnI/XhoI and XhoI/NotI, respectively, and ligated into the binary vector pGREEN0229. The resulting PAXp::YFP-PAX construct was amplified with primers 1 and 4, cloned again into pDONR201 and transferred into the binary vector pFAST R07, from which the original EGFP gene had been removed by PCR-based deletion mutagenesis using primers 5 and 6. Kinase-inactive variants PAX K392E and D6PK K138E were generated via site-directed mutagenesis using phosphorylated primers 7 and 8, respectively.

Primer sequences: primer 1: GGGGACAAGTTTGTACAAAAAAGCAGGCTTCGG TTACCGCTCGGAGAATTTTCAACTTCATTATAGTTAGTGAAG; primer 2: GGGGACCACTTT GTACAAGAAAGCTGGGTCCTCGAGTCATGCGATTTTTAACACCAAACACAAATCTCTC; primer 3: GGGGACAAGTTTGTACAAA AAAG CAGGCTTAATGCTGCAAATGGAAAGAGTTG; primer 4: GGGGACCACTTTGTACAAGAAAGCTGGGTATTAGAAAAACTCAAAGTCTAG AAATTTACC; primer 5: GGTGATATCCCGCGGCCCGGCATGTTACGTCC; primer 6: ACGTAACATGCCGGGCCGCGGGATATCACCACTTT; primer 7 (phosphorylated): GCTTTA TCCATGACTTCCACAGCAAAATGG; primer 8 (phosphorylated): TTATTTTGCGATGGAGGTTA TGG.

### Plant growth conditions

For phenotypic analysis, seeds were surface-sterilized, stratified for 2 days at 4 °C and then germinated on half-strength Murashige and Skoog medium (1/2 MS) supplemented with 0.3% sucrose at 21 °C under continuous light (110–150 μmol m^−2^ s^−1^) in MLR-351 SANYO growth chambers (Ewald)^7^. Adult plants were grown under continuous white light (110–130 μmol m^−2^ s^−1^) in MobyLux GroBanks (CLF Plant Climatics).

### Staging of leaf development

Sterilized stratified seeds germinated for 4, 4.5, 5 and 6 days in the light produced first leaves that are approximately 2, 2.5, 3 and 4 days old, respectively. This staging enables the analysis of vein formation events, with loop 1 (L1) typically initiating around 2–2.5 days, loop 2 (L2) around 3 days and HVs from approximately 4 days onwards^39^.

### Analysis of vein network topology

The vein network parameters connectivity, continuity and cardinality were calculated as previously described^11^: The connectivity index quantifies the proportion of vein segments with both ends connected to other veins, reflecting the extent of interconnection in the vascular network, including the formation of loops and HVs. The continuity index measures the proportion of vein fragments with at least one end in contact with another vein. A low continuity index indicates a high frequency of unconnected fragments. The cardinality index represents the absolute number of veins with at least one end connected to the residual vascular network. These parameters were determined from images of the first leaves of 12-day-old light-grown plants. Leaves were fixed overnight at 4 °C in methanol:acetic acid (3:1), then cleared overnight at 4 °C in 100% ethanol. Dehydration was performed through an ethanol series (80%, 60%, 40%, 20% and 0% (v/v)) over the course of 1 day. Cleared leaves were mounted in 80% (w/v) chloral hydrate and imaged using an Olympus SZX7 stereomicroscope (Olympus Corporation).

Statistical analyses were conducted using GraphPad Prism version 9.0.

### Fixed ClearSee sample preparation for confocal microscopy

All confocal images were acquired from samples prepared using the ClearSeealpha protocol^41^. Seedlings at defined developmental stages were fixed in 4% paraformaldehyde (PFA) dissolved in 1× PBS (pH 7.4) for 1 h under vacuum at room temperature. Following fixation, samples were rinsed twice with 1× PBS (pH 7.4) and cleared in ClearSeealpha solution supplemented with 50 mM Na_2_SO_4_ for 4 days. To ensure efficient clearing, the ClearSeealpha solution was refreshed every 24 h.

For cell wall staining, the clearing solution was replaced on day 3 with ClearSeealpha supplemented with 50 mM Na_2_SO_4_ and 0.1% (v/v) calcofluor white (CFW), and samples were stained overnight. Stained seedlings were then rinsed in ClearSeealpha solution containing 50 mM Na_2_SO_4_ but without CFW for 1–2 h before mounting.

Cleared and stained seedlings were mounted in antifade mounting medium (90% glycerol, 2.5% DABCO, 1× PBS, pH 8.8) and stored at –20 °C until imaging.

### Sample preparation for dark-field illumination microscopy

For the visualization of vein networks, 10-day-old leaves were fixed overnight at 4 °C in methanol:acetic acid (3:1) and then cleared for 1 day at 4 °C in 100% ethanol. Samples were subsequently dehydrated stepwise through an ethanol series (80%, 60%, 40%, 20% and 0% (v/v)) over the course of 1 day. Cleared leaves were mounted in 80% (w/v) chloral hydrate and stored at 4 °C until imaging. Dark-field illumination microscopy for xylem topology imaging was performed using an Olympus SZX7 stereomicroscope (Olympus Corporation).

### Confocal microscopy

Fluorescence confocal imaging was conducted on either an Olympus FV1000 (Olympus Corporation) or a Leica SP8 confocal microscope (Leica Microsystems).

### Image analysis

Cell and tissue morphology parameters were assessed from CFW-stained images. Cell outlines were manually traced to determine cell circumferences. For cell alignment in angle measurements, regions of interest (ROIs) were defined along incipient veins using the Segmented Line Tool in Fiji, followed by image straightening using the Straighten Tool^42^. Maximal two-dimensional cell length, cell orientation relative to the vein and aspect ratio were quantified using the Feret, Feret Angle and Aspect Ratio (AR) tools in Fiji, respectively. Statistical analyses were performed in GraphPad Prism (v9.0). Protein distribution and polarity were quantified from processed images. A segmented line was drawn along the region of polar protein accumulation, defining a 180° analysis window. Fluorescence intensity profiles were extracted using the Profile Plot Tool, and Gaussian fitting was applied to measure peak amplitude and peak width as indicators of polarity. The position of the fluorescence peak along the 180° window was used to determine polarity orientation relative to the vein. In addition, cell polarity indices were calculated from greyscale images using the OrientationJ plugin in Fiji^42^. Lookup tables for all confocal images were adjusted using Fiji to ensure consistent image presentation.

### Auxin and inhibitor treatments

Auxin treatments were performed by applying solidified lanolin paste containing either 1% or 10% (w/v) indole-3-acetic acid (IAA) or IAA-free lanolin for mock controls. The paste was applied to one leaf margin of 4-day-old first leaves using minutien pins as described previously^22,43^. Before application, lanolin was preheated to 60 °C, mixed with 1% (w/v) IAA and allowed to cool to room temperature before use.

Auxin transport inhibitor treatments were performed by germinating seedlings on 1/2 MS medium containing 0.3% sucrose, supplemented with 25 µM *N*-1-naphthylphthalamic acid (NPA) dissolved in dimethyl sulfoxide (DMSO). Mock treatments consisted of DMSO-only controls.

### Antibody generation and validation

The anti-PIN1 S1-P and anti-PIN1 T1-P S1-P antibodies were custom-generated as phospho-specific rabbit polyclonal antibodies raised against the synthetic peptides CGLSATPRPS(PO_3_H_2_)NLT and CGLSAT(PO_3_H_2_)PRPS(PO_3_H_2_)NLT, respectively (Eurogentec). Antibody specificity was validated by dot-blot analysis as previously described using non-phosphorylated, singly phosphorylated and doubly phosphorylated peptide variants^16^. All other PIN1 phosphosite-specific antibodies have been previously described and validated^16^.

### Whole-mount immunolocalization

Whole-mount immunolocalization on first leaf primordia was performed as previously described, with minor modifications^16^. Seedlings were fixed in 1 ml 100% methanol for 20 min at 37 °C, followed by 3 min incubation in 0.8 ml preheated 100% methanol (60 °C). Rehydration was carried out stepwise by adding 0.1 ml water every 2 min until a final concentration of 20% methanol was reached. Samples were then rinsed twice in 1 ml 1× PBS (pH 7.4) before proceeding with the immunolocalization protocol as previously described^16^. Primary antibodies were used at the following dilutions: 1:1,000 for anti-PIN1 rabbit (Abiocode); 1:100 for anti-PIN1 S4-P rabbit^16^; 1:250 for anti-PIN1 S1-P rabbit; and 1:500 for anti-PIN1 S1-P T1-P rabbit. The secondary antibody used was Cy3-conjugated antirabbit IgG (Dianova) at a dilution of 1:600. Samples were mounted in antifade mounting medium (90% glycerol, 2.5% DABCO, 1× PBS, pH 8.8) and stored at −20 °C until imaging.

### Statistics and reproducibility

All microscopy images shown are representative of observations reproduced in at least 2–4 independent experiments using multiple biological replicates. Reported genotype-dependent cellular phenotypes and protein localization patterns were consistently reproduced across developmental stages, pharmacological treatments and immunostaining experiments analysed in this study. For quantitative analyses, the unit of analysis, sample size (*n*), statistical test and multiple-comparisons procedure are reported in the corresponding figure legends. *n* denotes independent biological replicates, corresponding to either individual leaves or individual cells, depending on the biological question addressed, as indicated in the respective figure legends. Where measurements from tissue morphology or tissue ROIs were averaged for a biological replicate, these represent technical replicates and were not treated as independent observations.

### Reporting summary

Further information on research design is available in the Nature Portfolio Reporting Summary linked to this article.

## Supplementary information

Supplementary Information Supplementary Figs. 1–4, statistical information for Figs. 1–6 and statistical information for Extended Data Figs. 1–10.

Reporting Summary

Peer Review File

## Source data

Source Data Figs. 2, 3 and 5 and Extended Data Figs. 1, 4, 7 and 9 Source data of all quantifications shown in Figs. 2, 3 and 5 and Extended Data Figs. 1, 4, 7 and 9.

## Data Availability

All data and materials used in the analysis will be made available on requests addressed to C.S. (claus.schwechheimer@tum.de). Source data are provided with this paper.
