# Publisher Correction: PAX-regulated self-organizing polarity drives procambial strand formation

**DOI:** 10.1038/s41477-026-02424-3

**Published:** 2026-09-14

**Authors:** Alkistis E. Lanassa Bassukas, Yao Xiao, Namuk Kim, Claus Schwechheimer

**Affiliations:** 1 Plant Systems Biology, School of Life Sciences, Technical University of Munich, Freising, Germany; 2 Present Address: Guangdong Provincial Key Laboratory of Plant Adaptation and Molecular Design, School of Life Sciences, Guangzhou University, Guangzhou, China; 3 Present Address: Korea Research Institute of Bioscience and Biotechnology, Yuseong-gu, Republic of Korea

**Keywords:** Leaf development, Patterning

Correction to: *Nature Plants* https://doi.org/10.1038/s41477-026-02382-w, published online 27 August 2026.

In the version of this article initially published, there were graphical display errors in Figs. 1a, 2b, c, e, 3a–d, f, g, 4a–d, f, 5f, Extended Data Figs. 2, 7 which are now amended in the HTML and PDF versions of the article.

