# Supplementary material for: PAX-regulated self-organizing polarity drives procambial strand formation
Source: Nat Plants. 2026 Aug 27;12(10):2108–19. doi: 10.1038/s41477-026-02382-w (PMC13680282; doi:10.1038/s41477-026-02382-w)
Supplement: Supplementary file 1 [file 41477_2026_2382_MOESM1_ESM.pdf]

nature plants

Supplementary information https://doi.org/10.1038/s41477-026-02382-w

PAX-regulated self-organizing polarity drives procambial strand formation

In the format provided by the authors and unedited

SUPPLEMENTARY FIGURES

Supplementary Figure S1

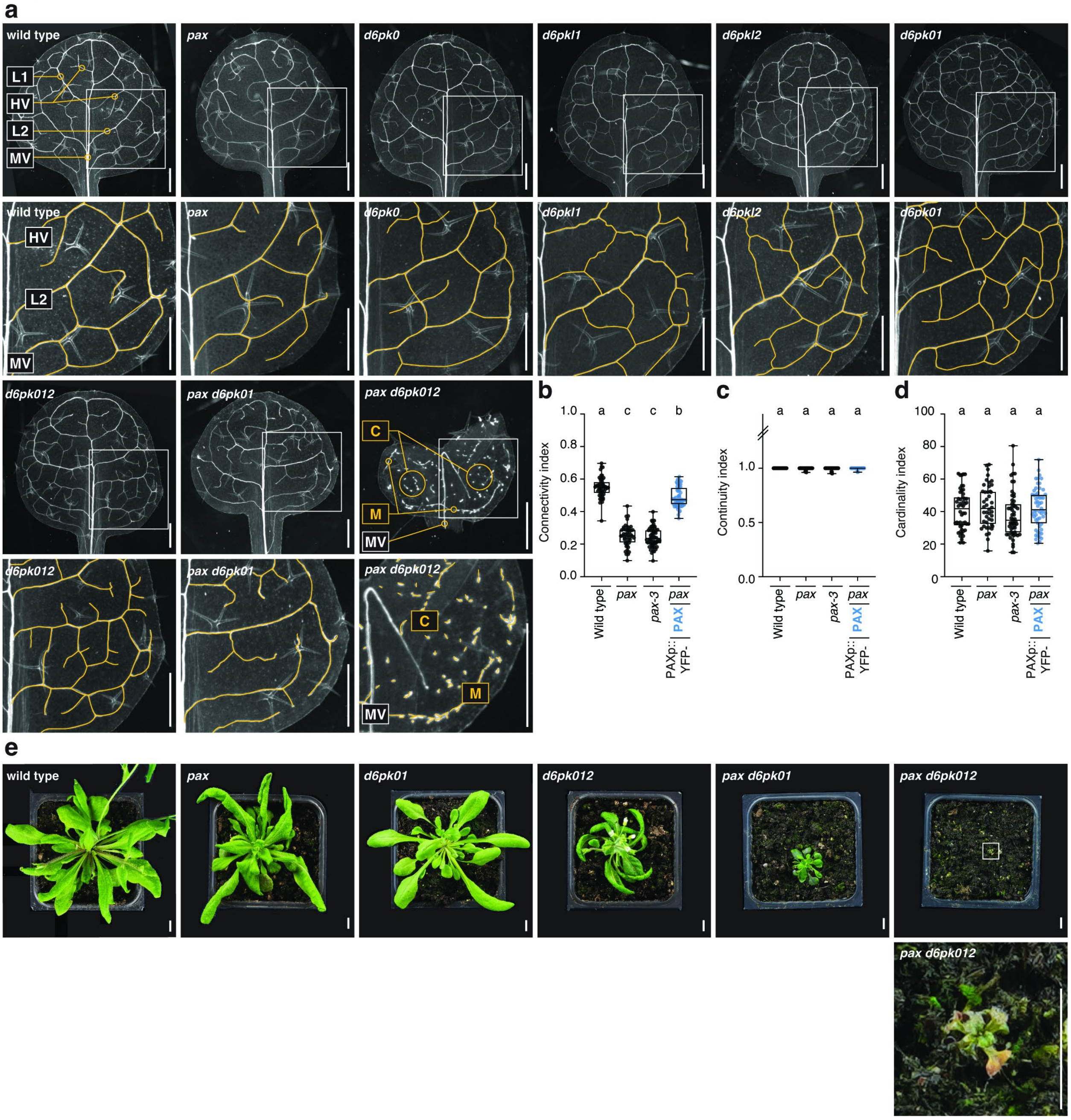

Supplementary Figure S1 | Mutations in *PAX* and individual *D6PK/D6PKLs* cooperatively define vascular network topology in *Arabidopsis thaliana* leaves. (a) Representative dark-field microscopy images showing xylem networks in cleared leaves from 10-day-old plants of indicated genotypes. White frames in upper panels indicate magnified regions shown below. The midvein (MV), selected lateral loops (L), higher-order veins (HV), cell conglomerates in the leaf lamina (C), and margin (M) are labeled; all veins except the MV are highlighted in yellow. The *pax d6pk012* leaf is partially folded due to the rigid structure of the leaf; bright, scattered structures are trichomes. All images originate from the same experimental series; images for wild type, *pax*, *d6pk012*, and *pax d6pk012* are identical to those in Figure 1a. Scale bars, 1.5 mm. **(b–d)** Box plots depicting individual measurements (dots), median (line), interquartile range (box), and range, and minimum–maximum values

(whiskers) for vein connectivity (b), continuity (c), and cardinality (d) indices in leaves with two independent *pax* alleles and a PAXp::YFP-PAX complementation line. Sample sizes (*n* = individual leaves) and statistical analyses are provided as Supporting Data in the Statistical Data and Source Data files. Groups not sharing the same letter (a–c) differ significantly (adjusted $P < 0.05$). **(e)** Representative photographs of 1-month-old plants of indicated genotypes. Scale bars, 8 mm.

Supplementary Figure S2

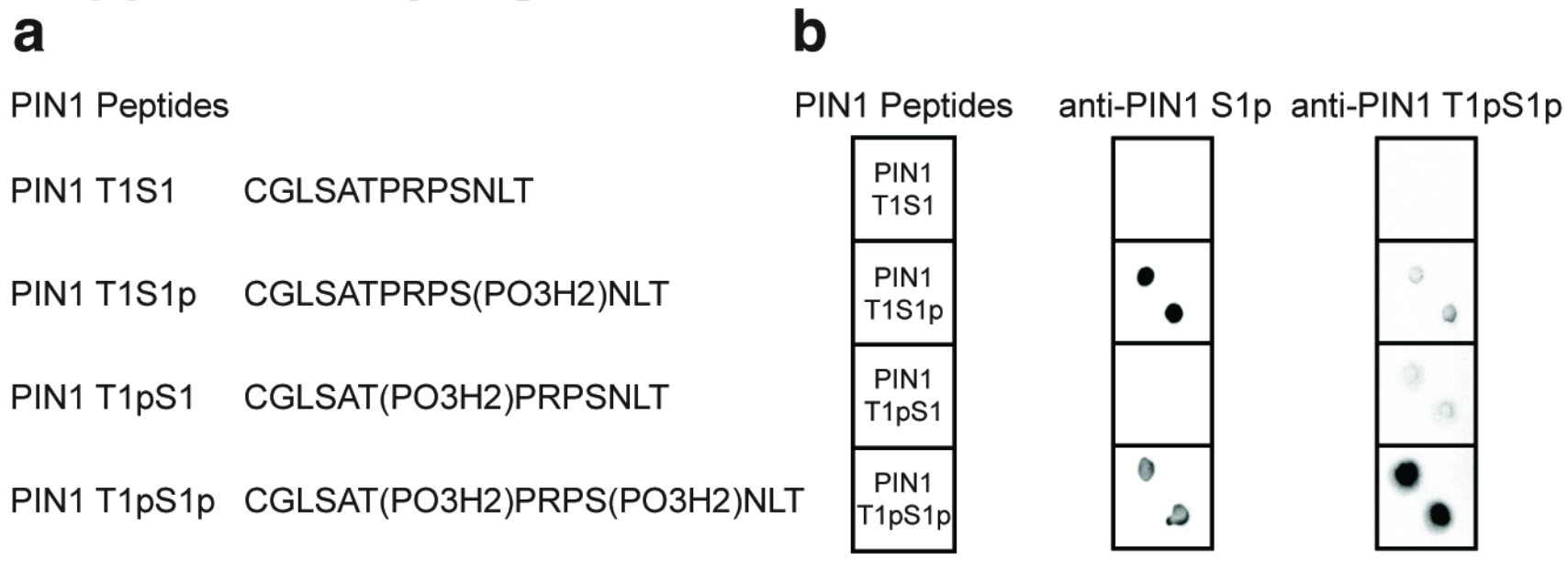

Supplementary Figure S2 | Specificity of phosphosite-specific anti-PIN1 antibodies. (a) Sequences of synthetic PIN1 peptides used for antibody validation, shown in their unmodified and phosphorylated (PO_3H_2) forms, with single or double phosphorylation at the S1 and T1 sites of PIN1, as previously described [9]. **(b)** Dot blot analysis of the peptides shown in (a), spotted in duplicate and probed with anti-PIN1 S1p and anti-PIN1 S1pT1p antibodies. The results confirm antibody specificity for the corresponding phosphorylated PIN1 epitopes. All other additional phosphosite-specific antibodies used in this study were previously described [16].

Supplementary Figure S3

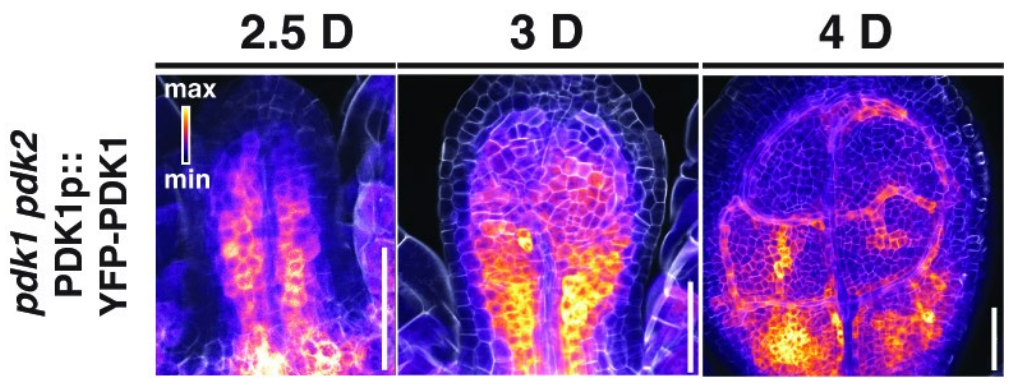

Supplementary Figure S3 | PDK1 expression during leaf development. Z-projections of representative confocal images of 2.5-, 3-, and 4-day-old (D) *pdk1 pdk2* mutant leaf primordia expressing PDK1p::YFP-PDK1. A calibration bar indicating minimum and maximum signal intensity is shown. Scale bars, 25 µm.

Supplementary Figure S4

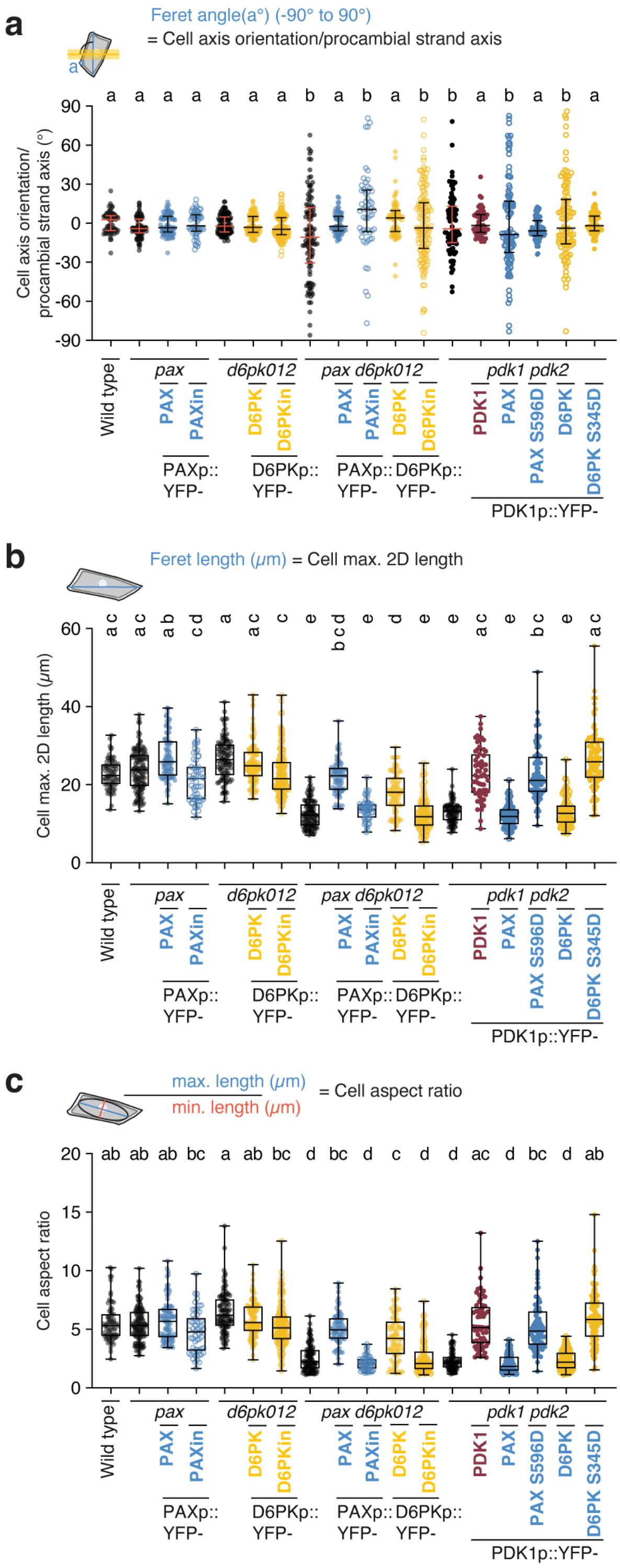

Supplementary Figure S4 | PAX and D6PK/D6PKL kinases function interdependently to coordinate cell polarity downstream of PDK1. (a-c) Quantification of cell morphology in the indicated genotypes. Measurements were obtained from individual cells (n = individual cells) in the first lateral loop (L1) or, in *pax d6pk012*, *pdk1 pdk2*, and their respective non-complementing lines, from cell conglomerates at the leaf margin (M). At least four independent leaves were analysed per genotype. (a) Graph showing individual measurements (dots),

median, and interquartile range of cell axis orientation relative to the procambial strand axis (Feret angle). (b, c) Box plots showing individual measurements (dots), median (line), interquartile range (box), and minimum–maximum values (whiskers) of cell maximal length (Feret length; b) and cell aspect ratio (c). Sample sizes (n = individual cells sampled from at least four independent leaves per genotype) and statistical analyses are provided as Supporting Data in the Statistical Data and Source Data files. Groups not sharing the same letter (a–d) differ significantly (adjusted $P < 0.05$).

STATISTICAL INFORMATION for Main Figures

The data underlying the statistical analyses, together with the results of all pairwise multiple-comparison tests are provided as Supporting Data in the Source Data file.

Figure 2 | PAX polarity during pre-procambial cell fate selection is coordinated and follows distinct rules. (e) Protein peak width was compared using the Kruskal–Wallis test (H(8) = 142.2, $P < 0.001$) followed by Dunn's multiple-comparisons test (significant adjusted P values: 2.5 D D6PKp::YFP-D6PK vs. 3 D PIN1p::PIN1-GFP, *, $P = 0.047$; 2.5 D D6PKp::YFP-D6PK vs. 4 D PIN1p::PIN1-GFP, **, $P = 0.004$; 2.5 D PIN1p::PIN1-GFP vs. 3 D PAXp::YFP-PAX, **, $P = 0.001$; 2.5 D PIN1p::PIN1-GFP vs. 3 D D6PKp::YFP-D6PK, **, $P = 0.005$;all other comparisons, ***, $P < 0.001$). Protein peak orientation was quantified as the cell-level absolute deviation from 0° alignment relative to the procambial strand axis and analysed separately at 2.5 D, 3 D, and 4 D using Kruskal–Wallis tests (2.5 D: H(2) = 16.3; 3 D: H(2) = 20.05; 4 D: H(2) = 32.08; all $P < 0.001$), followed by Dunn's multiple-comparisons test (adjusted P values). Significant adjusted P values for : 2.5 D (D6PKp::YFP-D6PK vs. PIN1p::PIN1-GFP, **, $P = 0.001$; PAXp::YFP-PAX vs. PIN1p::PIN1-GFP, **, $P = 0.002$); 3 D, (PIN1p::PIN1-GFP vs. PAXp::YFP-PAX, **, $P = 0.002$); all other significant comparisons, ***, $P < 0.001$). Individual cells constituted the statistical unit and were sampled from the loop-forming region of the first lateral loop (L1) at developmental stages 2.5–4 D from at least four independent leaves per genotype and developmental stage. Sample sizes (n = individual cells): PAXp::YFP-PAX: 2.5 D, n = 64; 3 D, n = 64; 4 D, n = 53. D6PKp::YFP-D6PK: 2.5 D, n = 37; 3 D, n = 43; 4 D, n = 42. PIN1p::PIN1-GFP: 2.5 D, n = 51; 3 D, n = 51; 4 D, n = 62 (protein peak width) and n = 61 (protein peak orientation). Cells were sampled from at least four independent leaves per genotype and developmental stage.

Figure 3 | Cell polarisation during procambial strand formation depends on PAX and is independent of PIN-mediated auxin transport. Cell axis alignment in (e) was quantified for individual cells as absolute deviations from 0°, and group differences were assessed using the Kruskal–Wallis test ($H(8) = 227.5$, $P < 0.001$), followed by Dunn's multiple-comparisons test (significant adjusted P values: 2.5 D wild type vs. 3 D wild type, *, $P = 0.011$; 2.5 D *pax d6pk012* vs. 3 D wild type, *, $P = 0.016$; 3 D wild type vs. 4 D *pax d6pk012*, **, $P = 0.005$; all other comparisons, ***, $P < 0.001$). Group differences in maximum cell length (f) and cell aspect ratio (g) were assessed using the Kruskal–Wallis test followed by Dunn's multiple-comparisons test (adjusted P values): (f) $H(8) = 572.3$, $P < 0.001$ (significant adjusted P values: 2.5 D *pax d6pk012* vs. 3 D *pax d6pk012*, *, $P = 0.013$; all other comparisons, ***, $P < 0.001$). (g) $H(8) = 575.5$, $P < 0.001$ (significant adjusted P values: 2.5 D *pax d6pk012* vs. 3 D *pax d6pk012*, *, $P = 0.027$; all other comparisons, ***, $P < 0.001$). Sample sizes (n = individual cells): 2.5 D: WT (n=105), *pax d6pk012* (n=91), PAXp::YFP-PAX *pax d6pk012* (n=57); 3 D: WT (n=123), *pax d6pk012* (n=203), PAXp::YFP-PAX *pax d6pk012* (n=225); 4 D: WT (n=70), *pax d6pk012* (n=169), PAXp::YFP-PAX *pax d6pk012* (n=67). Cells were measured from at least 6 leaves per genotype. Groups with significant differences are indicated by letters a–e.

Figure 4 | PDKs enhance PAX and D6PK cellular polarity during procambial strand formation. (b-d) Group differences in vein connectivity (b) were assessed using one-way ANOVA followed by Tukey's multiple-comparisons test (adjusted P values) $F(6,245) = 187.1$, $P < 0.001$(significant adjusted P values: *pdk1 pdk2* vs. PDK1p::YFP-D6PK, *, $P = 0.040$; all other comparisons, ***, $P < 0.001$). Group differences in continuity (c) and cardinality (d) were assessed using the Kruskal–Wallis test ((c) $H(6) = 233.8$, $P < 0.001$; (d) $H(6) = 188.4$, $P < 0.001$) followed by Dunn's multiple-comparisons test (significant adjusted P values: (c)

PDK1p::YFP-PAX vs. PDK1p::YFP-PAX S596D, **, $P = 0.006$; all other comparisons, ***, $P < 0.001$; (d) all comparisons, ***, $P < 0.001$). Sample sizes (n = individual leaves): wild type, $n = 48$; *pdk1 pdk2*, $n = 36$; *PDK1p::YFP-PDK1 pdk1 pdk2*, $n = 53$; *PDK1p::YFP-PAX pdk1 pdk2*, $n = 17$; *PDK1p::YFP-PAX S596D pdk1 pdk2*, $n = 45$; *PDK1p::YFP-D6PK pdk1 pdk2*, $n = 11$; *PDK1p::YFP-D6PK S345D pdk1 pdk2*, $n = 42$. **(g)** Group differences in protein peak width were assessed using two-sided Mann–Whitney tests: *PDK1p::YFP-PAX* versus *PAX S596D* ($U = 1285$, $P < 0.001$); *PDK1p::YFP-D6PK* versus *D6PK S345D* ($U = 2367$, $P = 0.539$). **(h)** Protein peak orientation was quantified for individual cells as absolute deviations from 0° alignment, and group differences were assessed using two-sided Mann–Whitney tests: *PDK1p::YFP-PAX* versus *PAX S596D* ($U = 817$, $P < 0.001$); *PDK1p::YFP-D6PK* versus *D6PK S345D* ($U = 1279$, $P < 0.001$). Sample sizes (n = individual cells): (g) *PDK1p::YFP-PAX*, $n = 84$; *PAX S596D*, $n = 47$; *PDK1p::YFP-D6PK*, $n = 73$; *D6PK S345D*, $n = 69$. (h) *PDK1p::YFP-PAX*, $n = 81$; *PAX S596D*, $n = 47$; *PDK1p::YFP-D6PK*, $n = 73$; *D6PK S345D*, $n = 70$. Cells were sampled from at least four independent leaves per genotype.

Figure 5 | Auxin-induced ectopic procambial strand formation is PAX-dependent. (e) Quantification of YFP-PAX mean pixel intensity in PS and NR cells of 5-day-old leaves treated with 10% IAA (+) or mock (-). Group differences were assessed using one-way ANOVA ($F(3,12) = 142.4$, $P < 0.001$; all significant pairwise comparisons, $P < 0.001$), followed by Tukey's multiple-comparisons test (adjusted P values). Each dot represents one leaf ($n = 4$), calculated as the mean pixel intensity of 10 regions of interest (ROIs; technical replicates) after background subtraction. **(h)** differences between genotypes in the frequency of xylem induction (induction versus no induction) were assessed using two-sided Fisher's exact tests. Within genotypes: wild type, mock vs. 1% IAA ($P = 0.061$), mock vs. 10% IAA ($P < 0.001$); *pax d6pk012*, mock vs. 1% IAA ($P = 0.268$), mock vs. 10% IAA ($P < 0.001$); PAXp::YFP-PAX *pax d6pk012*, mock vs. 1% IAA ($P < 0.001$), mock vs. 10% IAA ($P < 0.001$). Between genotypes following 10% IAA treatment: wild type vs. *pax d6pk012* ($P > 0.999$); wild type vs. PAXp::YFP-PAX *pax d6pk012* ($P = 0.613$). Sample sizes (n = individual leaves): wild type, mock (n = 35), 1% IAA (n = 90), 10% IAA (n = 120); *pax d6pk012*, mock (n = 16), 1% IAA (n = 25), 10% (n = 17); PAXp::YFP-PAX *pax d6pk012*, mock (n = 45), 1% IAA (n = 94), 10% (n = 81). **(i)** Group differences were assessed using two-sided Fisher's exact tests on the frequency of connected (connected at one or both ends) versus non-connected induced veins following 10% IAA treatment: wild type vs. *pax d6pk012* ($P < 0.001$); wild type vs. PAXp::YFP-PAX *pax d6pk012* ($P < 0.001$); *pax d6pk012* vs. *PAXp::YFP-PAX pax d6pk012* ($P < 0.001$). Sample sizes (n = induced veins): wild type 10% IAA (n = 146); *pax d6pk012* 10% IAA (n = 22); PAXp::YFP-PAX *pax d6pk012* 10% IAA (n = 101) within leaves observed in (h).

Statistical Information for Extended Data Figures

The data underlying the statistical analyses, together with the results of all pairwise multiple-comparison tests are provided as Supporting Data in the Source Data file.

Extended Data Figure 1 | Group differences in vein connectivity (a,d) and cardinality (c,f) were assessed using one-way ANOVA followed by Tukey's multiple-comparisons test (adjusted P values): (a) $F(8,287) = 210.1$, $P < 0.001$; significant adjusted P values: *d6pkl0* vs. *d6pk01*, **, $P = 0.001$; *d6pkl2* vs. *d6pk01*, **, $P = 0.002$; *d6pk01* vs. *d6pk012*, **, $P = 0.001$; all other comparisons, ***, $P < 0.001$). (c) $F(8,287) = 68.36$, $P < 0.001$; significant adjusted P values: Col vs. *d6pkl1*, *, $P = 0.018$; Col vs. *pax d6pkl1*, **, $P = 0.006$; *d6pkl1* vs. *d6pkl2*, **, $P = 0.007$; $P = 0.006$; *d6pkl2* vs. *pax d6pk01*, **, $P = 0.002$; all other comparisons, ***, $P < 0.001$.(d) $F(11,409) = 334.5$, $P < 0.001$; significant adjusted P values: *pax* vs. D6PKp::YFP-D6PKin *d6pk012*, **, $P = 0.001$; PAXp::YFP-PAX *pax* vs. *d6pk012*, *, $P = 0.020$; PAXp::YFP-PAXin *pax* vs. PAXp::YFP-PAX *pax d6pk012*, **, $P = 0.001$; PAXp::YFP-PAX *pax d6pk012* vs. D6PKp::YFP-D6PKin *d6pk012*, **, $P = 0.002$; all other comparisons, ***, $P < 0.001$. (f) $F(11,409) = 139.3$, $P < 0.001$; significant adjusted P values: *pax* vs. PAXp::YFP-PAX *pax,* **, $P = 0.003$; *d6pk012* vs. D6PKp::YFP-D6PK *d6pk012,* **, $P = 0.002$; *pax d6pk012* vs.D6PKp::YFP-D6PK *pax d6pk012,* **, $P = 0.002$; all other comparisons, ***, $P < 0.001$. Group differences in continuity (b,e) were assessed using the Kruskal–Wallis test followed by Dunn's multiple-comparisons test (adjusted P values): (b) $H(8) = 135.2$, $P < 0.001$; significant adjusted P values: *d6pk012* vs. *pax d6pk01*, **, $P = 0.004$; all other comparisons, ***, $P < 0.001$ (e) $H(11) = 334.1$, $P < 0.001$; all significant comparisons, ***, $P < 0.001$. Sample sizes (n = individual leaves): (a–c) wild type, $n = 48$; *pax*, $n = 51$; *d6pk0*, $n = 45$; *d6pkl1*, $n = 23$; *d6pkl2*, $n = 30$; *d6pk01*, $n = 40$; *d6pk012*, $n = 26$; *pax d6pk01*, $n = 24$; *pax d6pk012*, $n = 9$. (d–f) wild type, $n = 54$; *pax*, $n = 29$; PAXp::YFP-PAX *pax*, $n = 60$; PAXp::YFP-PAXin *pax*, $n = 29$; *d6pk012*, $n = 32$; D6PKp::YFP-D6PK *d6pk012*, $n = 36$; D6PKp::YFP-D6PKin *d6pk012*, $n = 50$; *pax d6pk012*, $n = 15$; PAXp::YFP-PAX *pax d6pk012*, $n = 43$; PAXp::YFP-PAXin *pax d6pk012*, $n = 26$; D6PKp::YFP-D6PK *pax d6pk012*, $n = 21$; D6PKp::YFP-D6PKin *pax d6pk012*, $n = 26$.

Extended Data Figure 4 | (a) Cell axis alignment was quantified for individual cells as absolute deviations from 0° alignment, and group differences were assessed separately at 2.5 D ($H(2) = 4.321$, $P = 0.115$), 3 D ($H(2) = 6.852$, $P = 0.033$), and 4 D ($H(2) = 0.229$, $P = 0.892$) using the Kruskal–Wallis test followed by Dunn's multiple-comparisons test (adjusted P values). Group differences in maximum cell length (b) and cell aspect ratio (c) were assessed using the Kruskal–Wallis test followed by Dunn's multiple-comparisons test (adjusted P values): **(b)** $H(8) = 370.5$, $P < 0.001$; **(c)** $H(8) = 369.4$, $P < 0.001$. Sample sizes (n = individual cells): PAXp::YFP-PAX: 2.5 D, $n = 64$; 3 D, $n = 67$; 4 D, $n = 55$. D6PKp::YFP-D6PK: 2.5 D, $n = 37$; 3 D, $n = 43$; 4 D, $n = 42$. PIN1p::PIN1-GFP: 2.5 D, $n = 51$; 3 D, $n = 51$; 4 D, $n = 61$. Cells were sampled from at least four independent leaves per genotype and developmental stage.

Extended Data Figure 7 | (d) Quantification of mean YFP-PAX pixel intensity in PS and NR cells from 5-day-old primordia grown with or without NPA. Group differences were assessed using one-way ANOVA ($F(3, 26) = 39.42$, $P < 0.001$), followed by Tukey's multiple-comparisons test (all significant pairwise comparisons, $P < 0.001$). Sample sizes (n = individual leaves): mock: PS, $n = 6$; NR, $n = 6$. NPA: PS, $n = 9$; NR, $n = 9$. **(h)** Quantification of procambial tissue width in 5-day-old seedlings treated with NPA or mock. Each dot represents one leaf ($n = 5$ leaves per group), calculated as the mean of five width measurements (technical replicates). Group differences were assessed using one-way ANOVA ($F(5,24) = 42.45$, $P < 0.001$), followed by Tukey's multiple-comparisons test (all

significant pairwise comparisons, *P* < 0.001). **(i)** Cell axis alignment was quantified for individual cells as the absolute deviation from 0° alignment. Group differences were assessed using the Kruskal–Wallis test (H(5) = 164.7, *P* < 0.001), followed by Dunn's multiple-comparisons test (significant adjusted *P* values: wild type mock vs. PAXp::YFP–PAX NPA, *, *P* = 0.048; all other comparisons, ***, *P* < 0.001). **(j,k)** Group differences in maximum cell length (j) and cell aspect ratio (k) were assessed using the Kruskal–Wallis test followed by Dunn's multiple-comparisons test (significant adjusted *P* values). (j) H(8) = 315.4, *P* < 0.001 (significant pairwise comparisons: wild type NPA vs. PAXp::YFP–PAX NPA, **, *P* = 0.006; all other comparisons, ***, *P* < 0.001); (k) H(8) = 244.8, *P* < 0.001 (significant pairwise comparisons: wild type mock vs. wild type NPA, *, *P* = 0.043; PAXp::YFP–PAX mock vs. PAXp::YFP–PAX NPA, **, *P* = 0.002; all other comparisons, ***, *P* < 0.001). Sample sizes (*n* = 80 cells per genotype and treatment; 20 cells were sampled from each of four independent leaves).

Extended Data Figure 9 | (b) Group differences in protein peak width were assessed using the Kruskal–Wallis test followed by Dunn's multiple-comparisons test (adjusted *P* values): *pax* vs. *pax d6pk012* expressing either PAXp::YFP-PAX or PAXp::YFP-PAXin (*H*(3) = 32.97, *P* < 0.001; significant pairwise comparisons: PAXp::YFP–PAXin *pax vs.* PAXp::YFP–PAX *pax d6pk012*, *, *P* = 0.030; all other comparisons, ***, *P* < 0.001), and *d6pk012* vs. *pax d6pk012* expressing either D6PKp::YFP-D6PK or D6PKp::YFP-D6PKin *H*(3) = 12.98, *P* = 0.005; significant pairwise comparison D6PKp::YFP–D*6PK d6pk012 vs.* D6PKp::YFP–D*6PK pax d6pk012*, *, *P* = 0.010). Sample sizes (*n* = individual cells): Sample sizes (*n* = individual cells): PAXp::YFP-PAX *pax* (*n* = 49), PAXp::YFP-PAXin *pax* (*n* = 49), PAXp::YFP-PAX *pax d6pk012* (*n* = 70), PAXp::YFP-PAXin *pax d6pk012* (*n* = 55), D6PKp::YFP-D6PK *d6pk012* (*n* = 38), D6PKp::YFP-D6PKin *d6pk012* (*n* = 39), D6PKp::YFP-D6PK *pax d6pk012* (*n* = 45), D6PKp::YFP-D6PKin *pax d6pk012* (*n* = 43). **(c)** Protein peak orientation were quantified for individual cells as absolute deviations from 0° alignment. Group differences were assessed using the Kruskal–Wallis (*H*(7) = 104.5, *P* < 0.001) test followed by Dunn's multiple-comparisons test (adjusted *P* values; all significant pairwise comparisons, *P* < 0.001). Sample sizes (*n* = individual cells): PAXp::YFP-PAX *pax* (*n* = 51), PAXp::YFP-PAXin *pax* (*n* = 49), PAXp::YFP-PAX *pax d6pk012* (*n* = 72), PAXp::YFP-PAXin *pax d6pk012* (*n* = 56), D6PKp::YFP-D6PK *d6pk012* (*n* = 38), D6PKp::YFP-D6PKin *d6pk012* (*n* = 39), D6PKp::YFP-D6PK *pax d6pk012* (*n* = 45), D6PKp::YFP-D6PKin *pax d6pk012* (*n* = 43). Cells were sampled from at least four independent leaves per genotype (b,c,d).

Supplementary Figure S1 | (b–d) Group differences in vein connectivity (b) and cardinality (d) were assessed using one-way ANOVA followed by Tukey's multiple-comparisons test (adjusted *P* values): (b) *F*(3,202) = 319.4, *P* < 0.001; (d) *F*(3,202) = 1.755, *P* = 0.157. Group differences in continuity (c) were assessed using the Kruskal–Wallis test (*H*(3) = 5.895, *P* = 0.117). Sample sizes (*n* = individual leaves): wild type, *n* = 50; *pax*, *n* = 44; *pax-3*, *n* = 56; PAXp::YFP-PAX *pax*, *n* = 56.

Supplementary Data Figure S4 | (a) Cell axis orientation were quantified for individual cells as absolute deviations from 0° alignment using the Kruskal–Wallis test *H*(17) = 435.9, *P* < 0.001, followed by Dunn's multiple-comparisons test (adjusted *P* values; significant pairwise comparisons: PAXp::YFP–PAXin *pax vs.* PAXp::YFP–PAXin *pax d6pk012*, *, *P* = 0.039; PAXp::YFP–PAXin *pax d6pk012 vs.* D6PKp::YFP–D6PK *pax d6pk012*, *, *P* = 0.034; *pdk1 pdk2 vs.* D6PKp::YFP–D6PK *pax d6pk012*, *, *P* = 0.034 ; all other comparisons, ***, *P* < 0.001). **(c, d)** Group differences in maximum cell length (c) and cell aspect ratio (d) were

assessed using the Kruskal–Wallis test followed by Dunn's multiple-comparisons test (adjusted *P* values: (c) $H(17) = 1015$, $P < 0.001$; (d) $H(17) = 935.6$, $P < 0.001$. Sample sizes (n = individual cells): wild type, n = 71; *pax*, n = 126; PAXp::YFP–PAX *pax*, n = 82; PAXp::YFP–PAXin *pax*, n = 55; *d6pk012*, n = 99; D6PKp::YFP–D6PK *d6pk012*, n = 104 (a), 105 (b), and 105 (c); D6PKp::YFP–D6PKin *d6pk012*, n = 165; *pax d6pk012*, n = 103; PAXp::YFP–PAX *pax d6pk012*, n = 68; PAXp::YFP–PAXin *pax d6pk012*, n = 53; D6PKp::YFP–D6PK *pax d6pk012*, n = 74; D6PKp::YFP–D6PKin *pax d6pk012*, n = 133; *pdk1 pdk2*, n = 84; PDK1p::YFP–PDK1, n = 59 (a) (a), 60 (b), and 60 (c); PDK1p::YFP–PAX, n = 104; PDK1p::YFP–PAX(S596D), n = 85; PDK1p::YFP–D6PK, n = 98; PDK1p::YFP–D6PK(S345D), n = 93. Cells were sampled from at least four independent leaves per genotype.
