# Supplementary material for: PAX-regulated self-organizing polarity drives procambial strand formation
Source: Nat Plants. 2026 Aug 27;12(10):2108–19. doi: 10.1038/s41477-026-02382-w (PMC13680282; doi:10.1038/s41477-026-02382-w)
Supplement: Supplementary file 2 [file 41477_2026_2382_MOESM2_ESM.pdf]

nature portfolio

Corresponding author(s): Claus Schwechheimer NPLANTS-260222325

Last updated by author(s): July 15, 2026

Reporting Summary

Nature Portfolio wishes to improve the reproducibility of the work that we publish. This form provides structure for consistency and transparency in reporting. For further information on Nature Portfolio policies, see our Editorial Policies and the Editorial Policy Checklist.

Statistics

For all statistical analyses, confirm that the following items are present in the figure legend, table legend, main text, or Methods section.

n/a	Confirmed	
☐	☒	The exact sample size (*n*) for each experimental group/condition, given as a discrete number and unit of measurement
☐	☒	A statement on whether measurements were taken from distinct samples or whether the same sample was measured repeatedly
☐	☒	The statistical test(s) used AND whether they are one- or two-sided<br>*Only common tests should be described solely by name; describe more complex techniques in the Methods section.*
☒	☐	A description of all covariates tested
☐	☒	A description of any assumptions or corrections, such as tests of normality and adjustment for multiple comparisons
☐	☒	A full description of the statistical parameters including central tendency (e.g. means) or other basic estimates (e.g. regression coefficient) AND variation (e.g. standard deviation) or associated estimates of uncertainty (e.g. confidence intervals)
☐	☒	For null hypothesis testing, the test statistic (e.g. *F*, *t*, *r*) with confidence intervals, effect sizes, degrees of freedom and *P* value noted<br>*Give P values as exact values whenever suitable.*
☒	☐	For Bayesian analysis, information on the choice of priors and Markov chain Monte Carlo settings
☒	☐	For hierarchical and complex designs, identification of the appropriate level for tests and full reporting of outcomes
☒	☐	Estimates of effect sizes (e.g. Cohen's *d*, Pearson's *r*), indicating how they were calculated

Our web collection on statistics for biologists contains articles on many of the points above.

Software and code

Policy information about availability of computer code

Data collection	n/a
Data analysis	n/a

For manuscripts utilizing custom algorithms or software that are central to the research but not yet described in published literature, software must be made available to editors and reviewers. We strongly encourage code deposition in a community repository (e.g. GitHub). See the Nature Portfolio guidelines for submitting code & software for further information.

Data

Policy information about availability of data

All manuscripts must include a data availability statement. This statement should provide the following information, where applicable:

- Accession codes, unique identifiers, or web links for publicly available datasets
- A description of any restrictions on data availability
- For clinical datasets or third party data, please ensure that the statement adheres to our policy

All original source data for statistical analysis are provided in the source data section of this manuscript. All other data and materials used in the analysis will be made available upon requests addressed to Claus Schwechheimer (claus.schwechheimer@tum.de)

Research involving human participants, their data, or biological material

Policy information about studies with human participants or human data. See also policy information about sex, gender (identity/presentation), and sexual orientation and race, ethnicity and racism.

Reporting on sex and gender	n/a
Reporting on race, ethnicity, or other socially relevant groupings	n/a
Population characteristics	n/a
Recruitment	n/a
Ethics oversight	n/a

Note that full information on the approval of the study protocol must also be provided in the manuscript.

Field-specific reporting

Please select the one below that is the best fit for your research. If you are not sure, read the appropriate sections before making your selection.

☒ Life sciences ☐ Behavioural & social sciences ☐ Ecological, evolutionary & environmental sciences

For a reference copy of the document with all sections, see nature.com/documents/nr-reporting-summary-flat.pdf

Life sciences study design

All studies must disclose on these points even when the disclosure is negative.

Sample size	An a priori power analysis using G*Power was performed to calculate the required sample size, with a target of 95% statistical power and effect size estimates drawn from the literature or pilot studies.
Data exclusions	No data was excluded, except for one case where an outlier was identified. This is stated in the manuscript.
Replication	At least two independent replications were performed for each experiment, yielding comparable results.
Randomization	Randomization was not applied, as the study assessed objective outcomes and plants were grown under uniform growth conditions.
Blinding	Blinding was not applied, as the study assessed objective outcomes and plants were grown under uniform growth conditions.

Reporting for specific materials, systems and methods

We require information from authors about some types of materials, experimental systems and methods used in many studies. Here, indicate whether each material, system or method listed is relevant to your study. If you are not sure if a list item applies to your research, read the appropriate section before selecting a response.

Materials & experimental systems

n/a	Involved in the study	
☐	☒	Antibodies
☒	☐	Eukaryotic cell lines
☒	☐	Palaeontology and archaeology
☒	☐	Animals and other organisms
☒	☐	Clinical data
☒	☐	Dual use research of concern
☐	☒	Plants

Methods

n/a	Involved in the study	
☒	☐	ChIP-seq
☒	☐	Flow cytometry
☒	☐	MRI-based neuroimaging

Antibodies

Antibodies used	The anti-PIN1 S1-P and anti-PIN1 T1-P S1-P antibodies were custom-generated as phospho-specific rabbit polyclonal antibodies raised against the synthetic peptides CGLSATPRPS(PO3H2)NLT and CGLSAT(PO3H2)PRPS(PO3H2)NLT, respectively (Eurogentec, Liège, Belgium). Primary antibodies were used at the following dilutions: 1:1000 for anti-PIN1 rabbit (Abiocode, Agoura Hills, CA, USA);

	1:100 for anti-PIN1 S4-P rabbit (B. Weller et al., 2017); 1:250 for anti-PIN1 S1-P rabbit 1:500 for anti-PIN1 S1-P T1-P rabbit. The secondary antibody used was Cy3-conjugated anti-rabbit IgG (Dianova, Hamburg, Germany) at a dilution of 1:600.
Validation	Antibody specificity for anti-PIN1 S1-P and anti-PIN1 T1-P S1P (this study) was validated by dot blot analysis (see Supplementary Figures), as previously described, using non-phosphorylated, singly phosphorylated, and doubly phosphorylated peptide variants (B. Weller et al., 2017). All other PIN1 phosphosite-specific antibodies were previously described and validated (B. Weller et al., 2017). Information on additional antibodies is available on the manufacturers' websites.

Dual use research of concern

Policy information about dual use research of concern

Hazards

Could the accidental, deliberate or reckless misuse of agents or technologies generated in the work, or the application of information presented in the manuscript, pose a threat to:

No	Yes	
☒	☐	Public health
☒	☐	National security
☒	☐	Crops and/or livestock
☒	☐	Ecosystems
☒	☐	Any other significant area

Experiments of concern

Does the work involve any of these experiments of concern:

No	Yes	
☒	☐	Demonstrate how to render a vaccine ineffective
☒	☐	Confer resistance to therapeutically useful antibiotics or antiviral agents
☒	☐	Enhance the virulence of a pathogen or render a nonpathogen virulent
☒	☐	Increase transmissibility of a pathogen
☒	☐	Alter the host range of a pathogen
☒	☐	Enable evasion of diagnostic/detection modalities
☒	☐	Enable the weaponization of a biological agent or toxin
☒	☐	Any other potentially harmful combination of experiments and agents

Plants

Seed stocks	Detailed information on the sources of seed stocks is provided in the Materials.
Novel plant genotypes	A detailed description of the methods used to generate novel plant genotypes is provided in the Materials.
Authentication	The genotypes included in this study were authenticated through cell biological analyses, genotyping, and phenotypic characterization.
