# Supplementary material for: PAX-regulated self-organizing polarity drives procambial strand formation
Source: Nat Plants. 2026 Aug 27;12(10):2108–19. doi: 10.1038/s41477-026-02382-w (PMC13680282; doi:10.1038/s41477-026-02382-w)
Supplement: Supplementary file 3 [file 41477_2026_2382_MOESM3_ESM.pdf]

nature portfolio

Peer Review File

PAX-REGULATED SELF-ORGANISING POLARITY DRIVES PROCAMBIAL STRAND FORMATION

Corresponding Author: Professor Claus Schwechheimer

This file contains all reviewer reports in order by version, followed by all author rebuttals in order by version.

Version 0:

Reviewer comments:

Reviewer #1

(Comments for the Author)
This study demonstrates that PAX is essential for vascular network connectivity in Arabidopsis thaliana leaves. Loss-of-function mutants of pax and its related genes D6PKs exhibit fragmented veins with reduced connectivity, although vascular cell differentiation still occurs. PAX appears to function through both PIN1-mediated auxin transport and a PIN1-independent mechanism. PAX seems to coordinate procambial strand formation and polarity. Rescue experiments confirm a dominant, kinase-dependent role for PAX in maintaining vein continuity. Live imaging revealed that PAX expression emerges early in morphologically pre-procambial cells, preceding PIN1 polarization and procambial differentiation. Treatment of pax mutants with the auxin transport inhibitor NPA further supports that PAX can act in a PIN1-independent manner, although the underlying molecular mechanism remains unclear at this point.
The data presented are solid, and the images are beautiful, complemented by clear, well-designed cartoons that help the reader follow the findings. These visual elements effectively illustrate the phenotypes and conclusions. However, in addition to the current lack of mechanistic insight, I have several other major concerns that need to be addressed.
Other major comments:
1. I understand that this work is presented as a short letter, and I acknowledge that the finding that PAX acts independently of PIN1 in vein formation is interesting. However, the abstract states that these results “redefine current models of vascular patterning,” which I consider oversold. Several previous studies have already described PIN1-independent mechanisms in vein formation, which are not cited here (see, for example, the recent review: https://www.sciencedirect.com/science/article/pii/S1369526623001267?via=ihub). Molecular players such as van3/SCARFACE, CVP2/CVL1, GNOM, and RPK2 signaling contribute to vascular continuity at least partially independently of polar auxin transport, showing that vein patterning can occur through multiple pathways beyond PIN1. I recommend that the authors place their findings in this context and clarify that their contribution extends existing models, rather than implying that previous models were incomplete or incorrect, thereby presenting the novelty of the PAX-mediated, PIN1-independent pathway in a more balanced and accurate manner.
2. The authors could examine vein defects in cotyledons. This would broaden the impact of the work and allow assessment of whether pax mutants exhibit defects in distal versus proximal vein formation, which seem to be differentially regulated with respect to PIN1 polarity (https://journals.biologists.com/dev/article/149/12/dev200403/275816/A-genetic-framework-for-proximal-secondary-vein).
3. Page 3, Lines 2–3: “The connectivity defects correlate with reduced leaf and plant size, likely reflecting impaired long-distance transport.” It would strengthen the manuscript to compare this observation with other mutants exhibiting fragmented veins and strong growth phenotypes, to support this interpretation with existing literature. Additionally, it would be valuable to directly examine transport in the pax mutant, which displays an intermediate phenotype, for example using dye transport assays, to better support the link between vein connectivity and long-distance transport.
4. Page 5, Lines 1–5: The authors could test the pdk1 pdk2 double mutant under NPA treatment to determine whether these kinases also act through a PIN1-independent mechanism.
Minor comments:
5. Page 1, Line 17: The statement “our findings redefine current models of vascular patterning” is likely an overstatement and could be revised for accuracy (see previous comment about PIN1-independent pathways).

Reviewer #2

(Comments for the Author)
Overall thought: This manuscript reports that the protein kinase PAX plays a role in establishing coordinated cell and tissue polarity during procambial strand formation in Arabidopsis leaves, and proposes that this function is directed by auxin but operates independently of PIN auxin transporters. If true, this would represent a significant advance. The field has a long history of attributing vascular patterning primarily to PIN-mediated polar auxin transport, a view that held for nearly two decades. It was only recently that auxin signaling was identified as an additional essential pathway (PMID: 31793881), and subsequently that plasmodesmata-enabled auxin signal movement (PEASM) was identified as a third pathway – with the simultaneous perturbation of all three pathways recapitulating the gn mutant phenotype of randomly oriented vascular clusters, suggesting that the full set of essential pathways controlling vascular patterning had been identified (PMID: 36166438). Against this backdrop, the identification of a genuinely novel fourth pathway would be a major conceptual development, and I approached this manuscript with that excitement in mind. However, I have several points I would like to bring to the authors' attention, which I believe need to be addressed before the significance of the findings can be fully evaluated.
Conceptual framework and central claims:
The claim that these findings "redefine current models of vascular patterning" and "uncover a novel, auxin-guided, PIN-independent pathway" is one I find genuinely exciting, and I would encourage the authors to engage more explicitly with the existing framework so that readers can fully evaluate whether it is justified.
As I understand it, the current model for vascular patterning includes not just PIN-mediated polar auxin transport, but also auxin signaling and PEASM, with all three pathways acting in coordination under GNOM as master regulator. I would have expected the authors to present this model explicitly in the background, since without it, it is difficult to assess what precisely is being redefined. As written, the framing suggests the field lacks a mechanistic model in this area, which I do not think accurately reflects the published literature.
With this in mind, I was wondering whether showing PIN-independence alone is sufficient to support the claim of a novel pathway. If the three arms of the current model are deeply interconnected and mutually dependent, would it not also be important to examine whether the proposed PAX-mediated function operates independently of auxin signaling and PEASM as well? Neither of which appears to have been addressed experimentally. I was also wondering what the relationship between PAX and GNOM might be – whether PAX acts upstream, downstream, or independently of GNOM seems like important context for evaluating pathway novelty, yet this question is not tackled in the study.
I was also struck by a phenotypic parallel that I think deserves explicit attention. Mutants with defects in plasmodesmata aperture – and therefore impaired PEASM – show mature leaf vascular phenotypes strikingly similar to those of pax;d6pk012: vein fragments, clusters of vascular elements, and in developing leaves, disrupted PIN1 polarity coordination. Importantly, in PEASM-impaired mutants the problem is not simply absent coordination – PIN1 expression domain dynamics are disrupted, with defective narrowing and loss of domain maintenance associated with the vein fragments seen at maturity. I was therefore wondering whether PIN1 expression dynamics in pax; d6pk012 follow a similar pattern, and if so, what this would tell us about the relationship between the proposed PAX pathway and PEASM. Given how striking the phenotypic parallels are, this comparison seems central rather than peripheral to understanding what PAX is actually doing.
There is a broader observation I would invite the authors to consider. As established in (PMID: 31793881), 100 µM NPA – not 25 µM – is required to fully phenocopy the pin sextuple mutant and completely inhibit PIN-dependent vein patterning activity. With this in mind, it is worth noting that in (PMID: 31793881) Figure 10, in wild-type leaves treated with 100 µM NPA, coordinated PIN1-YFP polarity is still eventually achieved, albeit with a delay relative to untreated wild type. This suggests that even in the complete absence of PIN-mediated transport, something else – most plausibly PEASM and/or auxin signaling – can generate the directional cue needed to orient PIN1. The data presented in this manuscript appear to suggest – setting aside my reservations about image quality, which I raise separately – that PAX also remains polarly coordinated under NPA treatment. I was struck by this parallel: both PIN1 and PAX appear to maintain coordinated polarity even when PIN-mediated transport is blocked. I was therefore wondering whether both are responding to the same residual directional signal provided by PEASM and/or auxin signaling, rather than PAX operating through a mechanistically independent pathway. If so, PAX's NPA-insensitive polarity coordination may reflect that both PAX and PIN1 are downstream targets of a shared upstream orienting signal – which would make the relationship between PAX, PEASM, and auxin signaling all the more important to understand.
I raise all of these points not to expand the scope of the study beyond what is feasible, but because I think the authors face a choice. If fully positioning the proposed PAX pathway within the existing framework is outside the scope of what can be addressed experimentally, then the claims in the abstract and summary may need to be reconsidered accordingly. More precisely scoped language about the nature and extent of the finding would, I think, make the manuscript stronger and the claims more defensible.
PAX polarity during procambial cell fate selection:
My concerns about image quality and specific polarity interpretations in Figure 2 panels a–d are detailed in the figure-specific comments – I would encourage the authors to address those first, as the claims in this section rest entirely on that evidence.
Regarding the claim that PAX polarity precedes PIN1 polarization – I found this interesting, and I was wondering where PAX polarity onset falls within the established molecular sequence of preprocambial and procambial marker expression: PIN1 broad expression, then ATHB8 and SHR marking preprocambial state, then Q0990 marking procambial state (PMID: 19710171, PMID: 21128301, PMID: 20563990). Whether the cells showing early PAX polarity are ATHB8-positive or ATHB8-negative would help situate PAX polarity within the known developmental progression and considerably strengthen the interpretation. Additionally, I was wondering whether PAX polarity is maintained normally in pin mutants or under full PIN inhibition – this reciprocal experiment would help evaluate the robustness of the temporal ordering claim.
PAX-dependent cell polarization is independent of PIN-mediated auxin transport:
I noticed an inconsistency between how PIN-independence is framed in the results versus the abstract. In the results, the authors carefully state that aspects of the pax; d6pk012 phenotype "cannot be accounted for by auxin transport defects" – which is measured and defensible. In the abstract this becomes "a novel, auxin-guided, PIN-independent pathway" – a

considerably stronger claim. I would encourage the authors to reconcile these two framings. The topology class of defects in pax;d6pk012 further supports the PEASM parallel raised in the major conceptual comment above.
Discussion
I was struck by an internal tension in principle (ii) – that PAX feeds back on auxin distribution through PIN regulation. If PAX and PIN are coupled in a feedback loop, this reintroduces PIN-dependence through the back door and sits in tension with the PIN-independent pathway claim in the abstract. I would encourage the authors to address this explicitly.
Finally, I noticed that plasmodesmata-mediated signal exchange is mentioned as a speculative future direction. I would encourage the authors to engage more explicitly with the existing PEASM literature here, since as I raise in the major conceptual comment, the phenotypic parallels between pax;d6pk012 and PEASM mutants are striking and seem to deserve more than a passing mention as a future possibility.
Method – Confocal microscopy: The Olympus SZX7 stereomicroscope used for dark-field illumination imaging of cleared leaves is listed under the heading "Confocal microscopy." Please rectify it.
Method – Auxin and inhibitor treatments: The authors use 25 µM NPA to inhibit polar auxin transport in support of their claim that PAX function is PIN-independent. However, as established in (PMID: 31793881), complete phenocopy of the pin sextuple mutant vein pattern requires 100 µM NPA, not 25 µM NPA. The authors should either repeat their experiments at 100 µM NPA or complement their NPA data with appropriate higher-order pin mutant combinations.
Method – Whole-mount immunolocalization: Missing a (;) between "1:250 for anti-PIN1 S1-P rabbit" and "1:500 for anti-PIN1 S1-P T1-P rabbit".
Method – Statistical tests:
Figure S2c: the Kruskal-Wallis test, $H(3) = 6.704$, $p = 0.082$ was insignificant, so I was wondering why post-hoc Dunn's multiple comparisons test was performed?
Figure 2e: (*) was conventionally used to denote $p < 0.05$ instead of $p < 0.01$, so maybe changed to (**)?
For orientation variation, I was wondering why the authors chose to compare group-level median absolute deviations rather than individual cell-level measurements directly?
For all figures:
Lack of reproducibility index information throughout.
Higher-resolution confocal images of regions of interest are needed to unambiguously determine polar localization of PIN1, PAX, and D6PK. Currently, the low-resolution confocal images were not sufficient; the inverted images were helpful in a limited number of cases and uninformative in many others; and the color-coded images with assigned arrows introduced more confusion than clarity in my opinion.
Images of leaves with signs of tissue damage in the region of interest should not be used for polarity analysis and should not be chosen as representative images in figures.
Figure 1:
Panel a: genotype typo.
Figure S1:
Judging visually, there were negative data points in panel c for cardinality index. Mathematically, this should not happen – please double check your calculations.
Panel f: scale typo.
Figure S2:
Lack of consistency in how multiple-gene mutant genotypes were labelled.
pax and pax-3 behaved differently regarding cardinality index, with pax no different from WT and only pax-3 showing a defect. I was therefore wondering why the authors chose to use pax and not pax-3 in other experiments?
I was wondering if genotype labels should be presented differently to not obscure view of leaf features – for example, using a vertical bar with genotype information alongside image panels?
Figure 2:
Lack of description for plots in panel e.
Panel a: the arrows were distracting and obscured view of PAX signal. In at least one case – the central-lower yellow arrow in the bottom rightmost sub-panel – the signal appeared to suggest the opposite of what the arrow implied. A better image should be used here; a reproducibility index is much needed.
Panel b: NLS-VENUS images at 2D, 2.5D, and 3D do not appear to focus on the median plane informative for visualization of developing veins. I suspected the same problem in PAX images at 2D and 2.5D; additionally, 3D and 4D leaves appeared damaged based on signal discontinuity in the midvein and loops. Better images and reproducibility indices are needed.
Panel c: at 2D a bipolar cell appears present in the image yet only basal polarity is indicated in the graphical representation; at 2.5D the image appears to suggest PAX being polarly localized but without coordination, yet coordinated polarity is interpreted. Similar concerns apply at 3D and 4D.
Panel d: with what is presented in these representative images, it is unclear how coordination of PAX polarity could be inferred.
Panel e: were the cells measured vascular cells, and if so, do they belong to the midvein, loops, or minor veins? I was also wondering why group comparisons in statistical tests were done with 2.5D, 3D, and 4D data combined rather than separately for each developmental stage.
Panels f, g: images focusing on loop-forming regions at this magnification would more reliably support PAX polarization analysis – such an approach has been shown to be feasible (PMID: 31793881, Fig. 10).
Figure S4:
Panel a: the leaf appears tilted; merging with the autofluorescence channel may help determine PAX signal boundaries.
Panel b (currently labeled c): judging from the leftmost sub-panel, what is labeled as midvein in the rightmost sub-panel appears to be a parallel vein rather than the true midvein.
Panel c (currently labeled b): the genotype background is unclear. Judging from Figure S3, leaves of these backgrounds are quite WT-looking, suggesting the median plane should capture most developing veins at this stage – making visualization

reliable in principle. However, the different focal planes shown each only partially capture developing cell rows, suggesting suboptimal mounting (see PMID: 35072973 for mounting guidance). With these image quality issues, the polarity interpretations are not convincing.
Figure 3:
Lack of description for plots in panels e, f, g.
2.5D WT: judging from the mid and right sub-panels, the top yellow arrow could equally be drawn toward the neighboring white arrow, leading to a case with no coordinated yellow arrow – consistent with published data (PMID: 36166438, PMID: 31793881) showing that at 2.5D the PIN1 expression domain along the eventual first loop is still broad with no clear polarization.
4D: the arrows were distracting and obscured view of PIN1 antibody signal.
Panels e, f, g: I was wondering why group comparisons were done with 2.5D, 3D, and 4D data combined rather than separately for each developmental stage.
Figure S7:
Presence of many arrows obscuring signal and distracting interpretation throughout.
2.5D (a): same concern as Figure 3 regarding premature polarity assignment. 2.5D (e): the cells captured do not appear to be in the correct median plane, making signal evaluation ambiguous. 2.5D (f): signal appears out of focus – the median plane for vascular cells appears deeper judging from the left sub-panel.
3D (e): the signal on the second loop suggests yellow arrows should point rightward rather than leftward. Cell boundaries are not clearly shown, making polarity interpretation ambiguous.
Figure S8:
Most comments for Figure S7 apply here. Additionally, images in panels j, e, k – and possibly f – appear to show damaged cells at regions of interest, making polarity data unreliable.
Figure S9:
My concern about the NPA concentration used here is detailed in the methods comment and the major conceptual comment above.
Figures S10, S11, S12, S13, S14, and S15:
Most aforementioned comments are applicable here. Additionally:
S13: legend for panel d is missing; please verify whether the same genotype was intended for all four images.
S14: panel b bottom two rows – there appears to be no resemblance between the stained and inverted images and their supposed schematic representations.

Method – Staging of leaf development:
The authors cited (PMID: 36166438) to define developmental landmarks for leaf staging, yet the staging convention used here appears to differ fundamentally from that in the cited reference. In (PMID: 36166438), DAG refers to days after imbibition, with germination beginning upon transfer to light – meaning a leaf labeled 2 DAG comes from a seedling that has been growing for 2 days. By contrast, the authors state that seedlings at 4 DAG produce leaves approximately 2 days old, suggesting that what they label as a 2-day-old leaf would be labeled as a 4 DAG leaf under the convention of the cited reference. This discrepancy is not explained and may reflect differences in growth conditions between the two studies that affect germination timing or early seedling development. Since the developmental landmarks cited – L1 initiation around 2–2.5 days, L2 around 3 days, higher-order veins from approximately 4 days onward – were established under specific conditions and a specific staging convention, the authors should explicitly define their staging system and validate that these landmarks apply under their conditions. The authors may also find it useful to consult the companion protocol paper by the same group (PMID: 35072973), which provides detailed guidance on mounting and imaging very young leaves at the stages relevant to this study.

Reviewer #3

(Comments for the Author)
This manuscript focuses on the relatively unexplored topic of leaf procambial cell formation. While the role of auxin and its directional transport in this process is well described, the mechanisms behind the integration of signalling pathways including gene expression and post-translation modifications remain unclear. In this study, the authors used in vivo and immunofluorescence techniques in various mutant plants to demonstrate the importance of AGC kinases (PAX in particular, but also D6PK), showing that while it is related to auxin levels, it is not primarily linked to the function of PIN auxin transporters. The main evidence comes from analysing the shaping and connectivity of leaf veins in PAX and triple d6pk012 mutant plants; localising the PAX protein kinase in pre-procambial cells; analysing procambial cell alignment and elongation; observing the action of phosphomimetic PAX and upstream kinases PDK; and locally applying auxin, which was unable to stimulate the differentiation of procambial cells.
This original work yields significant results, supported by data, and presented in 4 main figures and 14 (!!!) supplementary images. The quality of the data and its interpretation are of a high standard, and key analyses are very carefully supplemented by statistical analysis. I have several comments that, I hope, will help improve the manuscript with respect to its overall significance, robustness and validity.

Major comments:

1) This work has a very elegant descriptive character, but with respect to the mechanisms involved, one could potentially get more out of it by taking also some analytical approach. For example, it might benefit from the inspiration by studies highlighting the importance of phosphorylation after binding of peptide CLE-type peptides to receptor kinases in leaf vascular development (Hirakawa et al. 2008, PMID: 18812507; Hirakawa et al., 2010, PMID: 20729381). Although this regulation is still not understood in leaf, based on the root results, I would therefore say that the involvement of peptide

hormones through their binding to receptor kinases is very likely closely linked to the activation of a cascade involving the downstream kinase PAX and phosphoinositides, as has been demonstrated in the recent literature (Diaz Ardila et al 2023; Wang et al., 2023, PMID: 36702874; Diaz-Ardila et al., 2023, PMID: 36693368). In roots, this module involves PIN-independent polarization of PAX. Including mutants in the TDIF pathway or its receptor, or testing the role of phosphoinositides, would make this study more conclusive. The main finding presented here is that PAX is polarised by auxin, but not via PIN transporters. While I agree that this is a unique finding with regard to procambium development in the leaf (where vascular cambium is largely not sustained), many of the mechanisms involved in this development are shared between the leaf and the root. Therefore, I believe it would be beneficial to consider this work within the broader context of protein kinase activities involved in auxin-regulated vascular development.

2) Page 3, lines 10-16, authors argued that auxin transport-independent role of PAX could be suggested based on the venation patterns in analyzed mutants. I am afraid that this itself could not support the conclusion that PAX plays auxin transport-independent role here. Data shows that PAX is required, but probably the conclusion that it is required for proper spatiotemporal patterning implies broader or more exclusive role of PAX than it is shown here.

3) Page 4, line 32-33 - The statement "PAX is required for the axial morphogenesis driving procambial strand formation" could be interpreted as implying that PAX is the sole or essential driver of axial morphogenesis, whereas the data show that PAX is necessary for normal axialization but do not exclude contributions from other factors. So perhaps, a bit milder expression could be used, something like "Thus, PAX is required for normal axial morphogenesis during procambial strand formation".

4) Page 4, lines 36-37 - in the sentence "NPA did not affect PAX polarity or PIN1 phosphorylation but broadened PAX and PIN1 expression domains and induced marginal vein expansion, as previously reported (Fig. S9e-h)" the reference on this previous report is missing.

5) Figure 2 - in 2A caption, white arrowheads are not described in the caption. Arrowheads in general could be smaller, because the color-coded localizations could not be almost visible now behind these arrowheads. However, more importantly, it looks like that authors used single marker lines for this analysis, if I am right, while now these images look like that they were generated by co-investigating their patterning in one context. This should be somehow clearly mentioned. Authors want to point attention to the early polarization of PAX, when PIN1 is not showing this type of localization. For this they use color-coding, which is however shown only for PAX, not PIN1. In page 3, line 38, it is stated that PIN1 remained largely diffusive. However, images rather show often quite polarized pattern of PIN1, but very differently from PAX polarization. Moreover, PIN1 is membrane protein and cannot be "diffusive", I would recommend to rephrase this. However, the localization of PAX is indeed very interesting, and it is even consistent with its localization if dividing root protophloem cells, where it is also localized mostly in the middle of the polarized plasma membrane, not along the whole PM (this fact is not discussed in this article). In fig. 2D, the formative cell division is somehow mentioned, but not shown in the scheme. It is obvious from later stages, when cells are elongating, but perhaps for clarity, this could be shown in the scheme if it is mentioned in 2D. Also, I am not sure if "anisotropic elongation is not confusing", already the word „elongation" is clear, perhaps I am not right, but I do not know if elongation could be isotropic, it is always anisotropic.

6) I am slightly confused by the text description of Figure 3 (page 4 starting at line 21). It is mentioned that "PIN1 exhibited coordinated polarity aligned with PAX and D6PK in auxin-accumulating pre-procambial cells." However I did not find any co-staining of PINs with PAX or D6PK, so this needs to be better explained that authors probably argue indirectly based on the evidence from in vivo localizations of PAX in Figure 2 and D6PK in S4. In lines 22-24, in 3c and 3d, there is just phosphorylated PIN1 shown, not PIN1, as mentioned in this sentence.

7) I like data on PDK1, showing its upstream importance for the action of PAX and D6PK. They are presented only in supplementary figures. So perhaps it might be considered, also with respect to their inclusion in the model in Fig. 4, to integrate them in main figures somehow.

8) Considering that in this manuscript there are no other elements of the novel polarization module tested, the statement at page 6, line 12 "our findings uncover a previously unrecognized polarity-guided mechanism that links hormonal gradients to tissue-level organization, offering a new conceptual basis for understanding how vascular networks emerge in the growing plant leaf" possibly overstate the generality and novelty of the mechanism. I am afraid that data support a role for PAX in polarity and tissue organization, but do not fully establish a new conceptual basis. Not so critical, but to mention it here, as shown by Verna et al 2019, also other canonical PINs (3,4,7) and ER PINs, contribute to the leaf vasculature development, while PIN1 is the main player, especially during early development.

Minor comments:
1) The main text, as well as main and supplemental figure panels (sub-figures) are labelled with a mixture of lowercase and uppercase letters. This applies also for many captions, where I found also a mixture of normal and bold letters. This all should be unified.
2) Figure 1a - "awild type" is rather "wild type", panels a and b are not clearly separated, a and b should not be in just one of the images, it should highlight the entire panel a or b. The same could be found in S2 and S10.
3) References 14 and 15 are mentioned in the text on line 27 (page 3), but they rather fit to statement on line 25, and from there, reference 13 should be moved on the line 25.
4) Throughout the manuscript, proteins are labelled inconsistently with uppercase (correct) or uppercase italics (wrong). This should be unified.
5) Page 3, line 28 - probably not "To examine PAX dynamics", but rather "To examine dynamics of PAX distribution".

6) The parameter „protein peak width“ is a bit difficult to understand. If I got it correctly, it is simply the width of the signal of the particular marker. For the evaluation of the polarity, how relevant is “the width”?
7) I really appreciate a lot the quality of methanol-fixed immunostained preparations. In Fig. 3 perhaps instead a-PIN more established abbreviation is α-PIN1.
8) All images in Figure 3a,b,c,d for 2.5 D and 4 D are again shown in S7 (together with 3 D), but from the text this duplication is not clear at first glance.
9) Page 21, line 4, replace “anemorphogenesis” with “and morphogenesis”
10) Fig. S1b and S1e - values on y axes could not be, even theoretically, higher than 1. Therefore, values up to 1.5 are confusing. In the caption of this image, page 16, line 14 - probably not bright field, but transmitted light (considering the setup of confocal microscope).

Version 1:

Reviewer comments:

Reviewer #1

(Comments for the Author)
I think the novelty has now been properly placed within the context of our current knowledge. The possible mechanisms by which PAX controls self-organizing polarity and procambial strand formation are discussed, although they are not formally demonstrated at this stage. At this point, I have no further comments. Once again, congratulations on the beautiful images and the impressive amount of data presented. This is an important piece of work that will significantly move the field forward.

Reviewer #2

(Comments for the Author)
I thank the authors for their thorough and patient responses to my comments.

The revised manuscript and responses address my concerns sufficiently, including more precise framing of the central claim, explicit engagement with the existing framework, and correction of technical and statistical issues. The plasmodesmata parallel is now acknowledged in the discussion.

I support publication in Nature Plants and look forward to seeing how the work is evaluated and built upon by the community.

Reviewer #3

(Comments for the Author)
I appreciate the way the authors revised the manuscript in response to my comments, as well as to the comments of other reviewers. It is clear that the substance of my main comments was consistent with the observations of the other two reviewers, and I am satisfied with how authors addressed them. The manuscript now integrates the results much better into the broader context of vascular development and also provides much greater insight into this issue. I don't have any major objections, and I think the manuscript has been greatly improved.

Open Access This Peer Review File is licensed under a Creative Commons Attribution 4.0 International License, which permits use, sharing, adaptation, distribution and reproduction in any medium or format, as long as you give appropriate credit to the original author(s) and the source, provide a link to the Creative Commons license, and indicate if changes were made.
In cases where reviewers are anonymous, credit should be given to 'Anonymous Referee' and the source.
The images or other third party material in this Peer Review File are included in the article’s Creative Commons license, unless indicated otherwise in a credit line to the material. If material is not included in the article’s Creative Commons license and your intended use is not permitted by statutory regulation or exceeds the permitted use, you will need to obtain permission directly from the copyright holder.
To view a copy of this license, visit https://creativecommons.org/licenses/by/4.0/

Reviewer #1

1.1) General comment: This study demonstrates that PAX is essential for vascular network connectivity in *Arabidopsis thaliana* leaves. Loss-of-function mutants of *pax* and its related genes *D6PKs* exhibit fragmented veins with reduced connectivity, although vascular cell differentiation still occurs. PAX appears to function through both PIN1-mediated auxin transport and a PIN1-independent mechanism. PAX seems to coordinate procambial strand formation and polarity. Rescue experiments confirm a dominant, kinase-dependent role for PAX in maintaining vein continuity. Live imaging revealed that PAX expression emerges early in morphologically pre-procambial cells, preceding PIN1 polarization and procambial differentiation. Treatment of *pax* mutants with the auxin transport inhibitor NPA further supports that PAX can act in a PIN1-independent manner, although the underlying molecular mechanism remains unclear at this point.

The data presented are solid, and the images are beautiful, complemented by clear, well-designed cartoons that help the reader follow the findings. These visual elements effectively illustrate the phenotypes and conclusions. However, in addition to the current lack of mechanistic insight, I have several other major concerns that need to be addressed.

@ 1.1) Response: We thank the reviewer for the positive and thoughtful assessment of our work, as well as for the constructive comments that have helped us improve both the clarity and the positioning of our study. We sincerely feel that the requested revisions bring more clarity and strengthen the manuscript by providing not only a more balanced interpretation of the data but also by positioning our findings better in the context of the existing knowledge.

1.2) Major Comment 1: I understand that this work is presented as a short letter, and I acknowledge that the finding that PAX acts independently of PIN1 in vein formation is interesting. However, the abstract states that these results "redefine current models of vascular patterning," which I consider oversold. Several previous studies have already described PIN1-independent mechanisms in vein formation, which are not cited here (see, for example, the recen treview: https://www.sciencedirect.com/science/article/pii/S1369526623001267?via=ihub). Molecular players such as van3/SCARFACE, CVP2/CVL1, GNOM, and RPK2 signaling contribute to vascular continuity at least partially independently of polar auxin transport, showing that vein patterning can occur through multiple pathways beyond PIN1. I recommend that the authors place their findings in this context and clarify that their contribution extends existing models, rather than implying that previous models were incomplete or incorrect, thereby presenting the novelty of the PAX-mediated, PIN1-independent pathway in a more balanced and accurate manner.

@1.2) Response: We thank the reviewer for this comment. We apologise that our initial framing did not sufficiently acknowledge prior work in this area. It was and is, of course, not our intention to disregard or redefine existing knowledge in the field. As the reviewer correctly states, this is attributed to the word and length limits of this type of article that do not leave much room for introductions or discussions of existing knowledge. We have, however, now adapted the format and generated space, although limited, for a better discussion of our findings.

The emphasis on the PIN-independence of the process was not meant to disregard the fact that there are other proteins that operate in the pathway but are independent of PIN function.

Rather, it derived from the fact that PAX has previously been described as an activator of PINs, which all share polar distribution at the plasma membrane. In the context of procambial strand formation, however, our data shows that PAX polarity precedes PIN polar distribution and that PAX does not require PIN function to become polarised. When viewed from this angle, PAX acts, biochemically and mechanistically, in a PIN-independent manner, but this obviously does not exclude the fact that PIN-mediated auxin transport (and auxin responses) are required for proper vein patterning at the developmental level.

We have revised the manuscript to better separate the roles of PAX and PINs in the process at the biochemical, developmental, and mechanistic levels and to introduce, acknowledge, and discuss previous studies on vascular patterning mechanisms. This has also helped us to more clearly position our findings within the context of this body of work. This is even more important, as there are very obvious hypotheses for future work that can be phrased as to how PAX is regulated by known transcriptional and cell biological processes that are commonly studied in the field. Even more so, we think that PAX has the properties and potential to bridge these molecular players. In line with the reviewer's recommendation, we have clarified how our findings contribute to a broader contextualisation of prior work. We now emphasise that our study adds a mechanistic layer addressing how polarity required for vascular strand formation is established and coordinated at the cellular and tissue levels.

[Abstract] … Here, we identify a previously uncharacterised role for the polar protein PAX in establishing coordinated cell and tissue polarity during this process. We uncover a novel, auxin-guided role for the polar PAX protein in establishing cell and tissue polarity that precedes and is independent of the polarisation and activity of PIN auxin transporters. Our findings refine and redefine existing models of vascular patterning during vein network formation in plant leaves.

[Results] … Together, these findings indicate that PAX is required for proper spatiotemporal patterning of procambial tissue but not for vascular cell differentiation. PAX and D6PK/D6PKLs cooperate to regulate vein continuity and connectivity but are not functionally redundant. Importantly, PAX has critical functions in connecting the vascular network that cannot be explained by the direct regulation of auxin transport by PAX.

[Discussion] … Together, these observations support a dynamic model in which auxin biosynthesis, tissue-wide auxin distribution, and auxin signaling regulate *PAX* transcription and activity during leaf development. These processes are likely mediated, at least in part, through PIN auxin transporters and auxin response pathways. PAX-dependent polarity could then become self-reinforcing, promoting coordinated alignment of polarised cells that ultimately gives rise to continuous procambial strands.

Although our data establish PAX as an important coordinator of vascular polarity, the molecular mechanisms linking auxin perception to PAX function and intercellular polarity propagation remain unresolved. Several previously identified regulators of vascular development may act within this framework. For example, mutants of the auxin response factor *MP* (*MONOPTEROS*) fail to establish a functional leaf vascular system, suggesting that MP may contribute to auxin-responsive regulation of *PAX* expression [15,23].

Among characterised vein-patterning mutants, *sfc/van3* and the *cvp2 cvl* double mutant most closely phenocopy the severe vein fragmentation observed in *pax d6pk012* mutants [24,25]. SFC/VAN3 (SCARFACE/VASCULAR NETWORK DEFECTIVE3) encodes an ADP-ribosylation factor GTPase-activating protein that may facilitate PAX delivery and/or recycling at the plasma membrane [24,26,27]. Similarly, the inositol polyphosphate 5′-phosphatases CVP2 (COTYLEDON VASCULAR PATTERNING2) and CVL (CVP2-LIKE) regulate membrane phosphoinositide composition and could thereby influence PAX stability or retention at the plasma membrane [25]. Such mechanisms are consistent with previous studies demonstrating phosphoinositide-dependent regulation of PAX and other AGC1 kinase family members [10,28,29]. The guanine nucleotide exchange factor GNOM may further contribute to these processes by coordinating trafficking of both PIN auxin transporters and PAX/D6PK family kinases [30,31].

Sterol composition may represent an additional layer of polarity regulation during vascular development. Mutants in the three Arabidopsis *SMT* (*C-24 STEROL METHYLTRANSFERASE*) genes exhibit vascular defects associated with impaired cellular axial organisation [32]. In particular, *cvp1/smt2* and *smt3* are required for the synthesis of downstream C24-ethyl sterols such as 24-ethylidenelophenol [32]. Loss of *SMT2* function in the *cvp1* (*cotyledon vascular patterning 1*) background is further enhanced by *smt3* mutations, and the resulting vascular defects have been associated with impaired cellular axial organisation [32]. These findings suggest that sterol composition contributes to the establishment or maintenance of cell polarity during vascular development, potentially by modulating membrane properties required for coordinated PAX polarity establishment during procambial strand formation.

Our findings also raise the possibility that additional signaling pathways modulate PAX-dependent polarity coordination. BRX family proteins, which interact with PAX during protophloem development in the root, may similarly function during leaf procambial strand formation [7]. Notably, some BRX family proteins act as auxin-sensitive negative regulators of both PAX activity and auxin

transport, suggesting that they may provide feedback control within the auxin–PAX regulatory network [7,33,34]. Peptide-mediated signaling pathways, including CLE (CLAVATA3/EMBRYO SURROUNDING REGION-related) and TDIF (TRACHEARY ELEMENT DIFFERENTIATION INHIBITORY FACTOR) signaling, may likewise participate in the process [29,35-38].

Beyond cell-autonomous mechanisms, PAX may also operate within a plasmodesmata-mediated signaling network. Plasmodesmata aperture is regulated by callose deposition, which controls pore permeability, and both narrowing and widening of plasmodesmata can produce vascular defects including open vein loops, vein fragments, and vascular clusters [39]. The similarity of these phenotypes to those observed in *pax d6pk012* mutants raises the possibility that plasmodesmata-mediated intercellular flux contributes to PAX-dependent coordination of cell and tissue polarity.

1.3) Major Comment 2: The authors could examine vein defects in cotyledons. This would broaden the impact of the work and allow assessment of whether pax mutants exhibit defects in distal versus proximal vein formation, which seem to be differentially regulated with respect to PIN1 polarity (https://journals.biologists.com/dev/article/149/12/dev200403/275816/A-genetic-framework-for-proximal-secondary-vein).

@1.3) Response: We appreciate this suggestion. In fact, our observations were initially made in cotyledons, but we had decided to focus our work on leaf development since this offered a better staging of the process and allowed the inclusion of NPA treatments of the developing leaf and brought us a clearer understanding of the role of auxin transport (inhibition) in this process.

The analysis of vascular networks in cotyledons across the kinase mutant backgrounds revealed defects comparable to those observed in true leaves. Specifically, *pax* mutants exhibit reduced connectivity, *d6pk012* mutants show increased cardinality, and the combined *pax d6pk012* mutant displays vascular differentiation along the midvein, ectopic vascular fragments along the cotyledon margin, and vascular islands scattered throughout the lamina. These cotyledon phenotypes, thereby, closely phenocopy the vascular network defects observed in the leaves. The similarity of these phenotypes suggests that PAX/D6PK-dependent mechanisms function similarly in cotyledons and leaves and that the core principles underlying vascular network formation are largely shared between these two organs. Thus, while the leaf system offered greater experimental resolution and broader opportunities for developmental and pharmacological analyses, the conclusions of our study are very likely extendable to cotyledon vein patterning as well.

We are presenting the available data for this review below (Figure R1).

Regarding the distinct mechanisms proposed for proximal versus distal secondary vein formation and the possible involvement of PAX in these processes, we have not performed the detailed morphometric analysis in *pax* cotyledons that would be required to make a specific statement. As described in the study by the reviewer, this distinction is based on vascular strand morphology and inferred strand origin: distal secondary veins appear to branch directly from pre-existing procambial cell files of the midvein, whereas proximal veins are proposed to arise through a different recruitment process rather than by direct branching. Because this point was specifically raised by the reviewer, we now briefly discuss how both modes of vascular strand morphogenesis are supported by the spatiotemporal deployment and progressive refinement of the specific kinase-mediated cell–cell polarity coordination mechanism uncovered in our work, and point toward future experimental directions that should help resolve these developmental mechanisms in greater detail.

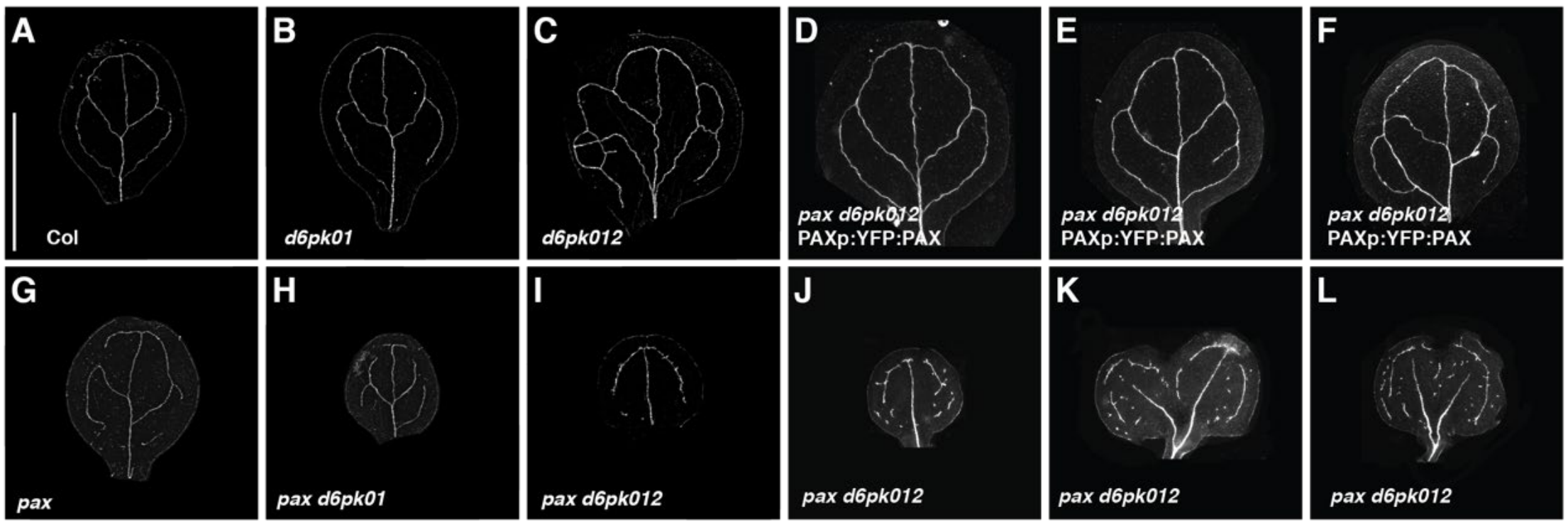

Figure R1. Cotyledon vascular network topology in the *pax* and *d6pk012* mutant panel employed in our study. Representative dark-field microscopy images of cleared cotyledons from 10-day-old seedlings of the indicated genotypes. Scale bar, 2 mm.

1.4) Major Comment 3: Page 3, Lines 2–3: "The connectivity defects correlate with reduced leaf and plant size, likely reflecting impaired long-distance transport." It would strengthen the manuscript to compare this observation with other mutants exhibiting fragmented veins and strong growth phenotypes to support this interpretation with existing literature. Additionally, it would be valuable to directly examine transport in the *pax* mutant, which displays an intermediate phenotype, for example using dye transport assays, to better support the link between vein connectivity and long-distance transport.

@1.4) Response: We agree that this interpretation may potentially require stronger support. At the same time, even if we were to experimentally demonstrate reduced transport in the mutant, this may only be one cause of the reduced growth of the mutant, given the fact that, besides solute transport, polar auxin transport must be strongly impaired in the mutant, too, as well as other as yet unknown kinase-controlled pathways. We now refer to a previous publication where it was shown that the *pax* mutant shows conductivity defects (Marhava et al., 2018).

[Results] The connectivity defects correlated with reduced leaf and plant size, likely reflecting impaired long-distance transport as previously reported for the *pax* mutant (Supplementary Data Figure S1E)[7].

1.5) Major Comment 4: Page 5, Lines 1–5: The authors could test the *pdk1 pdk2* double mutant under NPA treatment to determine whether these kinases also act through a PIN1-independent mechanism.

@1.5) Response: Our motivation for including PDK1 in the analysis was based on the idea that this upstream kinase may act prior to PAX (and D6PK/D6PKL) during procambial strand formation, not only with respect to kinase activation but potentially also in relation to polarity establishment itself. One possibility was that PDK proteins might become polarly localised within future procambial cells and define the polar domain prior to PAX recruitment. However, we do not find evidence that PDK1 or PDK2 themselves are polar proteins at the cellular or tissue level. Instead, our data identify PDK1 activity as necessary for the establishment of kinase-coordinated polarity in ground meristem cells, explicitly through phosphorylation of the activation loop, and for the associated procambial cell axialisation required for procambial strand selection and morphogenesis.

Importantly, we explicitly show that PIN-dependent auxin transport is not required either for PAX polarisation itself or for its function during cell axialisation. Thus, while the reviewer's suggested experiment is technically feasible, it would only reinforce our existing conclusion that PDK acts upstream of the PAX/D6PK polarity module and would therefore not provide substantial additional mechanistic insight beyond the current dataset.

1.6) Minor comment 5 5. Page 1, Line 17: The statement "our findings redefine current models of vascular patterning" is likely an overstatement and could be revised for accuracy (see previous comment about PIN1-independent pathways).

@1.6) Response: We understand the reviewer's concern, which we have addressed as described and shown above (see @1.2).

Reviewer #2

2.1) Overall thought: This manuscript reports that the protein kinase PAX plays a role in establishing coordinated cell and tissue polarity during procambial strand formation in Arabidopsis leaves, and proposes that this function is directed by auxin but operates independently of PIN auxin transporters. If true, this would represent a significant advance. The field has a long history of attributing vascular patterning primarily to PIN-mediated polar auxin transport, a view that held for nearly two decades. It was only recently that auxin signaling was identified as an additional essential pathway (PMID: 31793881), and subsequently that plasmodesmata-enabled auxin signal movement (PEASM) was identified as a third pathway – with the simultaneous perturbation of all three pathways recapitulating the gn mutant phenotype of randomly oriented vascular clusters, suggesting that the full set of essential pathways controlling vascular patterning had been identified (PMID: 36166438). Against this backdrop, the identification of a genuinely novel fourth pathway would be a major conceptual development, and I approached this manuscript with that excitement in mind. However, I have several points I would like to bring to the authors' attention, which I believe need to be addressed before the significance of the findings can be fully evaluated.

@2.1) Response: We thank Reviewer #2 for the thorough and accurate analysis of the current challenges in the field and for highlighting that our findings would represent a significant advance in the field. We particularly appreciate the detailed discussion of the conceptual framework of this expert reviewer.

We are also grateful for the detailed analysis of the figures presented in the paper, which has given us the opportunity to correct several mistakes in the data presentation and analysis that had escaped our attention when preparing the first version of the manuscript.

We would like to clarify that we did not intend to give the impression that PAX acts in an independent or new fourth pathway, and we do not question or redefine the proposed framework of three pathways acting downstream of GNOM. Rather, our contribution is to provide mechanistic insight into the components that regulate cell and tissue polarity during procambial strand and vascular network formation. We propose that this polarity-generating mechanism is spatially deployed through the combined influences of auxin transport, auxin availability within the tissue, and auxin signaling. At present, we do not yet know how this relates to plasmodesmata.

We also hope that we did not give the impression that we were ignorant of other existing observations and the relevant biochemical and developmental landscape. As outlined above in response to a comment from reviewer #1, this is largely explained by the format and brevity of the paper. E.g., the omission of the respective discussion has to be attributed to the fact that the paper in its present form contains a large amount of data that require introduction, data presentation, and discussion. We hope that the paper, in its revised version, where we embed our observation and PAX function in the context of the existing literature, clarifies this point.

The emphasis in our argumentation on the PIN-independent function of PAX is not to be interpreted in the context of the already existing observations that PINs and auxin transport are required for proper vein patterning. Rather, this argumentation is used in view of the biochemical activity of PAX (and D6PK/D6PKLs) and their roles as activators of PINs in other developmental contexts. So, it is less with regard to the developmental process, which requires PINs and auxin transport to shape and reinforce auxin-directed processes, including PAX expression, but rather with regard to the formerly established biochemical role of PAX and D6PK kinases as PIN and auxin transport activators that we consider(ed) this process as being PIN-independent. We have revised the respective sections to make this distinction clearer and hope that the revised paper, also with regard to this point, is acceptable to this and the other reviewers who have phrased similar concerns.

We hope that the revisions address the reviewer's concerns and clarify the significance of our findings.

For the most significant changes in the text, refer to the sections of the revised manuscript presented in response to @1.2.

2.2) Major comment 1: The claim that these findings "redefine current models of vascular patterning" and "uncover a novel, auxin-guided, PIN-independent pathway" is one I find genuinely exciting, and I would encourage the authors to engage more explicitly with the existing framework so that readers can fully evaluate whether it is justified.

As I understand it, the current model for vascular patterning includes not just PIN-mediated polar auxin transport, but also auxin signaling and PEASM, with all three pathways acting in coordination under GNOM as master regulator. I would have expected the authors to present this model explicitly in the background, since without it, it is difficult to assess what precisely is being redefined. As written, the framing suggests the field lacks a mechanistic model in this area, which I do not think accurately reflects the published literature.

@2.2) Response: We appreciate and understand the reviewer's criticism. We have now revised the discussion of the paper and have embedded our observations and the function of PAX in the existing mechanistic framework.

For the most significant changes in the text, refer to the sections of the revised manuscript presented in response to @1.2.

The omission of such a discussion in the first version can be attributed to the length limitations of the article, which, together with the fact that we present many findings that require introduction, presentation, and discussion, left little space for a detailed discussion.

We hope that the revised version of the paper provides a clearer hypothesis as to how PAX and PAX function fit into the existing biochemical and developmental landscape of procambial strand formation. We have tried to do justice to the expectations of the reviewer and kindly ask to view this effort in the context of the severe length constraints that the paper has according to the journal guidelines.

In response, we have revised the abstract, introduction, and discussion to more explicitly present this framework, thereby clarifying the context in which our findings should be interpreted. We have also moderated our wording throughout the manuscript to avoid the implication that the field lacks mechanistic models. Rather than suggesting a redefinition of existing models, we now explicitly emphasise that our findings introduce a mechanistic layer that provides a new perspective to anchor previous work.

2.3) Major comment 2. With this in mind, I was wondering whether showing PIN-independence alone is sufficient to support the claim of a novel pathway. If the three arms of the current model are deeply interconnected and mutually dependent, would it not also be important to examine whether the proposed PAX-mediated function operates independently of auxin signaling and PEASM as well? Neither of which appears to have been addressed experimentally. I was also wondering what the relationship between PAX and GNOM might be – whether PAX acts upstream, downstream, or independently of GNOM seems like important context for evaluating pathway novelty, yet this question is not tackled in the study.

I was also struck by a phenotypic parallel that I think deserves explicit attention. Mutants with defects in plasmodesmata aperture – and therefore impaired PEASM – show mature leaf vascular phenotypes strikingly similar to those of *pax;d6pk012*: vein fragments, clusters of vascular elements, and in developing leaves, disrupted PIN1 polarity coordination. Importantly, in PEASM-impaired mutants the problem is not simply absent coordination – PIN1 expression domain dynamics are disrupted, with defective narrowing and loss of domain maintenance associated with the vein fragments seen at maturity. I was therefore wondering whether PIN1 expression dynamics in *pax; d6pk012* follow a similar pattern, and if so, what this would tell us about the relationship between the proposed PAX pathway and PEASM. Given how striking the phenotypic parallels are, this comparison seems central rather than peripheral to understanding what PAX is actually doing.

@2.3) Response: We thank the reviewer for this important comment and for sharing his observations. We agree that demonstrating PIN-independence alone is not sufficient to justify the definition of a novel, independent pathway, particularly given the established interdependence of PIN-mediated transport, auxin signaling, and plasmodesmata (PEASM) within the GNOM-centered framework in the process.

We have therefore revised the manuscript to clarify that we did not aim to interpret PAX-mediated polarity control as a separate, independent pathway. Rather, our findings introduce a mechanistic layer that provides a new perspective to anchor previous work within a unified framework, in which multiple regulatory processes may converge on the establishment and coordination of AGC1 kinase-dependent tissue polarity control. We also recognise that our previous phrasing and the omission of relevant data may have made it difficult for the reader to fully appreciate this integrative aspect, and we have now revised the manuscript to more clearly present these results and their implications.

For the most significant changes in the text, refer to the sections of the revised manuscript presented in response to @1.2.

In view of the space restrictions and the new experimental complexity and, hence, ample space that the analyses of auxin response, PEASM, and GNOM would bring to our paper, set aside the lengthy period of dedicated experimentation, we can, unfortunately, not include such analyses in this paper, even for the cases where we have already made observations.

Still, we would like to take this opportunity to share some data and observations that the reviewer may find interesting and that show our deeper understanding and appreciation of the underlying observations: With respect to auxin signaling, we have performed experiments demonstrating that PAX polarity and procambial cell axialisation remain intact upon treatment with phenyl-boronic acid (PBA), a drug that was formerly used as an auxin response inhibitor and that also, in our hands, suppresses the activation of the auxin response-dependent DR5:GUS reporter. Our findings, summarised in Figure R2, indicate that the core polarity-establishing mechanism operates also after PBA treatment and thus, possibly, independently of auxin signaling. At the same time we find that auxin signaling is required for the efficient nucleation of new polarity trajectories and for their propagation across neighbouring cells. Consistently, inhibition of auxin signaling results in a reduced and less connected vascular network, characterised by fewer veins and an increased frequency of open vein ends (red circles). In our opinion, these findings do not fall within the conceptual scope of the paper but may become part of follow-up studies that bring the findings reported here in a larger mechanistic context.

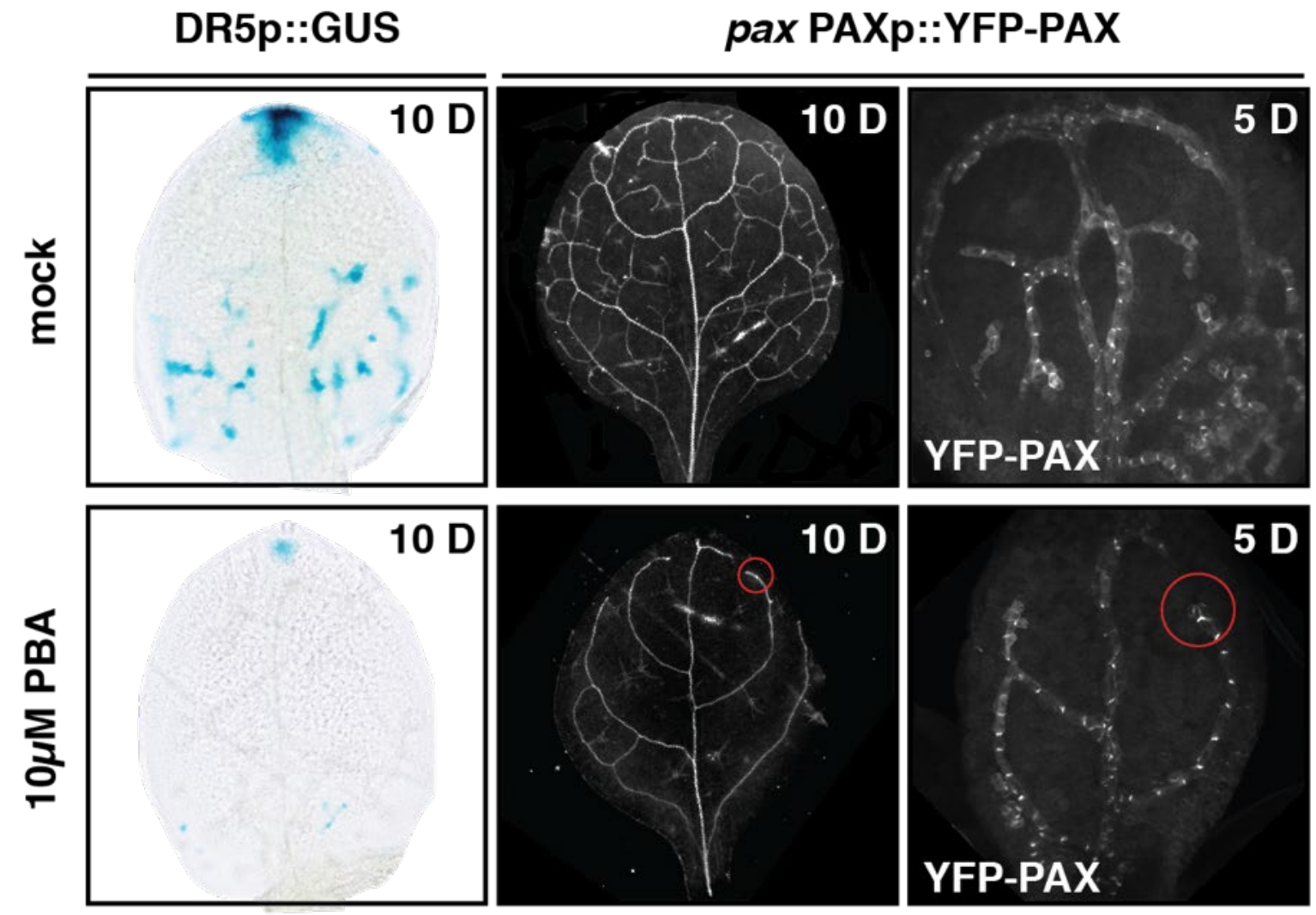

Figure R2: PAX kinase expression dynamics upon phenyl-boronic acid (PBA) treatment. Left panel: DR5p::GUS reporter activity in first leaves of 10-day-old seedlings grown on mock medium (upper) or 10 µM PBA (lower), demonstrating the ability of the PBA treatment to suppress auxin responses. Shown are representative GUS-stained leaves cleared in ethanol and imaged by light stereomicroscopy. **Middle panel:** Representative dark-field microscopy images of cleared first leaves from 10-day-old *pax* seedlings expressing PAXp::YFP-PAX, grown on mock medium (upper) or 10 µM PBA (lower). **Right panel:** Z-projections of representative confocal stacks of 5-day-old leaf primordia expressing PAXp::YFP-PAX in the *pax* mutant background. Red circles highlight the occurrence of open vein ends after PBA treatment.

While our study does not directly address genetic interactions between PEASM and PAX, we agree with the reviewer that notable phenotypic parallels exist between *gsl8* mutants and *pax d6pk012*, particularly with regard to PIN1 polarity convergence in vascular fragments and defects in procambial cell axialisation. Similar phenotypes are also observed in mutants of the ARF-GAP protein *VAN3/SCARFACE* and of *GNOM*, further highlighting a shared class of vascular patterning defects associated with disrupted polarity coordination. In line with our

findings, these phenotypes are consistent with defects in procambial cell patterning that give rise to non-axialised cellular clusters rather than continuous vascular strands and have been difficult to reconcile with classical canalisation models that invoke continuous, sink–source-guided auxin flux gradients as global polarity cues. A common feature of these mutants is the failure of initially broad PIN1 expression domains to progressively refine into continuous, axially polarised procambial strands associated with elevated auxin levels. Instead, PIN1 expression domains resolve into spatially discrete, non-uniform cellular clusters, within which PIN1 polar crescents converge among neighbouring PIN1-polarizing cells, spatially coinciding with sites of auxin accumulation and non-axialised procambial differentiation (vascular islands). These similarities raise the possibility that the regulatory components either act within a shared pathway or converge on common regulatory mechanisms, which future studies will need to resolve.

Regarding the relationship between PAX and GNOM, we agree that this represents an interesting experiment. However, since PIN as well as AGC1 kinase distribution is sensitive to BFA and both types of proteins are transported independently in a GNOM-dependent manner (PMID: 24930721), we would not expect to gain mechanistic insights from analyses in the *gnom* mutant, where we would expect that PINs, PAX, D6PK/D6PKLs, and likely other AGC1 kinases accumulate predominantly in internal compartments.

2.4) Major comment 3: There is a broader observation I would invite the authors to consider. As established in PMID: 31793881, 100 µM NPA – not 25 µM – is required to fully phenocopy the pin sextuple mutant and completely inhibit PIN-dependent vein patterning activity. With this in mind, it is worth noting that in (PMID: 31793881) Figure 10, in wild-type leaves treated with 100 µM NPA, coordinated PIN1-YFP polarity is still eventually achieved, albeit with a delay relative to untreated wild type. This suggests that even in the complete absence of PIN-mediated transport, something else – most plausibly PEASM and/or auxin signaling – can generate the directional cue needed to orient PIN1. The data presented in this manuscript appear to suggest – setting aside my reservations about image quality, which I raise separately – that PAX also remains polarly coordinated under NPA treatment. I was struck by this parallel: both PIN1 and PAX appear to maintain coordinated polarity even when PIN-mediated transport is blocked. I was therefore wondering whether both are responding to the same residual directional signal provided by PEASM and/or auxin signaling, rather than PAX operating through a mechanistically independent pathway. If so, PAX's NPA-insensitive polarity coordination may reflect that both PAX and PIN1 are downstream targets of a shared upstream orienting signal – which would make the relationship between PAX, PEASM, and auxin signaling all the more important to understand.

I raise all of these points not to expand the scope of the study beyond what is feasible, but because I think the authors face a choice. If fully positioning the proposed PAX pathway within the existing framework is outside the scope of what can be addressed experimentally, then the claims in the abstract and summary may need to be reconsidered accordingly. More precisely scoped language about the nature and extent of the finding would, I think, make the manuscript stronger and the claims more defensible.

@2.4) Response: We thank the reviewer for this thoughtful and insightful comment, and for highlighting the relevance of PMID: 31793881. We have revised the manuscript to better position our findings and PAX within the context of the existing literature, and we hope these changes (see response @1.2) address the reviewer's concerns.

We fully agree that PIN-mediated auxin transport and PIN polarity are central to auxin distribution and vein patterning. At the same time, our data indicate that the coordinated cell axialisation of procambial cells is PAX-dependent and that PAX polarity precedes and coordinates axialised PIN1 distribution. Given that PAX regulates PIN activity and that NPA treatment does not disrupt PAX polarisation or the associated alignment of polarised cells, not even quantitatively, we interpret this specific part of the process as being independent of PIN-mediated auxin transport.

Regarding NPA concentrations, we used 25 µM because it is widely employed in the literature to inhibit auxin transport. In PMID: 31793881, the reference mentioned by the reviewer, the authors report that 25 µM and 100 µM NPA treatments produce highly comparable phenotypes, both recapitulating the defects of the *pin sextuple* mutant (Fig. 8h). While we acknowledge that 100 µM NPA has been used in other experimental contexts, including the work referenced by the reviewer, we do not see, based on the data presented in PMID: 31793881, why the higher concentrations may have given stronger phenotypic effects. Further, even if 25 µM NPA was not able to fully block auxin transport, we may have expected to find quantitatively intermediate phenotypes, which is not the case.

[Results] Importantly, *pax d6pk012* failed to form aligned veins <u>in our NPA treatment conditions</u>, and cell elongation defects persisted. <u>We conclude from our observations</u> that, although PAX regulates the coordination of phosphorylated PIN1 polarity and thereby influences auxin distribution, aspects of the *pax d6pk012* phenotype, particularly the loss of vascular cell axialisation, cannot be accounted for by auxin transport defects.

Finally, we appreciate the reviewer's broader point regarding the possibility that both PIN1 and PAX respond to a shared upstream directional cue (e.g., PEASM and/or auxin signaling). While this is an intriguing possibility, directly addressing this question is beyond the scope of the current study. To reflect this, we have been more moderate in our claims and clarified the extent to which our data support a PIN-independent role for PAX.

For the most significant changes in the text, refer to the sections of the revised manuscript presented in response to @1.2.

2.5) Major comment 4: PAX polarity during procambial cell fate selection: My concerns about image quality and specific polarity interpretations in Figure 2 panels a–d are detailed in the figure-specific comments – I would encourage the authors to address those first, as the claims in this section rest entirely on that evidence. Regarding the claim that PAX polarity precedes PIN1 polarization – I found this interesting, and I was wondering where PAX polarity onset falls within the established molecular sequence of preprocambial and procambial marker expression: PIN1 broad expression, then ATHB8 and SHR marking preprocambial state, then Q0990 marking procambial state (PMID: 19710171, PMID: 21128301, PMID: 20563990). Whether the cells showing early PAX polarity are ATHB8-positive or ATHB8-negative would help situate PAX polarity within the known developmental progression and considerably strengthen the interpretation. Additionally, I was wondering whether PAX polarity is maintained normally in pin mutants or under full PIN inhibition – this reciprocal experiment would help evaluate the robustness of the temporal ordering claim.

@2.5) Response: We agree with the reviewer that this experiment would strengthen the temporal resolution of the proposed relationship. While we have not included this marker in the present study, our data show that supracellular PAX polarity domains form as overlapping, multi-trajectory networks prior to, and function upstream of, the delineation of procambial strands, which, based on previous work (Krishna et al., 2021; PMID: 34556094), would be

expected to coincide with *ATHB8* expression. We acknowledge that this is an important direction for future investigation.

2.6) Major comment 5: PAX-dependent cell polarization is independent of PIN-mediated auxin transport: I noticed an inconsistency between how PIN-independence is framed in the results versus the abstract. In the results, the authors carefully state that aspects of the pax; d6pk012 phenotype "cannot be accounted for by auxin transport defects" – which is measured and defensible. In the abstract this becomes "a novel, auxin-guided, PIN-independent pathway" – a considerably stronger claim. I would encourage the authors to reconcile these two framings. The topology class of defects in pax;d6pk012 further supports the PEASM parallel raised in the major conceptual comment above.

@2.6) Response: We thank the reviewer for highlighting this important point. We have now carefully reviewed and reworded the abstract to ensure that "our" biochemical mechanistic interpretation of PAX cellular and tissue polarity being independent from auxin transport and PINs is not interpreted as PINs and auxin transport having no role in the development of the vascular network. As mentioned in several places of this response letter, this requires indeed clarification and stems from our AGC1 kinase-centric viewpoint that these kinases are direct activators of PIN, which does not disregard the fact that it is well established that PIN-mediated auxin transport is required for proper leaf vascular patterning.

We also agree that the vascular topology observed in *pax d6pk012* aligns with the class of phenotypes described for PEASM and related mutants, as already explained above (see @2.3). We have incorporated this perspective into the revised discussion, where we place our findings in the context of the previously described pathways and propose that they may converge on a shared mechanism of tissue polarity coordination.

For the most significant changes in the text, refer to the sections of the revised manuscript presented in response to @1.2.

2.7) Major comment 6: Discussion I was struck by an internal tension in principle (ii) – that PAX feeds back on auxin distribution through PIN regulation. If PAX and PIN are coupled in a feedback loop, this reintroduces PIN-dependence through the back door and sits in tension with the PIN-independent pathway claim in the abstract. I would encourage the authors to address this explicitly.

Finally, I noticed that plasmodesmata-mediated signal exchange is mentioned as a speculative future direction. I would encourage the authors to engage more explicitly with the existing PEASM literature here, since as I raise in the major conceptual comment, the phenotypic parallels between *pax;d6pk012* and PEASM mutants are striking and seem to deserve more than a passing mention as a future possibility.

@2.7) Response: The reviewer is correct in stating that there is an inherent conflict in our statements related to the role of PINs in vascular patterning, which we have resolved for the revised version of the manuscript.

For the most significant changes in the text, refer to the sections of the revised manuscript presented in response to @1.2.

Here are some conclusions on how we understand the role of PAX and PINs in vascular patterning: We demonstrate that coordinated PAX polarity is established prior to and functions upstream of PIN1 polarity in ground-meristem cells, and that its formation is independent of PIN1-mediated polar auxin transport. Instead, PAX polarity requires PAX catalytic activity, the cooperative contribution of D6PK/D6PKLs, and priming phosphorylation by PDK1. While PIN1 activity is dispensable for the establishment of PAX polarity, PIN1-dependent auxin flux is required to progressively refine initially broad PAX expression domains into narrow unicellular files that define pre-procambial strands.

Inhibition of polar auxin transport by NPA results in a marked expansion of the PAX expression domain and, consequently, an enlargement of the field of ground-meristem cells in which coordinated PAX polarity establishes. The altered dynamics of PAX domain organisation closely parallel the changes in auxin distribution observed under conditions of functional versus impaired polar auxin transport. Consistently, local application of exogenous auxin induces local, polarised PAX expression, indicating that tissue-wide auxin distribution modulates PAX expression domain dynamics.

Experiments conducted in the *pax d6pk012* genetic background expressing catalytically inactive PAX (PAXp::YFP-PAXin) reveal the mechanistic nature of this feedback: the PAX expression field initially expands substantially, unlike in lines expressing catalytically active PAX or possessing cooperative D6PK activity, and subsequently fragments into isolated PAX-expressing cell conglomerates at later stages of leaf development. These dynamics closely mirror the aberrant auxin distribution and PIN1 expression patterns observed in *pax d6pk012*, both of which are normally coordinated downstream of PAX function.

In summary, PAX catalytic activity is required for the consolidation of PAX expression into narrow, continuous, axial cell files that subsequently differentiate into procambial strands. The same dependency holds for downstream components: neither PIN1 expression patterns nor auxin distribution along incipient strands are properly established in the absence of PAX (activity). Furthermore, PDK1 promoter–driven PAX and D6PK expression patterns organise into discrete procambial strands only when the kinases are expressed in phosphorylation-mimicking catalytically active forms (D6PK S354D and PAX S596D). Likewise, D6PKin polar domains align along incipient pre-procambial cell strands only in the presence of catalytically active PAX. Together, these data support the existence of a PAX-driven feedback loop, whereby PAX catalytic activity promotes directional PIN-mediated auxin transport, and the auxin flux patterns generated in this way feed back to refine PAX expression boundaries into narrow, axial domains.

We of course fully appreciate the comment on PEASM and have revised the wording and extended the discussion to clarify the potential role of plasmodesmata regulation in the context of our work. The brevity of the article and the fact that there is no (experimental) room to extend our study, in one way or another, unfortunately, restricts the discussion of the PEASM pathway (and that of other regulators in the pathway) to the now-revised discussion, and, again, space constraints allow only a very focused, brief discussion. At the same time, the revised discussion very clearly shows that there is a solid ground for future studies and that very clear hypotheses can be phrased on how PAX interacts not only with PEASM but also with other prominent components of the pathway.

For the most significant changes in the text, refer to the sections of the revised manuscript presented in response to @1.2.

2.7) Minor comments: Method – Confocal microscopy: The Olympus SZX7 stereomicroscope used for dark-field illumination imaging of cleared leaves is listed under the heading "Confocal microscopy." Please rectify it.

@2.7) Response: Thank you for spotting this. We have corrected this.

2.8) Minor comments: Method – Auxin and inhibitor treatments: The authors use 25 μM NPA to inhibit polar auxin transport in support of their claim that PAX function is PIN-independent. However, as established in (PMID: 31793881), complete phenocopy of the pin sextuple mutant vein pattern requires 100 μM NPA, not 25 μM NPA. The authors should either repeat their experiments at 100 μM NPA or complement their NPA data with appropriate higher-order pin mutant combinations.

@2.8) Response (same as @2.4 Response): We thank the reviewer for this thoughtful and insightful comment, and for highlighting the relevance of PMID: 31793881. We have revised the manuscript to better position our findings and PAX within the context of the existing literature, and we hope these changes address the reviewer's concerns.

We fully agree that PIN-mediated auxin transport and PIN polarity are central to auxin distribution and vein patterning. At the same time, our data indicate that the coordinated cell axialisation of procambial cells is PAX-dependent and that PAX polarity precedes and coordinates axialised PIN1 distribution. Given that PAX has been shown to regulate PIN activity and that NPA treatment does not disrupt PAX polarisation or the associated alignment of polarised cells, also not quantitatively, we interpret this specific part of the complex process as being independent of PIN-mediated auxin transport.

Regarding NPA concentrations, we used 25 μM because it is widely employed in the literature to inhibit auxin transport. In PMID: 31793881, the authors report that 25 μM and 100 μM NPA treatments produce very comparable phenotypes, both recapitulating the defects of the *pin sextuple* mutant (Fig. 8h). At the same time, we acknowledge that 100 μM NPA has been used in other experimental contexts, including the work referenced by the reviewer.

Finally, we appreciate the reviewer's broader point regarding the possibility that both PIN1 and PAX respond to a shared upstream directional cue (e.g., PEASM and/or auxin signaling). While this is an intriguing possibility, directly addressing this question is beyond the scope of the current study. To reflect this, we have moderated our claims in the abstract and summary and clarified the extent to which our data support a PIN-independent role for PAX.

For the most significant changes in the text, refer to the sections of the revised manuscript presented in response to @1.2.

2.9) Minor comments: Method – Whole-mount immunolocalization: Missing a (;) between "1:250 for anti-PIN1 S1-P rabbit" and "1:500 for anti-PIN1 S1-P T1-P rabbit".

@2.9) Response: We have corrected this.

2.10) Minor comments: Method – Statistical tests: Figure S2c: the Kruskal-Wallis test, $H(3) = 6.704$, $p = 0.082$ was insignificant, so I was wondering why post-hoc Dunn's multiple comparisons test was performed?

@2.10) Response: We thank the reviewer for this comment. We agree that post-hoc testing is not warranted following a non-significant Kruskal–Wallis result. We have therefore removed Dunn's multiple comparisons test and now report only the Kruskal–Wallis analysis. Figure S2c is Supplementary Figure S1 in the revised manuscript.

2.11) Minor comments: Figure 2e: (*) was conventionally used to denote $p < 0.05$ instead of $p < 0.01$, so maybe changed to (**)?

@2.11) Response: We have corrected this. Thank you for noticing this.

2.12) Minor comments: For orientation variation, I was wondering why the authors chose to compare group-level median absolute deviations rather than individual cell-level measurements directly?

@2.12) Response: We thank the reviewer for raising this point and apologise for the confusing terminology in the figure legend of the original manuscript. Our use of the phrase "median absolute deviations" was not intended to refer to the formal group-level Median Absolute Deviation (MAD) statistic. Rather, we analysed individual cell measurements after transforming each orientation value to its absolute deviation from 0° alignment. We have revised the statistical information section to clarify that cell-level absolute angular deviations were compared among groups using the Kruskal–Wallis test.

This transformation was necessary because peak orientation relative to the procambial cell axis is measured on a symmetric scale ranging from −90° to +90°, with 0° representing alignment to the axis. As a result, opposite deviations of equal magnitude (e.g., −30° and +30°) would cancel each other if analysed directly, causing both the mean and median of the untransformed data to cluster near 0° even when substantial misalignment is present.

By converting values to absolute deviations from 0°, we quantified the magnitude of angular departure from axial alignment independent of direction. These transformed cell-level values were then used for the Kruskal–Wallis test to compare distributions among groups. We have revised the text to clarify this point:

[STATISTICAL INFORMATION] Peak orientation variability was assessed using cell-level absolute deviations from 0° alignment (angle), and group differences were tested using the Kruskal–Wallis test…

2.13) Minor comments: For all figures: Lack of reproducibility index information throughout. Higher-resolution confocal images of regions of interest are needed to unambiguously determine polar localization of PIN1, PAX, and D6PK. Currently, the low-resolution confocal images were not sufficient; the inverted images were helpful in a limited number of cases and uninformative in many others; and the color-coded images with assigned arrows introduced more confusion than clarity in my opinion.

@2.13) Response: Based on this comment, we have reviewed our images and figure files. In our opinion the high-resolution images, which accompany the revised manuscript, clearly show cellular and tissue polarities. We do not believe that providing alternative images would add substantial value. PDF conversion for the original submission may have resulted in a reduction of the resolution. We are also submitting high-resolution figure files for further inspection.

Regarding polarity orientation, it is a common problem that confocal microscopy images do not allow distinguishing between adjacent e.g., the apical and basal membranes, and therefore the often slightly lateralised distribution, the “smile” of the polar signal, is used to attribute a signal to one or the other side of the membrane. We have the feeling that the reviewer is very familiar with this shortcoming and the solution of the problem.

We also would like to underline the importance of the colour-coded images and of the assigned arrows for data interpretation. First, this colour-coding allows visually identifying, in an a priori heterogeneous field of cells and in an unbiased manner, whether there is emerging signal polarity within a cell. Second, the interpretation of the polarity orientation between cells allows judging whether the polarity across the tissue is aligned. Without such an objective assessment of the polarity, which needs to be measured and documented in one way or another, any assessment of the polarity pattern across cells would be subjective. Finally, the arrowheads provide a simplified interpretation of these polarities in the context of strand formation.

We have now included information in the legends and in the statistical information, to provide information about the reproducibility of the findings. At the same time, we would like to draw attention to the high number of quantitative analyses accompanied by statistical analysis provided in the manuscript.

2.14) Minor comments: Images of leaves with signs of tissue damage in the region of interest should not be used for polarity analysis and should not be chosen as representative images in figures.

@2.14) Response: Based on this reviewer’s comment, we have revised all images for signs of tissue damage. We are uncertain which images the reviewer is referring to since we could not identify such cases. We report one genotype, *pax d6pk012*, where the two-dimensional display of the entire leaf is hampered by the fact that the stiffness of the leaf prevents us from “unfolding” it for imaging. Some images, unavoidably, have “shades” of trichomes, which may give the impression of tissue damage.

In figures where we have used lanolin paste for auxin application, we have marked the traces of lanolin paste. This is explained in the legend of the figure and could have been interpreted as a sign of tissue damage.

2.15) Minor comments: Figure 1: Panel a: genotype typo.

@2.15) Response: Thank you. We have corrected this.

2.16) Minor comments: Figure S1: Judging visually, there were negative data points in panel c for cardinality index. Mathematically, this should not happen – please double check your calculations.

@2.16) Response: We thank the reviewer very much for spotting this issue. Upon checking the source files, we found that the position of one data point must have been moved with the remaining items of this panel during figure assembly. We have replaced the panel with the correct plot generated from the source data.

2.17) Minor comments: Figure S1: Panel f: scale typo.

@2.17) Response: Thank you. We have corrected this.

2.18) Minor comments: Figure S2: Lack of consistency in how multiple-gene mutant genotypes were labelled.

@2.18) Response: Thank you. We have corrected this.

Figure S2 is Figure S1 in the revised manuscript.

2.19) Minor comments: pax and pax-3 behaved differently regarding cardinality index, with pax no different from WT and only pax-3 showing a defect. I was therefore wondering why the authors chose to use pax and not pax-3 in other experiments?

@2.19) Response: We thank the reviewer for the question related to the choice of the *pax* allele. The *PAX* loss-of-function allele *pax* (*pax-1*; SAIL_688_B04) was described and established as the reference allele in earlier studies (Marhava and Bassukas et al., 2018). This allele was subsequently used to generate the higher-order mutant combinations and the corresponding complementation lines employed in our work. Therefore, the use of *pax* in the majority of experiments was primarily determined by material availability and the need to maintain consistency with previously established genetic resources.

For the revised manuscript, we performed additional analyses of both *pax* alleles under carefully synchronised developmental conditions, which are shown in Supplementary Figure S1b-d. These new analyses revealed comparable vascular defects in both alleles. Proper staging of developing leaves requires closely matched germination and developmental progression, and we believe that slight differences in developmental synchronisation between the independently performed experiments contributed to the apparent shift in the original data distribution.

To provide the most robust estimate of the phenotype, we combined the newly acquired measurements with the original dataset, as both experiments were performed using the same experimental pipeline, quantification procedure, and statistical criteria. The merged dataset increased the biological sample size and demonstrated consistent phenotypic behavior across independent experiments and both alleles (Supplementary Figure S1b-d of the revised manuscript).

2.20) Minor comments: I was wondering if genotype labels should be presented differently to not obscure view of leaf features for example, using a vertical bar with genotype information alongside image panels?

@2.20) Response: 2.20) Response: Thank you for this helpful suggestion. In preparing the figures, we carefully ensured that genotype labels do not obscure important leaf features. We also considered the use of a dedicated vertical bar for genotype information. Given the high panel density in most figures, this approach would, however, substantially reduce the size of the image panels and consequently diminish the visibility of relevant details.

2.21) Minor comments: Figure 2: Lack of description for plots in panel e.

@2.21) Response: We thank the reviewer for spotting this. We have now added the description of the plots for panel e.

2.22) Minor comments: Figure 2 Panel a: the arrows were distracting and obscured view of PAX signal. In at least one case – the central-lower yellow arrow in the bottom rightmost sub-panel – the signal appeared to suggest the opposite of what the arrow implied. A better image should be used here; a reproducibility index is much needed.

@2.22) Response: We have reviewed this panel and colour-coding of this arrowhead . Thank you for spotting this.

We have responded to issues related to reproducibility in our response to comment 2.13.

2.23) Minor comments: Figure 2 Panel b: NLS-VENUS images at 2D, 2.5D, and 3D do not appear to focus on the median plane informative for visualization of developing veins. I suspected the same problem in PAX images at 2D and 2.5D; additionally, 3D and 4D leaves appeared damaged based on signal discontinuity in the midvein and loops. Better images and reproducibility indices are needed.

@2.23) Response: We thank the reviewer for this comment. All images shown in Figure 2b are z-stacks of optical sections selected to represent the medial plane of the leaves presented. This is discernible from the continuous NLS-VENUS signal visible along the full length of the midvein in the yz projection (see below).

Likewise, in the corresponding PAX images, PAX polarity domains can be followed continuously along the midvein across the entire field of view in all samples, indicating that the relevant medial vascular plane is captured.

We have carefully re-examined the images selected as representative images across multiple biological replicates and independent imaging experiments. We would like to reassure the reviewer that the leaves shown are not damaged.

With regard to reproducibility, we would like to emphasise that the qualitative representation of the PAX signal shown in representative images is also supported by the extensive quantitative analyses included in the paper. To make this clearer, we have now indicated the number of individual leaves measured for polarity quantification analyses, thereby documenting the reproducibility of the observed polarity patterns across biological replicates.

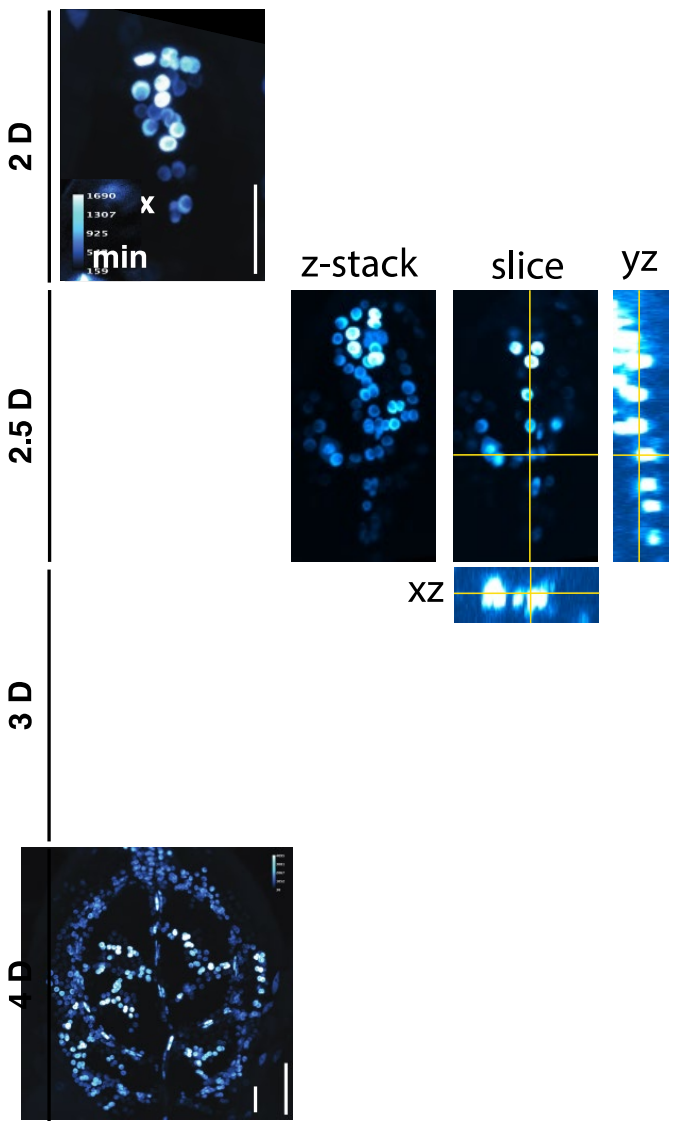

2.24) Minor comments: Figure 2? Panel c: at 2D a bipolar cell appears present in the image yet only basal polarity is indicated in the graphical representation; at 2.5D the image appears to suggest PAX being polarly localised but without coordination, yet coordinated polarity is interpreted. Similar concerns apply at 3D and 4D.

@2.24) Response: We thank the reviewer for this comment and apologise for the confusion caused by the presentation in these panels. We have now incorporated the reviewer's suggestions to better represent what is visible in the images shown and to improve the clarity of the polarity annotations across the developmental stages presented.

2.25) Minor comments: Figure 2? Panel d: with what is presented in these representative images, it is unclear how coordination of PAX polarity could be inferred.

@2.26) Response: We thank the reviewer for this important comment. We hope that, following the changes implemented in panel c, the interpretation of panel d is now also clearer and that the coordination of PAX polarity can be more readily inferred from the representative images shown. We are grateful for the reviewer's suggestions, which substantially improved the clarity and presentation of these panels.

2.26) Minor comments: Figure 2? Panel e: were the cells measured vascular cells, and if so, do they belong to the midvein, loops, or minor veins? I was also wondering why group comparisons in statistical tests were done with 2.5D, 3D, and 4D data combined rather than separately for each developmental stage.

@2.26) Response: We thank the reviewer for this question. All measurements were performed on cells located within the domain for loop 1 (L1). Accordingly, the analyzed cells represent a developmental progression rather than a single mature vascular cell type.

At 2.5 D, cells expressing YFP-PAX, YFP-D6PK, or PIN1-GFP are nearly isodiametric in shape (now Extended Data Figure 4a–c). These cells correspond to ground meristem cells that exhibit potency to adopt either mesophyll or procambial identity.

At 3 D, cells expressing these markers become restricted to narrow cellular files (see Figure 2 a). As quantified in Extended Data Figure 4, they exhibit axial alignment in the range of −30° to +30° relative to the reference incipient strand axis (0°), together with increased cell length and aspect ratio compared with 2.5 D. According to Donner et al., 2009 these cells can be considered as cells in the transient pre-procambial state.

At 4 D, marker-expressing cells display pronounced axial orientation relative to the strand axis and elongated morphology characteristic of procambial cells.

Regarding the statistical analysis of protein peak width, measurements from 2.5 D, 3 D, and 4 D were analyzed across developmental stages to compare the dynamics of YFP-PAX**,** YFP-D6PK, and PIN1-GFP subcellular protein domains throughout the continuous morphogenetic window. This analysis shows that YFP-PAX and YFP-D6PK domains are already sharply defined at early stages and remain comparatively stable, whereas the PIN1 polarity domain remains comparably broad during procambial strand development.

2.27) Minor comments: Panels f, g: images focusing on loop-forming regions at this magnification would more reliably support PAX polarization analysis – such an approach has been shown to be feasible (PMID: 31793881, Fig. 10).

@2.27) Response: We thank the reviewer for this comment. The analyses shown in panel e of Figure 2 were performed in the loop-forming regions of the first loop (L1) at comparable imaging resolution as depicted in the higher-magnification views (HV) shown in panels f and g. We apologise if this was not sufficiently clear from the figure presentation and have revised the legend to state this explicitly.

2.28) Minor comments: Figure S4: Panel a: the leaf appears tilted; merging with the autofluorescence channel may help determine PAX signal boundaries.

@2.28) Response: We thank the reviewer for this comment. We would like to note that former Figure S4a (now Extended Data Figure 3) shows D6PKp::YFP-D6PK expression in the *d6pk012* background. Samples were carefully mounted to preserve the correct orientation of the young primordia, although slight tilting of the leaf structure could not be completely prevented.

Nevertheless, the images shown are z-stack projections of focal scans, which were used to visualise the narrow planar domain in which vascular cell differentiation occurs. We have also added the genotype background to the figure label, as this was not indicated previously.

2.29) Minor comments: Figure S4: Panel b (currently labeled c): judging from the leftmost sub-panel, what is labeled as midvein in the rightmost sub-panel appears to be a parallel vein rather than the true midvein.

@2.29) Response: We thank the reviewer for this careful observation. We have corrected the labeling in Extended Data Figure 3 (formerly Figure S4b). By overlaying the CFW counterstain

with the YFP-PAX signal, we confirmed that the previously indicated cells were incorrectly identified as midvein (MV) cells and instead correspond to parallel vein cells. The figure has been updated accordingly to accurately highlight the MV cells.

2.30) Minor comments: Figure S4: Panel c (currently labeled b): the genotype background is unclear. Judging from Figure S3, leaves of these backgrounds are quite WT-looking, suggesting the median plane should capture most developing veins at this stage – making visualization reliable in principle. However, the different focal planes shown each only partially capture developing cell rows, suggesting suboptimal mounting (see PMID: 35072973 for mounting guidance). With these image quality issues, the polarity interpretations are not convincing.

@2.30) Response: Extended Data Figure 3 was formerly Figure S4, panel c was previously labeled b. We thank the reviewer for this comment and would like to reassure them that all images shown capture the median plane of the respective leaf primordia. We apologise if the presentation gave the impression that these panels represent different focal planes of the same leaf. This is not the case. Rather, the panels show different leaves at the 3 D developmental stage, each focused on the site of L2 formation.

We imaged a series of such leaves and use these representative examples to illustrate the developmental progression of the PAX polarity domain. With reference to the L1 loop visible in the upper part of each magnified region, the uppermost row (which we interpret as the earliest developmental example) shows a broad PAX expression domain at the future L2 position in which PAX has not yet acquired pronounced polar localisation. In the middle row, representing a developmentally more advanced stage, cells proximal to the midvein become polarised and appear to function as polarity attractors for more distally positioned neighbouring cells. In the lowest row, at a later developmental example, many cells within the PAX expression domain exhibit polarity, with multiple partially overlapping PAX polarity trajectories converging on a proximally positioned cell near the midvein, thereby forming a supracellular cone-shaped polarity domain. This supracellular PAX polarity domain precedes the formation of all vein orders that we have examined, demonstrating that it represents a reproducible polarity pattern during vein initiation.

We have revised the legend/text to make clearer that the panels represent different leaves at comparable developmental stages rather than multiple focal planes of a single sample.

2.31) Minor comments: Figure 3: Lack of description for plots in panels e, f, g.

@2.31) Response: We thank the reviewer for noticing this omission. We have now revised the figure legend to explicitly describe the plotted parameters.

2.32) Minor comments: Figure 3: 2.5D WT: judging from the mid and right sub-panels, the top yellow arrow could equally be drawn toward the neighboring white arrow, leading to a case with no coordinated yellow arrow – consistent with published data (PMID: 36166438, PMID: 31793881) showing that at 2.5D the PIN1 expression domain along the eventual first loop is still broad with no clear polarization.

@2.32) We thank the reviewer for this careful and insightful comment. We would like to clarify that in Figure 3 we specifically analyze phosphorylated PIN1 pools detected using the a-PIN1 T1pS1p phospho-specific antibody. In these samples, the signal exhibits polarised localisation, consistent with the patterns observed using the other phospho-specific antibodies tested (a-PIN1 S1p and a-PIN1 S4p; see Extended Data Figure 6 (formerly Fig. S8d,g,j). To better illustrate this, we have provided 3D projections showing that the signals indicated by the yellow and white arrows correspond to distinct polarity domains and are correctly oriented (see below).

At the same time, we agree with the reviewer that total PIN1 localisation at this developmental stage is less sharply resolved. Indeed, both our analysis of PIN1-GFP localisation at 2.5 D in WT (Figure 2e) and the corresponding total a-PIN1 signal shown in Extended Data Figure 6 (formerly Fig. S8a) display subcellular localisation without pronounced overt polarity. This observation is fully consistent with the previously published reports cited by the reviewer (PMID: 36166438; PMID: 31793881). We therefore interpret these data to indicate that the phosphorylated PIN1 fraction already exhibits coordinated polarised enrichment while overall PIN1 polarity is not yet clearly established at 2.5 D, which is the specific feature highlighted in Figure 3 and Extended Data Figures 5 and 6 (formerly Figures S7 and S8).

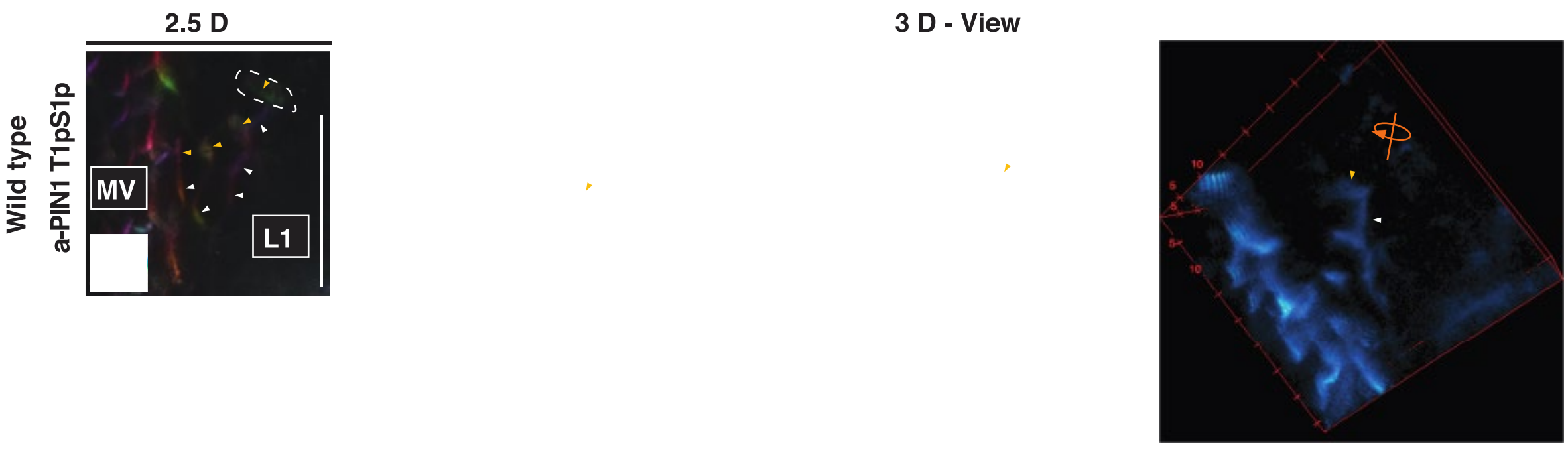

2.33) Minor comments: Figure 4: 4D: the arrows were distracting and obscured view of PIN1 antibody signal.

@2.33) Response: We have now reduced the size of the arrowheads to ensure better visibility of the antibody signals.

2.34) Minor comments: Panels e, f, g: I was wondering why group comparisons were done with 2.5D, 3D, and 4D data combined rather than separately for each developmental stage.

@2.34) Response: We thank the reviewer for this question. We believe that the reviewer refers to panels e, f, and g of Figure 3. As described in our response to comment 2.26, statistical analysis using group comparisons across developmental stages allows comparison of the different genotypes across the continuous developmental window of vascular cell morphogenesis.

2.35) Minor comments: Figure S7: Presence of many arrows obscuring signal and distracting interpretation throughout.

@2.35) Response: We have reduced the size of the arrowheads in Extended Data Figure 5 (formerly Figure S7) and placed them carefully to avoid distraction and improve clarity.

2.36) Minor comments: Figure S7: 2.5D (a): same concern as Figure 3 regarding premature polarity assignment.

@2.36) Response: We thank the reviewer for this comment. We believe that our response to comment 2.32 addresses this concern and clarifies the basis for polarity assignment at the 2.5 D stage in Figure S7a (now Extended Data Figure 5).

2.37) Minor comments: Figure S7: 2.5D (e): the cells captured do not appear to be in the correct median plane, making signal evaluation ambiguous.

@2.37) Response: We thank the reviewer for the considerable care and attention given to examining these images in detail, and we sincerely appreciate this close evaluation. We understand the repeated concern regarding whether individual views precisely capture the medial plane. However, in this context we believe this issue is less critical than assumed, because the polarity patterns analyzed propagate within a continuous unicellular cell sheet and are therefore readily identifiable even when slight deviations in focal position occur.

To account for minor differences caused by mounting or tissue curvature, all images were presented as z-stacks spanning multiple consecutive focal planes, allowing the relevant polarity domains to be captured reliably across the tissue. Moreover, major tilting events would be readily apparent, as polarity domains no longer appear as discrete crescents but instead as broadened or extended signal domains. The panels shown in the figures are representative examples selected from multiple biological replicates and repeated imaging experiments. We therefore remain confident that the signal patterns and polarity interpretations presented in Figure S7e (now Extended Data Figure 5) are robust and valid.

2.38) Minor comments: Figure S7: 2.5D (f): signal appears out of focus – the median plane for vascular cells appears deeper judging from the left sub-panel.

@2.38) Response: We thank the reviewer for this comment. As addressed in our response to comment 2.37, the images are presented as z-stacks of spanning multiple consecutive focal planes to capture the relevant domain. Please note that Figure S7 is now Extended Data Figure 5.

2.39) Minor comments: Figure S7: 3D (e): the signal on the second loop suggests yellow arrows should point rightward rather than leftward. Cell boundaries are not clearly shown, making polarity interpretation ambiguous.

@2.39) Response: We thank the reviewer for this careful comment. After re-examining the image panels, we realised that for figure illustration purposes some panels had inadvertently been reflected after polarity analysis and colour-coding were performed (rather than before), rendering the angular interpretation of the polarity domains inconsistent with the displayed orientation. We have therefore repeated the analysis using the correctly oriented original images and replaced the affected panels accordingly. We thank the reviewer for identifying this issue. As noted above, interpretation of polarity orientation can be challenging in two-dimensional projections; however, the curvature of membrane-associated fluorescent domains provides informative geometric cues that allow reliable inference of polarity direction.

In particular, polarised crescents often appear as characteristic “smile-like” enrichments whose orientation has been successfully used throughout the literature to interpret polarity. Based on these criteria, the reproducibility among multiple biological replicates and repeated imaging experiments, we consider the leftward annotation in Extended Data Figure 5 (formerly Figure S7) 3 D, panel e) to be appropriate.

2.40) Minor comments: Figure S8: Most comments for Figure S7 apply here. Additionally, images in panels j, e, k – and possibly f – appear to show damaged cells at regions of interest, making polarity data unreliable.

@2.40) Response: We thank the reviewer for this comment. As noted in our comments for Figure S7 (now Extended Data Figure 5), the images shown in Figure S8 (now Extended Data Figure 6) are representative examples selected from multiple biological replicates and repeated imaging experiments. We therefore would like to reassure the reviewer that the signal patterns presented in panels j, e, k, and f reflect reproducible observations and are not the result of tissue damage at the regions of interest.

2.41) Minor comments: Figure S9: My concern about the NPA concentration used here is detailed in the methods comment and the major conceptual comment above.

@2.41) Response: As stated in the respective comments made above (@2.4), we have rephrased the respective sections and applied more careful wording to soften our conclusions from these experiments. Figure S9 is now Extended Data Figure 7.

2.42) Minor comments: Figures S10, S11, S12, S13, S14, and S15: Most aforementioned comments are applicable here.

@2.42) Response: We thank the reviewer for these comments. We hope that the concerns raised have been addressed after revision of the figures by improving image presentation, annotations, legends, and the explanation of our imaging and polarity interpretation criteria.

2.43) Minor comments: Additionally: S13: legend for panel d is missing; please verify whether the same genotype was intended for all four images.

@2.43) Response: We thank the reviewer for carefully noticing this omission. We have now added the missing legend for panel d in Extended Data Figure 9 (formerly Figure S13) and properly annotated the genotype assignments for all four images to ensure that they are accurately indicated in the revised figure.

2.44) Minor comments: S14: panel b bottom two rows – there appears to be no resemblance between the stained and inverted images and their supposed schematic representations.

@2.44) Response: We thank the reviewer for this comment. These panels had been correct, but the intention of the panels had maybe been misleading. We have now revised the layout of the figure, which is now main Figure 5, and hope that the revised panel arrangement improves the correspondence between the stained images, inverted images, and their

schematic representations and thereby resolves the confusion noted in the original figure presentation.

2.45) Minor comments: Method – Staging of leaf development: The authors cited (PMID: 36166438) to define developmental landmarks for leaf staging, yet the staging convention used here appears to differ fundamentally from that in the cited reference. In (PMID: 36166438), DAG refers to days after imbibition, with germination beginning upon transfer to light – meaning a leaf labeled 2 DAG comes from a seedling that has been growing for 2 days. By contrast, the authors state that seedlings at 4 DAG produce leaves approximately 2 days old, suggesting that what they label as a 2-day-old leaf would be labeled as a 4 DAG leaf under the convention of the cited reference. This discrepancy is not explained and may reflect differences in growth conditions between the two studies that affect germination timing or early seedling development. Since the developmental landmarks cited – L1 initiation around 2–2.5 days, L2 around 3 days, higher-order veins from approximately 4 days onward – were established under specific conditions and a specific staging convention, the authors should explicitly define their staging system and validate that these landmarks apply under their conditions. The authors may also find it useful to consult the companion protocol paper by the same group (PMID: 35072973), which provides detailed guidance on mounting and imaging very young leaves at the stages relevant to this study.

@2.45) Response: We thank the reviewer for raising this important point. We agree that the terminology used for early leaf staging is, throughout the literature, not fully consistent and may lead to confusion if not explicitly defined. For this reason, we chose to use "D" to indicate "days of leaf age", rather than relying solely on the term DAG (days after germination), a term that has been used differently across different previously published studies:

To avoid ambiguity, we have now clarified our staging system explicitly in the Methods section. We now state that seedlings at 4, 4.5, 5, and 6 days after transfer of sterilised, sown, and stratified seeds to light produce first leaves that are approximately 2, 2.5, 3, and 4 days old, respectively.

To avoid ambiguity, we have now clarified our staging system explicitly in the Methods section.

[Methods] **Staging of leaf development.** Sterilised stratified seeds germinated for 4, 4.5, 5, and 6 days in the light produced first leaves that are approximately 2, 2.5, 3, and 4 days old, respectively. This staging enables the analysis of vein formation events, with loop 1 (L1) typically initiating around 2–2.5 days, loop 2 (L2) around 3 days, and higher-order veins (HV) from approximately 4 days onward [39].

According to Scarpella et al., 2004, "days after germination" (DAG) refer to days after exposure of imbibed seeds to light. Under that convention, after 4 days in light (4 DAG), the first leaf initiates the formation of the first loop, and after 5 days in light (5 DAG), loop 1 is completed while loop 2 initiates (Fig.1; Scarpella et al., 2004; see below). These timings are in good agreement with our observations.

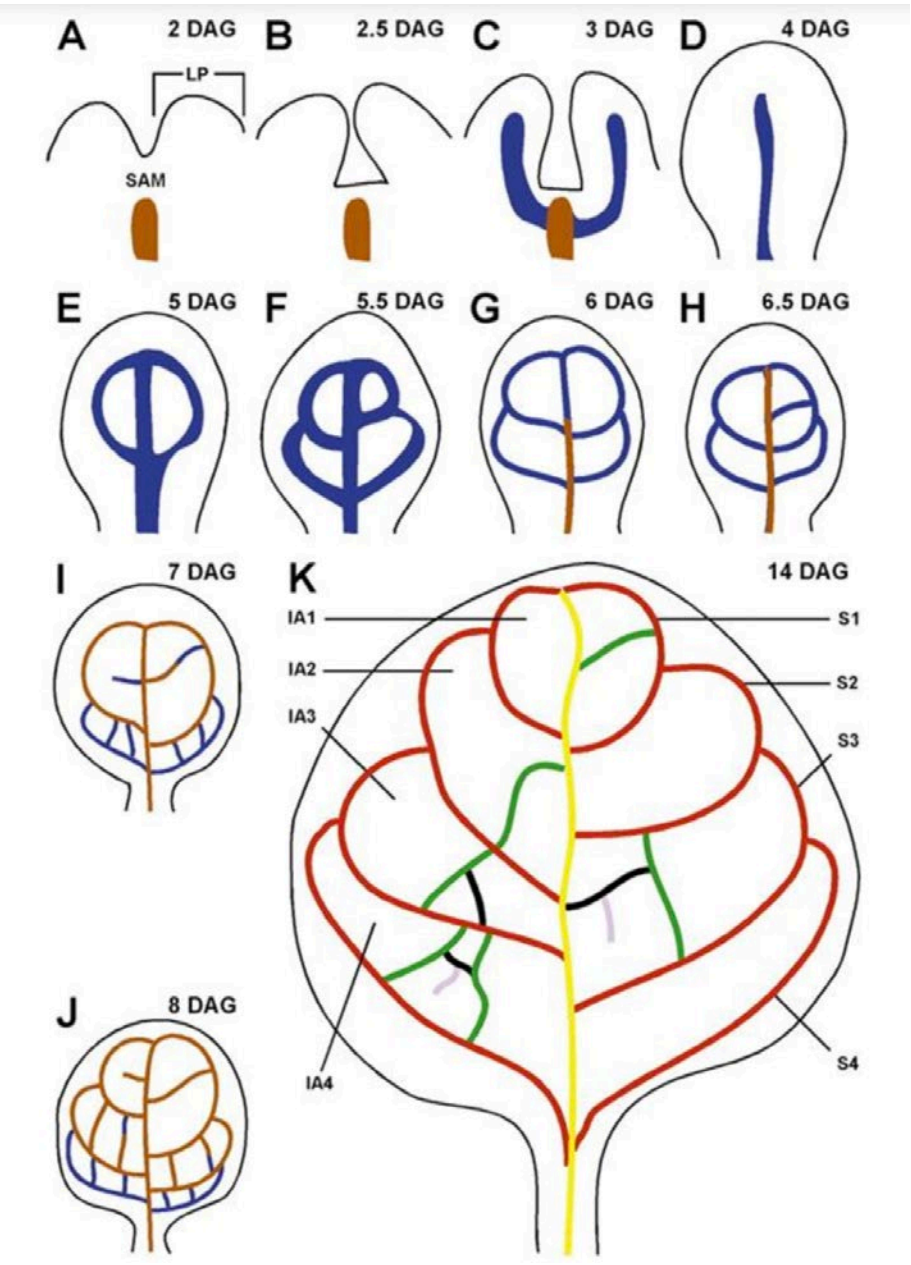

By contrast, in more recent studies (including Verna et al., 2019; PMID: 31793881, and Linh et al.,2022; PMID: 36166438 and PMID: 35072973), the term “2 DAG” appears to be used for days after transfer of sterilised, stratified seeds to light and refers to 4 DAG staged leaves according to Scarpella et al. and to 2 “D“ according to our notation, which differs from the earlier convention and from our empirical developmental timing. For this reason, we adopted the “D” notation to help readers relate our data to the more recent literature while avoiding ambiguity, and we now explain this staging explicitly in the revised manuscript.

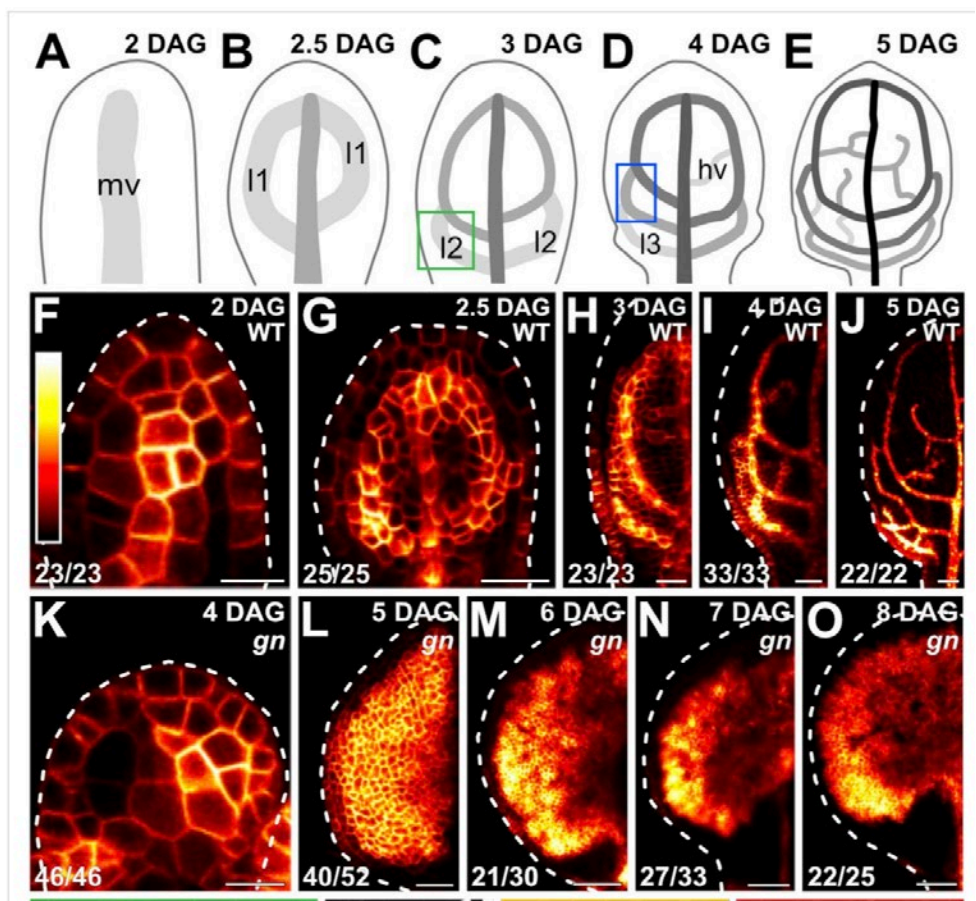

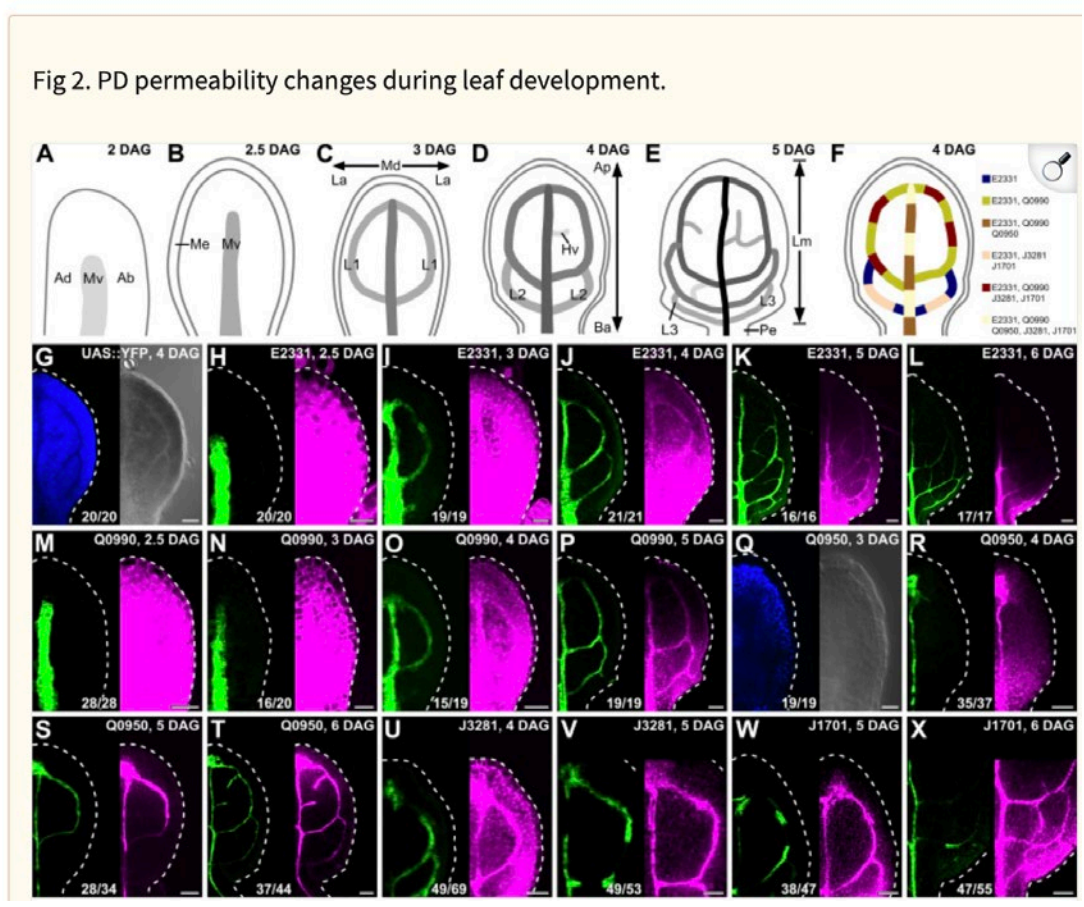

3.1) Reviewer #3 This manuscript focuses on the relatively unexplored topic of leaf procambial cell formation. While the role of auxin and its directional transport in this process is well described, the mechanisms behind the integration of signaling pathways, including gene expression and post-translational modifications, remain unclear. In this study, the authors used in vivo and immunofluorescence techniques in various mutant plants to demonstrate the importance of AGC kinases (PAX in particular, but also D6PK), showing that while it is related

to auxin levels, it is not primarily linked to the function of PIN auxin transporters. The main evidence comes from analysing the shaping and connectivity of leaf veins in PAX and triple d6pk012 mutant plants; localising the PAX protein kinase in pre-procambial cells; analysing procambial cell alignment and elongation; observing the action of phosphomimetic PAX and upstream kinases PDK; and locally applying auxin, which was unable to stimulate the differentiation of procambial cells.

This original work yields significant results, supported by data, and presented in 4 main figures and 14 (!!!) supplementary images. The quality of the data and its interpretation are of a high standard, and key analyses are very carefully supplemented by statistical analysis. I have several comments that, I hope, will help improve the manuscript with respect to its overall significance, robustness and validity.

@3.1) Response. We thank Reviewer #3 for the positive evaluation and constructive suggestions.

3.2) Major comment 1 This work has a very elegant descriptive character, but with respect to the mechanisms involved, one could potentially get more out of it by taking also some analytical approach. For example, it might benefit from the inspiration by studies highlighting the importance of phosphorylation after binding of peptide CLE-type peptides to receptor kinases in leaf vascular development (Hirakawa et al. 2008, PMID: 18812507; Hirakawa et al., 2010, PMID: 20729381). Although this regulation is still not understood in leaf, based on the root results, I would therefore say that the involvement of peptide hormones through their binding to receptor kinases is very likely closely linked to the activation of a cascade involving the downstream kinase PAX and phosphoinositides, as has been demonstrated in the recent literature (Diaz Ardila et al 2023; Wang et al., 2023, PMID: 36702874; Diaz-Ardila et al., 2023, PMID: 36693368). In roots, this module involves PIN-independent polarization of PAX. Including mutants in the TDIF pathway or its receptor, or testing the role of phosphoinositides, would make this study more conclusive. The main finding presented here is that PAX is polarised by auxin, but not via PIN transporters. While I agree that this is a unique finding with regard to procambium development in the leaf (where vascular cambium is largely not sustained), many of the mechanisms involved in this development are shared between the leaf and the root. Therefore, I believe it would be beneficial to consider this work within the broader context of protein kinase activities involved in auxin-regulated vascular development.

@3.2) Response. We appreciate this suggestion. As shown in our response to comment 1.2, we have expanded the Discussion to include connections to position PAX within this broader regulatory context and also make reference, although brief, to peptide signaling, receptor kinases, and phosphoinositide pathways.

[Discussion] Although our data establish PAX as an important coordinator of vascular polarity, the molecular mechanisms linking auxin perception to PAX function and intercellular polarity propagation remain unresolved. Several previously identified regulators of vascular development may act within this framework. For example, mutants of the auxin response factor *MP* (*MONOPTEROS*) fail to establish a functional leaf vascular system, suggesting that MP may contribute to auxin-responsive regulation of *PAX* expression [15,23].

Among characterised vein-patterning mutants, *sfc/van3* and the *cvp2 cvl* double mutant most closely phenocopy the severe vein fragmentation observed in *pax d6pk012* mutants [24,25]. SFC/VAN3 (SCARFACE/VASCULAR NETWORK DEFECTIVE3) encodes an ADP-ribosylation factor GTPase-activating protein that may facilitate PAX delivery and/or recycling at the plasma membrane [24,26,27]. Similarly, the inositol polyphosphate 5′-phosphatases CVP2 (COTYLEDON VASCULAR PATTERNING2) and CVL (CVP2-LIKE) regulate membrane phosphoinositide composition and could thereby influence PAX stability or retention at the plasma membrane [25]. Such mechanisms are consistent with previous studies demonstrating phosphoinositide-dependent regulation of

PAX and other AGC1 kinase family members [10,28,29]. The guanine nucleotide exchange factor GNOM may further contribute to these processes by coordinating trafficking of both PIN auxin transporters and PAX/D6PK family kinases [30,31].

Sterol composition may represent an additional layer of polarity regulation during vascular development. Mutants in the three Arabidopsis *SMT* (*C-24 STEROL METHYLTRANSFERASE*) genes exhibit vascular defects associated with impaired cellular axial organisation [32]. In particular, *cvp1/smt2* and *smt3* are required for the synthesis of downstream C24-ethyl sterols such as 24-ethylidenelophenol [32]. Loss of *SMT2* function in the *cvp1* (*cotyledon vascular patterning 1*) background is further enhanced by *smt3* mutations, and the resulting vascular defects have been associated with impaired cellular axial organisation [32]. These findings suggest that sterol composition contributes to the establishment or maintenance of cell polarity during vascular development, potentially by modulating membrane properties required for coordinated PAX polarity establishment during procambial strand formation.

Our findings also raise the possibility that additional signaling pathways modulate PAX-dependent polarity coordination. BRX family proteins, which interact with PAX during protophloem development in the root, may similarly function during leaf procambial strand formation [7]. Notably, some BRX family proteins act as auxin-sensitive negative regulators of both PAX activity and auxin transport, suggesting that they may provide feedback control within the auxin–PAX regulatory network [7,33,34]. Peptide-mediated signaling pathways, including CLE (CLAVATA3/EMBRYO SURROUNDING REGION-related) and TDIF (TRACHEARY ELEMENT DIFFERENTIATION INHIBITORY FACTOR) signaling, may likewise participate in the process [29,35-38].

3.3) Major comment 2 Page 3, lines 10-16, authors argued that auxin transport-independent role of PAX could be suggested based on the venation patterns in analyzed mutants. I am afraid that this itself could not support the conclusion that PAX plays auxin transport-independent role here. Data shows that PAX is required, but probably the conclusion that it is required for proper spatiotemporal patterning implies broader or more exclusive role of PAX than it is shown here.

@3.3) Response. We thank the reviewer for this important comment and agree that phenotypic observations alone are not sufficient to establish independence from auxin transport. We would like to emphasise, however, that we do not solely base our conclusion on vein patterning but provide robust quantitative evidence showing that PAX functions in cell axialisation independently of PIN-dependent auxin transport.

As already stated in other, related comments, we have now clarified the interpretation of our data in a broader developmental context (see our response to 3.2 above). It is, of course, not the case that we exclude the role of auxin responses and auxin transport in the developmental process, but it is rather the precise developmental process of coordinated cell axialisation, which is likely independent of PINs (see comments on the NPA concentration used for PIN inhibition above: @2.4) and PAX-dependent PIN activation, which we consider PIN-independent. So, essentially, our conclusion and wording come from the emphasis on the direct biochemical role of PAX rather than from its developmental role in the broader context of leaf vascular development.

All reviewers have commented on this apparent discrepancy within our manuscript and the apparent conflict with published data and knowledge, which we can understand. We have now carefully reworded the article and hope that, in its revised form, the specific individual contribution of PAX to vascular patterning becomes clear. See also our comments on point 1.2 by reviewer #1.

3.4) Major comment 3. Page 4, line 32-33 - The statement “PAX is required for the axial morphogenesis driving procambial strand formation” could be interpreted as implying that PAX is the sole or essential driver of axial morphogenesis, whereas the data show that PAX is necessary for normal axialization but do not exclude contributions from other factors. So

perhaps, a bit milder expression could be used, something like "Thus, PAX is required for normal axial morphogenesis during procambial strand formation".

@3.4) Response: Thank you very much for this comment. We have now revised the article such that we put PAX in a broader developmental context and phrase hypotheses about its possible function in the context of other developmental players. See our comments in 1.2, 3.2 and 3.3.

3.5) Major comment 4. Page 4, lines 36-37 - in the sentence "NPA did not affect PAX polarity or PIN1 phosphorylation but broadened PAX and PIN1 expression domains and induced marginal vein expansion, as previously reported (Fig. S9e-h)" the reference on this previous report is missing.

@3.5) Response: We apologise for this omission and have now added the respective reference for the effects of NPA on PIN1 (Verna et al., 2019).

3.6) Major comment 5 Figure 2 – in 2A caption, white arrowheads are not described in the caption. Arrowheads in general could be smaller, because the color-coded localizations could not be almost visible now behind these arrowheads. However, more importantly, it looks like that authors used single marker lines for this analysis, if I am right, while now these images look like that they were generated by co-investigating their patterning in one context. This should be somehow clearly mentioned. Authors want to point attention to the early polarization of PAX, when PIN1 is not showing this type of localization. For this they use color-coding, which is however shown only for PAX, not PIN1. In page 3, line 38, it is stated that PIN1 remained largely diffusive. However, images rather show often quite polarized pattern of PIN1, but very differently from PAX polarization. Moreover, PIN1 is membrane protein and cannot be "diffusive", I would recommend to rephrase this. However, the localization of PAX is indeed very interesting, and it is even consistent with its localization if dividing root protophloem cells, where it is also localized mostly in the middle of the polarized plasma membrane, not along the whole PM (this fact is not discussed in this article). In fig. 2D, the formative cell division is somehow mentioned, but not shown in the scheme. It is obvious from later stages, when cells are elongating, but perhaps for clarity, this could be shown in the scheme if it is mentioned in 2D. Also, I am not sure if "anisotropic elongation is not confusing", already the word „elongation" is clear, perhaps I am not right, but I do not know if elongation could be isotropic, it is always anisotropic.

@3.6 Response: We have improved figure clarity, reduced arrowhead sizes, clarified labeling, and revised text descriptions to avoid ambiguity.

The term "anisotropic elongation" is indeed better than the term "elongation" because it describes a directionality of the cellular elongation.

3.7) Major comment 6 I am slightly confused by the text description of Figure 3 (page 4 starting at line 21). It is mentioned that "PIN1 exhibited coordinated polarity aligned with PAX and D6PK in auxin-accumulating pre-procambial cells." However I did not find any co-staining of PINs with PAX or D6PK, so this needs to be better explained that authors probably argue indirectly based on the evidence from in vivo localizations of PAX in Figure 2 and D6PK in S4.

In lines 22-24, in 3c and 3d, there is just phosphorylated PIN1 shown, not PIN1, as mentioned in this sentence.

@3.7) Response: We thank the reviewer for spotting these inaccuracies in the text, which have now been corrected in the text.

3.8) Major comment 7 I like data on PDK1, showing its upstream importance for the action of PAX and D6PK. They are presented only in supplementary figures. So perhaps it might be considered, also with respect to their inclusion in the model in Fig. 4, to integrate them in main figures somehow.

@3.8) Response: We thank the reviewer for this positive comment and for highlighting the importance of the PDK1 data. We agree that these findings are mechanistically important, particularly because they place PDK1 upstream of coordinated PAX and D6PK polarity regulation and therefore directly support the model presented in Figure 6.

In response to the reviewer's suggestion, we have now incorporated the PDK1 analysis as Figure 4 in the revised manuscript. This improves the conceptual continuity of the manuscript and more directly integrates our findings with the proposed mechanistic model. We thank the reviewer for this helpful suggestion.

3.8) Major comment 8 Considering that in this manuscript there are no other elements of the novel polarization module tested, the statement at page 6, line 12 "our findings uncover a previously unrecognized polarity-guided mechanism that links hormonal gradients to tissue-level organization, offering a new conceptual basis for understanding how vascular networks emerge in the growing plant leaf" possibly overstate the generality and novelty of the mechanism. I am afraid that data support a role for PAX in polarity and tissue organization, but do not fully establish a new conceptual basis. Not so critical, but to mention it here, as shown by Verna et al 2019, also other canonical PINs (3,4,7) and ER PINs, contribute to the leaf vasculature development, while PIN1 is the main player, especially during early development.

@3.8 Response. We appreciate the comment made by the reviewer and do fully agree to their judgment. As stated elsewhere in this response letter, we have now revised the paper to make clear that the PAX-mediated cell-to-cell alignment during procambial strand formation is the conceptual novelty of our work and that we refer to this process when we state that PAX acts (biochemically) in a PIN-independent manner. We hope that the revised manuscript better clarifies this point. See our response to comment 1.2 for the revisions of the respective text sections.

With regard to a similar comment from Reviewer #2, we have now softened the conclusions that PINs are not involved in this process, since we cannot rule out that residual PIN activity is still present in cells even when treated with 25 µM NPA.

Minor comments

3.9) Minor comments 1) The main text, as well as main and supplemental figure panels (sub-figures) are labelled with a mixture of lowercase and uppercase letters. This applies also for

many captions, where I found also a mixture of normal and bold letters. This all should be unified.

@3.9) Response: We apologise for this and have revised all figures to ensure that we use uniform letter labels.

3.10) Minor comments 2) Figure 1a - “awild type” is rather “wild type”, panels a and b are not clearly separated, a and b should not be in just one of the images, it should highlight the entire panel a or b. The same could be found in S2 and S10.

@3.10) Response: We apologise for this and have revised the figure layout and corrected the typo.

3.11) Minor comments 3) References 14 and 15 are mentioned in the text on line 27 (page 3), but they rather fit to statement on line 25, and from there, reference 13 should be moved on the line 25.

@3.11) Response: We apologise for this. We have revised the reference listing in this section.

3.12) Minor comments 4) Throughout the manuscript, proteins are labelled inconsistently with uppercase (correct) or uppercase italics (wrong). This should be unified.

@3.12) Response: We apologise for this and have revised the manuscript to ensure the proper labeling for proteins and genes in accordance with the rules of the community. Since we are generally referring to observations of proteins, we use predominantly upper case letters. Please also note that we treat transgenes as proper names (and not as gene names as others may do) and therefore do not use italics to refer to them.

3.13) Minor comments 5) Page 3, line 28 - probably not “To examine PAX dynamics”, but rather “To examine dynamics of PAX distribution”.

@3.13) Response: This has been corrected. Thank you.

3.14) Minor comments 6) The parameter „protein peak width“ is a bit difficult to understand. If I got it correctly, it is simply the width of the signal of the particular marker. For the evaluation of the polarity, how relevant is “the width”?

@3.14) Response: We thank the reviewer for this question. The parameter protein peak width refers to the spatial width of the membrane-associated fluorescence peak of the respective marker. This measurement is based on a fitted Gaussian curve and therefore provides an approximation of the underlying peak width. We included this parameter because it offers relative quantitative information on the degree of spatial confinement versus lateral spread of polarised protein domains, and thus complements the polarity orientation measurements.

For example, in Figure 2e, comparison of protein peak width across developmental stages allowed us to assess the dynamics of YFP-PAX, YFP-D6PK, and PIN1-GFP subcellular polarity domains throughout the morphogenetic window of procambial strand development. This analysis shows that YFP-PAX and YFP-D6PK polarity domains are already sharply

defined at the early 2.5 D stage and remain comparatively stable, whereas the PIN1 polarity domain remains comparatively broader throughout procambial strand development.

3.15) Minor comments 7) I really appreciate a lot the quality of methanol-fixed immunostained preparations. In Fig. 3 perhaps instead a-PIN more established abbreviation is α-PIN1.

@3.15) Response: We have now replaced the a- by anti- to refer to the antibodies used for immunostaining. Adobe Illustrator does not allow displaying α in symbol font as bold letter.

3.16) Minor comments 8) All images in Figure 3a,b,c,d for 2.5 D and 4 D are again shown in S7 (together with 3 D), but from the text this duplication is not clear at first glance.

@3.16) Response: We have now revised the text to clarify this.

3.17) Minor comments 9) Page 21, line 4, replace "anemorphogenesis" with "and morphogenesis"

@3.17) Response: Thank you. We have corrected this mistake.

3.18) Minor comments 10) Fig. S1b and S1e - values on y axes could not be, even theoretically, higher than 1. Therefore, values up to 1.5 are confusing. In the caption of this image, page 16, line 14 - probably not bright field, but transmitted light (considering the setup of confocal microscope).

@3.18) Response: This comment is accurate and we have now removed the values greater than 1 from the y-axes in Extended Data Figure 1 (formerly Figure S1). We also specified that the images were acquired using transmitted light rather than bright-field microscopy. We thank the reviewer for these helpful corrections.
